# Supplementary material for: Dimensionality reduction methods for extracting functional networks from large‐scale CRISPR screens
Source: Mol Syst Biol. 2023 Sep 26;19(11):e11657. doi: 10.15252/msb.202311657 (PMC10632734; doi:10.15252/msb.202311657)
Supplement: Supplementary file 1 — Appendix S1 [file MSB-19-e11657-s001.pdf]

Appendix Materials

Manuscript Title

Dimensionality reduction methods for extracting functional networks from large-scale CRISPR screens

Authors

Arshia Zernab Hassan[1*], Henry N. Ward[2*], Mahfuzur Rahman[1], Maximilian Billmann[1,3], Yoonkyu Lee[2], Chad L. Myers[1,2]

*These authors contributed equally to this work

[1] Department of Computer Science and Engineering, University of Minnesota – Twin Cities, Minneapolis, Minnesota, USA

[2] Bioinformatics and Computational Biology Graduate Program, University of Minnesota – Twin Cities, Minneapolis, Minnesota, USA

[3] Institute of Human Genetics, University of Bonn, School of Medicine and University Hospital Bonn, Bonn, Germany

Corresponding author

Chad L. Myers

Email: chadm@umn.edu

Table of contents

Appendix Contents --- Page Number

Appendix Figure S1 --- 1

Appendix Figure S2 --- 3

Appendix Figure S3 --- 5

Appendix Figure S4 --- 7

Appendix Figure S5 --- 9

Appendix Figure S6 --- 11

Appendix Figure S7 --- 13

Appendix Figure S8 --- 15

Appendix Figure S9 --- 17

Appendix Figure S10 -- 19

Appendix Figure S11 -- 21

Appendix Figure S12 -- 23

Appendix Figure S13 -- 25

Appendix Figure S14 -- 27

Appendix Figure S15 -- 29

Appendix Figure S16 -- 31

Appendix Figure S17 -- 33

References --- 35

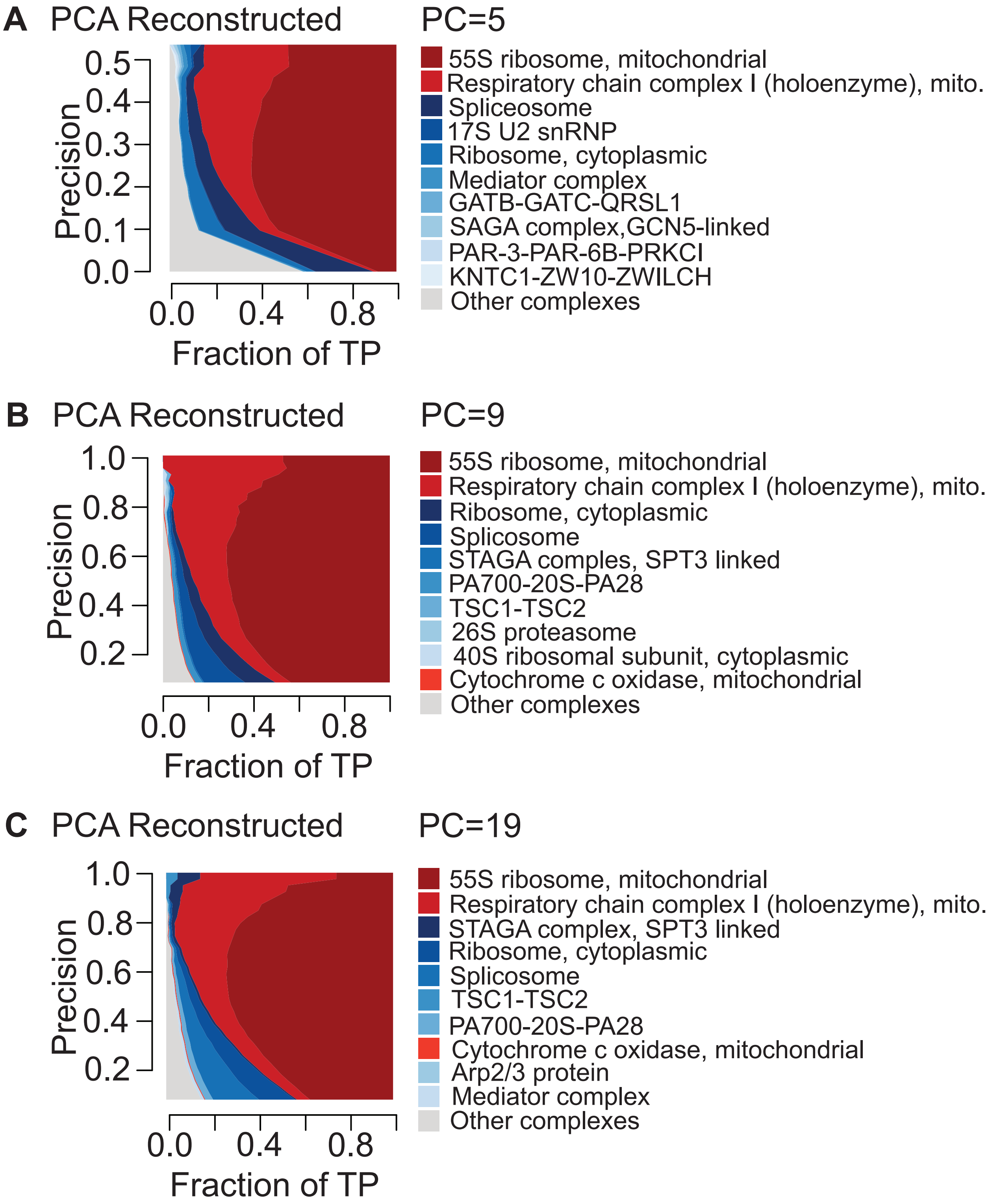
A
PCA Reconstructed
PC=5
Precision
0.5
0.4
0.3
0.2
0.1
0.0
0.0
0.4
0.8
Fraction of TP
55S ribosome, mitochondrial
Respiratory chain complex I (holoenzyme), mito.
Spliceosome
17S U2 snRNP
Ribosome, cytoplasmic
Mediator complex
GATB-GATC-QRSL1
SAGA complex,GCN5-linked
PAR-3-PAR-6B-PRKCI
KNTC1-ZW10-ZWILCH
Other complexes
B
PCA Reconstructed
PC=9
Precision
1.0
0.8
0.6
0.4
0.2
0.0
0.4
0.8
Fraction of TP
55S ribosome, mitochondrial
Respiratory chain complex I (holoenzyme), mito.
Ribosome, cytoplasmic
Splicosome
STAGA comples, SPT3 linked
PA700-20S-PA28
TSC1-TSC2
26S proteasome
40S ribosomal subunit, cytoplasmic
Cytochrome c oxidase, mitochondrial
Other complexes
C
PCA Reconstructed
PC=19
Precision
1.0
0.8
0.6
0.4
0.2
0.0
0.4
0.8
Fraction of TP
55S ribosome, mitochondrial
Respiratory chain complex I (holoenzyme), mito.
STAGA complex, SPT3 linked
Ribosome, cytoplasmic
Splicosome
TSC1-TSC2
PA700-20S-PA28
Cytochrome c oxidase, mitochondrial
Arp2/3 protein
Mediator complex
Other complexes

Appendix Figure S1: Contribution diversity plot depicting true-positive (TP) pairs contributions from CORUM complexes in PCA-reconstructed DepMap 20Q2 (Data ref: Broad DepMap, 2020). The top ten contributing complexes are listed in the legend, with the light gray category representing all complexes represented at lower frequency. Note that figure panel A, B, and C are replicated from Figure 1E.

A, Contribution diversity plot from PCA-reconstructed DepMap data with the first 5 principal components.

B, Contribution diversity plot from PCA-reconstructed DepMap data with the first 9 principal components.

C, Contribution diversity plot from PCA-reconstructed DepMap data with the first 19 principal components.

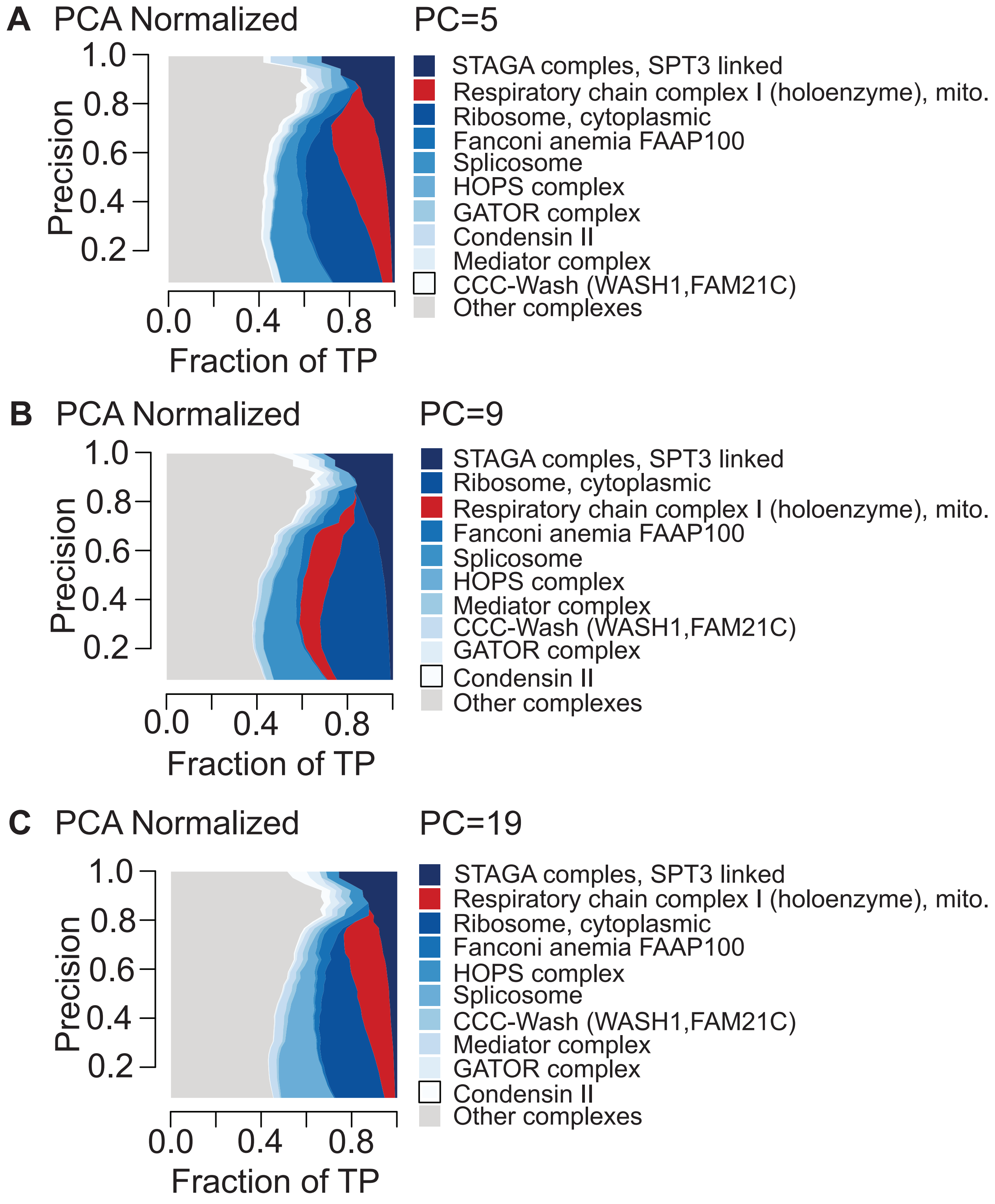
A
PCA Normalized
PC=5
Precision
1.0
0.8
0.6
0.4
0.2
0.0
0.4
0.8
Fraction of TP
STAGA comples, SPT3 linked
Respiratory chain complex I (holoenzyme), mito.
Ribosome, cytoplasmic
Fanconi anemia FAAP100
Splicosome
HOPS complex
GATOR complex
Condensin II
Mediator complex
CCC-Wash (WASH1,FAM21C)
Other complexes
B
PCA Normalized
PC=9
Precision
1.0
0.8
0.6
0.4
0.2
0.0
0.4
0.8
Fraction of TP
STAGA comples, SPT3 linked
Ribosome, cytoplasmic
Respiratory chain complex I (holoenzyme), mito.
Fanconi anemia FAAP100
Splicosome
HOPS complex
Mediator complex
CCC-Wash (WASH1,FAM21C)
GATOR complex
Condensin II
Other complexes
C
PCA Normalized
PC=19
Precision
1.0
0.8
0.6
0.4
0.2
0.0
0.4
0.8
Fraction of TP
STAGA comples, SPT3 linked
Respiratory chain complex I (holoenzyme), mito.
Ribosome, cytoplasmic
Fanconi anemia FAAP100
HOPS complex
Splicosome
CCC-Wash (WASH1,FAM21C)
Mediator complex
GATOR complex
Condensin II
Other complexes

Appendix Figure S2: Contribution diversity plots depicting true-positive (TP) pairs contributions from CORUM complexes in PCA-normalized DepMap 20Q2 data (Data ref: Broad DepMap, 2020). The top ten contributing complexes are listed in the legend, with the light gray category representing all complexes represented at lower frequency. Note that figure panel A, B, and C are replicated from Figure 1E.

A, Contribution diversity plot from PCA-normalized DepMap data with the first 5 principal components removed.

B, Contribution diversity plot from PCA-normalized DepMap data with the first 9 principal components removed.

C, Contribution diversity plot from PCA-normalized DepMap data with the first 19 principal components removed.

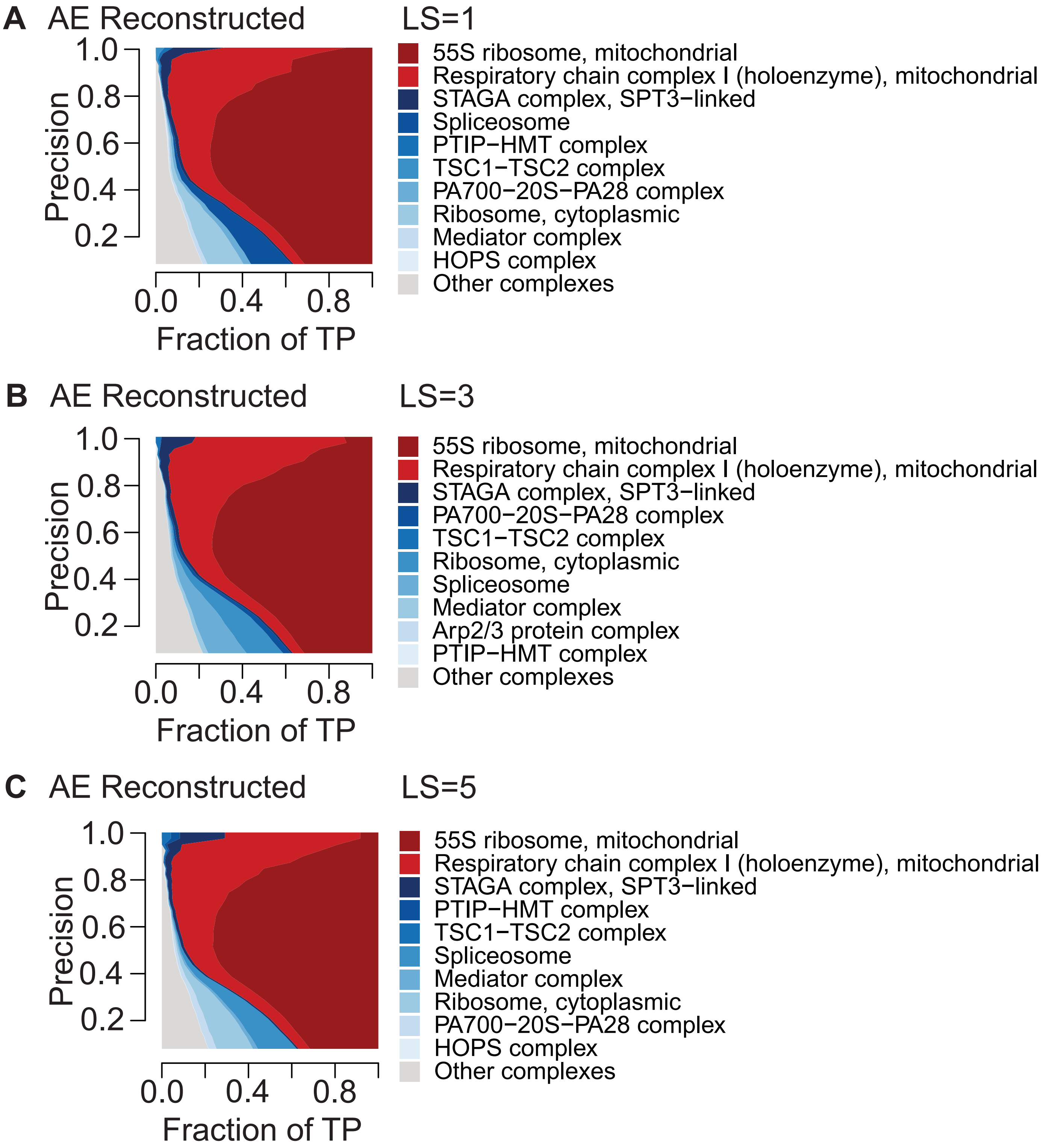
A
AE Reconstructed
LS=1
Precision
1.0
0.8
0.6
0.4
0.2
0.0
0.4
0.8
Fraction of TP
55S ribosome, mitochondrial
Respiratory chain complex I (holoenzyme), mitochondrial
STAGA complex, SPT3−linked
Spliceosome
PTIP−HMT complex
TSC1−TSC2 complex
PA700−20S−PA28 complex
Ribosome, cytoplasmic
Mediator complex
HOPS complex
Other complexes
B
AE Reconstructed
LS=3
Precision
1.0
0.8
0.6
0.4
0.2
0.0
0.4
0.8
Fraction of TP
55S ribosome, mitochondrial
Respiratory chain complex I (holoenzyme), mitochondrial
STAGA complex, SPT3−linked
PA700−20S−PA28 complex
TSC1−TSC2 complex
Ribosome, cytoplasmic
Spliceosome
Mediator complex
Arp2/3 protein complex
PTIP−HMT complex
Other complexes
C
AE Reconstructed
LS=5
Precision
1.0
0.8
0.6
0.4
0.2
0.0
0.4
0.8
Fraction of TP
55S ribosome, mitochondrial
Respiratory chain complex I (holoenzyme), mitochondrial
STAGA complex, SPT3−linked
PTIP−HMT complex
TSC1−TSC2 complex
Spliceosome
Mediator complex
Ribosome, cytoplasmic
PA700−20S−PA28 complex
HOPS complex
Other complexes

Appendix Figure S3: Contribution diversity plots depicting true-positive (TP) pairs contributions from CORUM complex in AE-reconstructed DepMap 20Q2 data generated with latent space sizes 1, 3 and 5 (Data ref: Broad DepMap, 2020). The top ten contributing complexes are listed in the legend, with the light gray category representing all complexes represented at lower frequency. Note that figure panel A, B, and C are replicated from Figure 2A (right panel).

A, Contribution diversity plot from AE-reconstructed DepMap data generated with latent space size 1.

B, Contribution diversity plot from AE-reconstructed DepMap data generated with latent space size 3.

C, Contribution diversity plot from AE-reconstructed DepMap data generated with latent space size 5.

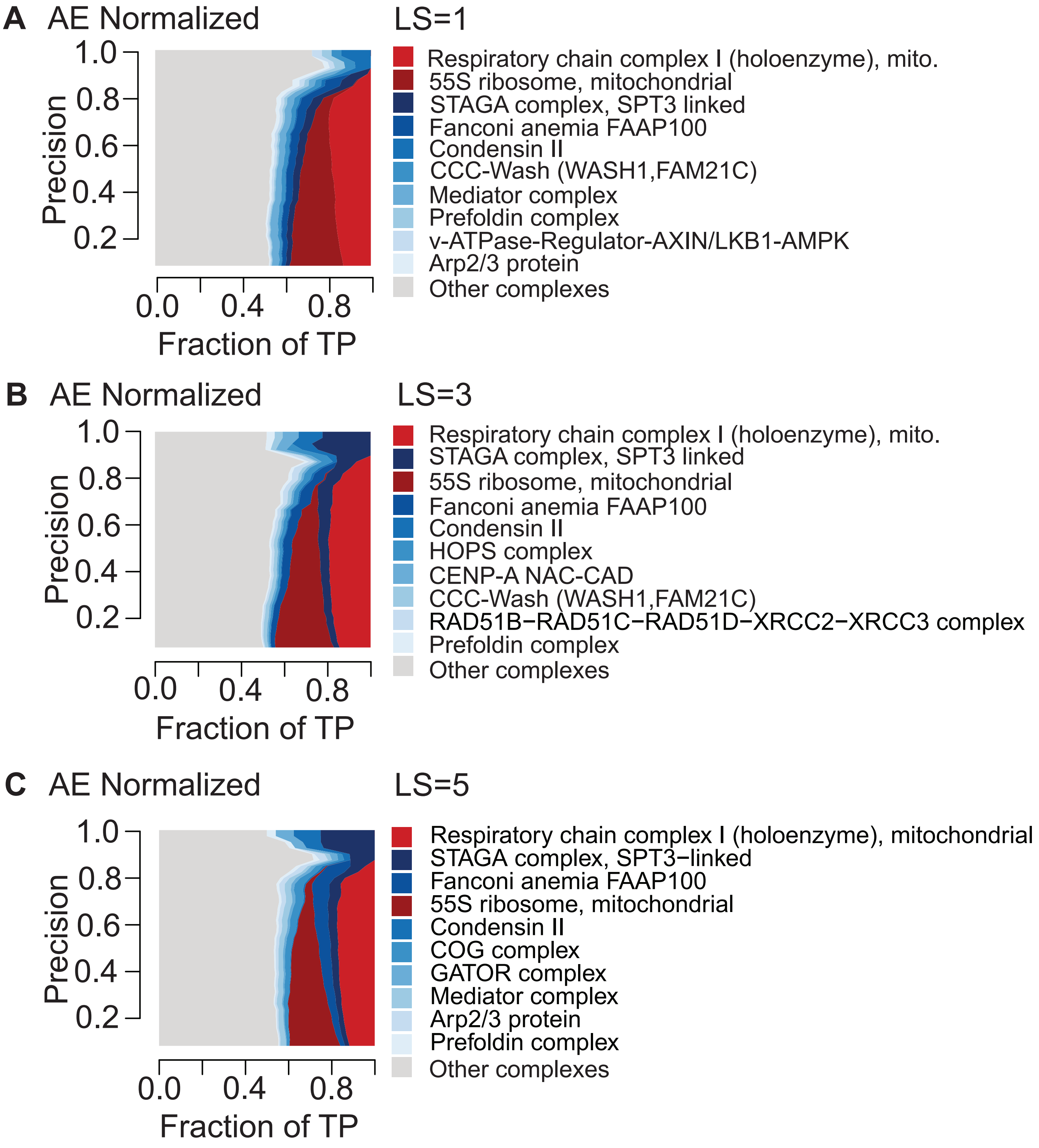
A AE Normalized
LS=1
Precision
1.0
0.8
0.6
0.4
0.2
0.0
0.4
0.8
Fraction of TP
Respiratory chain complex I (holoenzyme), mito.
55S ribosome, mitochondrial
STAGA complex, SPT3 linked
Fanconi anemia FAAP100
Condensin II
CCC-Wash (WASH1,FAM21C)
Mediator complex
Prefoldin complex
v-ATPase-Regulator-AXIN/LKB1-AMPK
Arp2/3 protein
Other complexes
B AE Normalized
LS=3
Respiratory chain complex I (holoenzyme), mito.
STAGA complex, SPT3 linked
55S ribosome, mitochondrial
Fanconi anemia FAAP100
Condensin II
HOPS complex
CENP-A NAC-CAD
CCC-Wash (WASH1,FAM21C)
RAD51B−RAD51C−RAD51D−XRCC2−XRCC3 complex
Prefoldin complex
Other complexes
C AE Normalized
LS=5
Respiratory chain complex I (holoenzyme), mitochondrial
STAGA complex, SPT3−linked
Fanconi anemia FAAP100
55S ribosome, mitochondrial
Condensin II
COG complex
GATOR complex
Mediator complex
Arp2/3 protein
Prefoldin complex
Other complexes

Appendix Figure S4: Contribution diversity plots depicting true-positive (TP) pairs contributions from CORUM complex in AE-normalized DepMap 20Q2 data (Data ref: Broad DepMap, 2020). The top ten contributing complexes are listed in the legend, with the light gray category representing all complexes represented at lower frequency. Note that figure panel A, B, and C are replicated from Figure 2A (right panel).

A, Contribution diversity plot from AE-normalized DepMap data generated with latent space size 1.

B, Contribution diversity plot from AE-normalized DepMap data generated with latent space size 3.

C, Contribution diversity plot from AE-normalized DepMap data generated with latent space size 5.

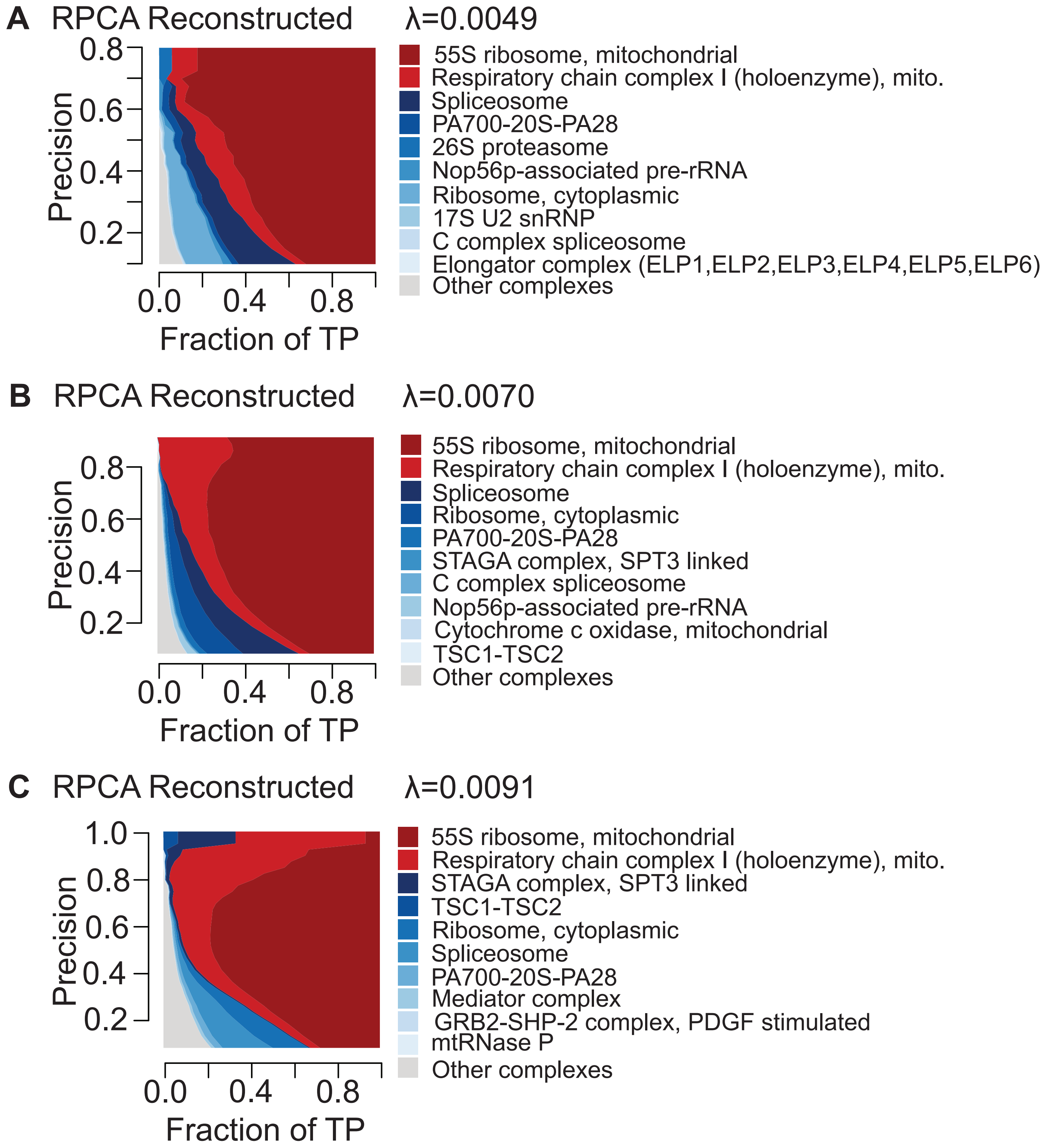
A
RPCA Reconstructed
λ=0.0049
Precision
0.8
0.6
0.4
0.2
0.0
0.4
0.8
Fraction of TP
55S ribosome, mitochondrial
Respiratory chain complex I (holoenzyme), mito.
Spliceosome
PA700-20S-PA28
26S proteasome
Nop56p-associated pre-rRNA
Ribosome, cytoplasmic
17S U2 snRNP
C complex spliceosome
Elongator complex (ELP1,ELP2,ELP3,ELP4,ELP5,ELP6)
Other complexes
B
RPCA Reconstructed
λ=0.0070
Precision
0.8
0.6
0.4
0.2
0.0
0.4
0.8
Fraction of TP
55S ribosome, mitochondrial
Respiratory chain complex I (holoenzyme), mito.
Spliceosome
Ribosome, cytoplasmic
PA700-20S-PA28
STAGA complex, SPT3 linked
C complex spliceosome
Nop56p-associated pre-rRNA
Cytochrome c oxidase, mitochondrial
TSC1-TSC2
Other complexes
C
RPCA Reconstructed
λ=0.0091
Precision
1.0
0.8
0.6
0.4
0.2
0.0
0.4
0.8
Fraction of TP
55S ribosome, mitochondrial
Respiratory chain complex I (holoenzyme), mito.
STAGA complex, SPT3 linked
TSC1-TSC2
Ribosome, cytoplasmic
Spliceosome
PA700-20S-PA28
Mediator complex
GRB2-SHP-2 complex, PDGF stimulated
mtRNase P
Other complexes

Appendix Figure S5: Contribution diversity plots illustrating true-positive (TP) pairs contributions from CORUM complexes in RPCA-reconstructed DepMap 20Q2 data (Data ref: Broad DepMap, 2020). The top ten contributing complexes are listed in the legend, with the light gray category representing all complexes represented at lower frequency. Note that figure panel A, B, and C are replicated from Figure 2B (right panel).
A, Contribution diversity plot from RPCA-reconstructed DepMap data generated with hyperparameter λ set to 0.0049.
B, Contribution diversity plot from RPCA-reconstructed DepMap data generated with hyperparameter λ set to 0.007.
C, Contribution diversity plot from RPCA-reconstructed DepMap data generated with hyperparameter λ set to 0.0091.

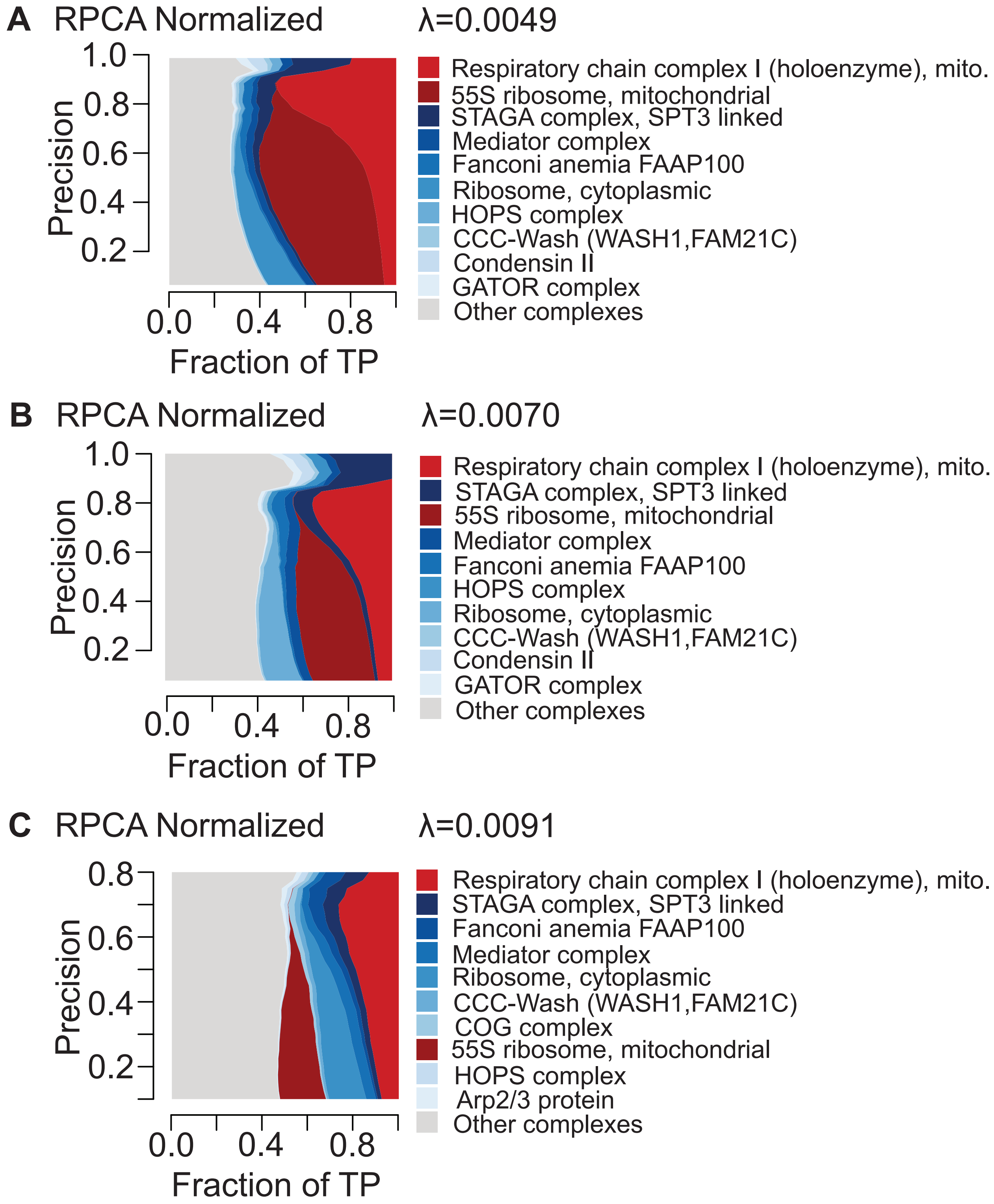
A RPCA Normalized
λ=0.0049
Precision
1.0
0.8
0.6
0.4
0.2
0.0
0.4
0.8
Fraction of TP
Respiratory chain complex I (holoenzyme), mito.
55S ribosome, mitochondrial
STAGA complex, SPT3 linked
Mediator complex
Fanconi anemia FAAP100
Ribosome, cytoplasmic
HOPS complex
CCC-Wash (WASH1,FAM21C)
Condensin II
GATOR complex
Other complexes
B RPCA Normalized
λ=0.0070
Precision
1.0
0.8
0.6
0.4
0.2
0.0
0.4
0.8
Fraction of TP
Respiratory chain complex I (holoenzyme), mito.
STAGA complex, SPT3 linked
55S ribosome, mitochondrial
Mediator complex
Fanconi anemia FAAP100
HOPS complex
Ribosome, cytoplasmic
CCC-Wash (WASH1,FAM21C)
Condensin II
GATOR complex
Other complexes
C RPCA Normalized
λ=0.0091
Precision
0.8
0.6
0.4
0.2
0.0
0.4
0.8
Fraction of TP
Respiratory chain complex I (holoenzyme), mito.
STAGA complex, SPT3 linked
Fanconi anemia FAAP100
Mediator complex
Ribosome, cytoplasmic
CCC-Wash (WASH1,FAM21C)
COG complex
55S ribosome, mitochondrial
HOPS complex
Arp2/3 protein
Other complexes

Appendix Figure S6: Contribution diversity plots illustrating true-positive (TP) pairs contributions from CORUM complexes in RPCA-normalized DepMap 20Q2 (Data ref: Broad DepMap, 2020). The top ten contributing complexes are listed in the legend, with the light gray category representing all complexes represented at lower frequency. Note that figure panel A, B, and C are replicated from Figure 2B (right panel).

A, Contribution diversity plot from RPCA-normalized DepMap data generated with hyperparameter λ set to 0.0049.

B, Contribution diversity plot from RPCA-normalized DepMap data generated with hyperparameter λ set to 0.007.

C, Contribution diversity plot from RPCA-normalized DepMap data generated with hyperparameter λ set to 0.0091.

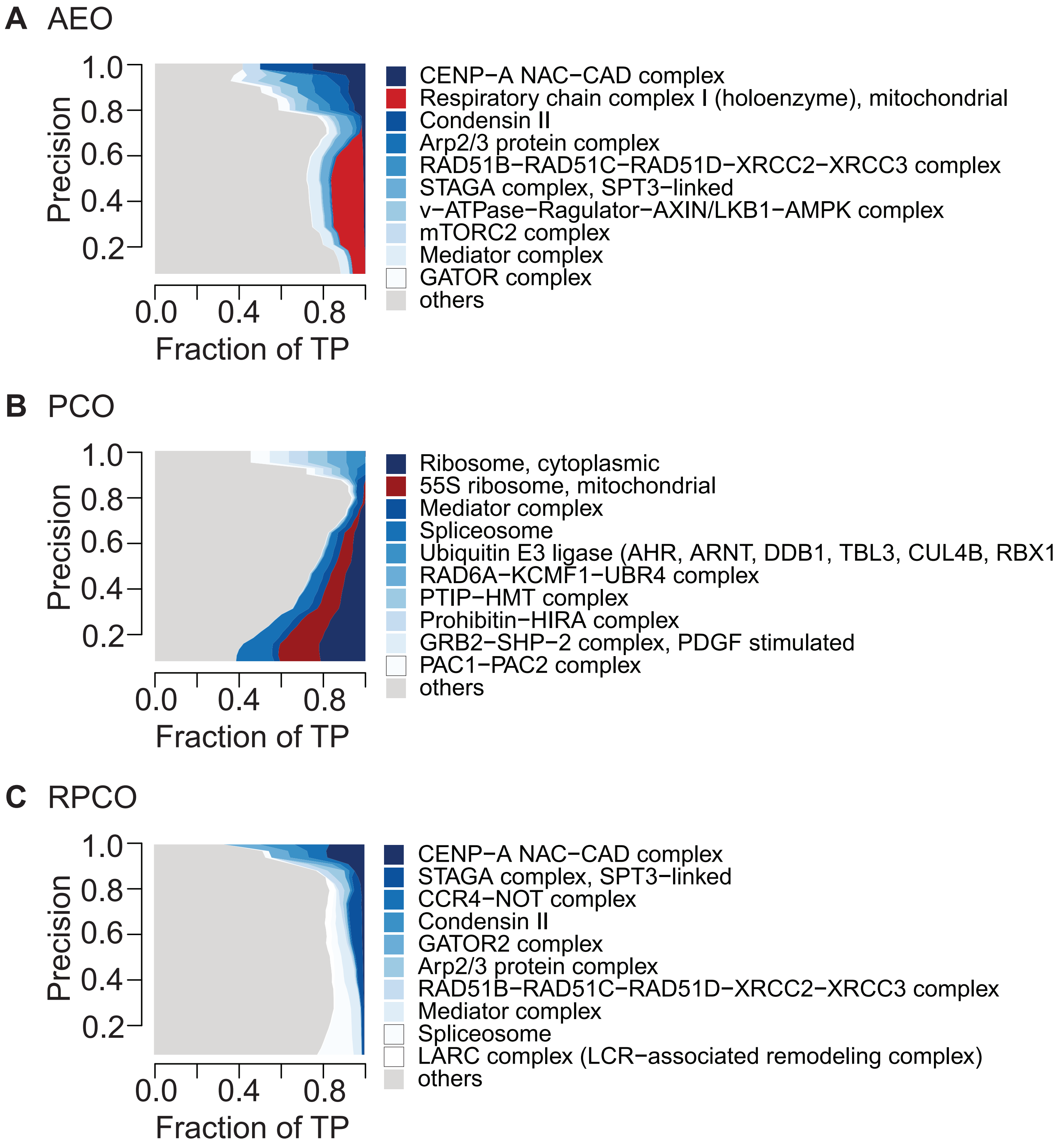
A AEO
1.0
0.8
0.6
0.4
0.2
Precision
0.0
0.4
0.8
Fraction of TP
CENP−A NAC−CAD complex
Respiratory chain complex I (holoenzyme), mitochondrial
Condensin II
Arp2/3 protein complex
RAD51B−RAD51C−RAD51D−XRCC2−XRCC3 complex
STAGA complex, SPT3−linked
v−ATPase−Ragulator−AXIN/LKB1−AMPK complex
mTORC2 complex
Mediator complex
GATOR complex
others
B PCO
1.0
0.8
0.6
0.4
0.2
Precision
0.0
0.4
0.8
Fraction of TP
Ribosome, cytoplasmic
55S ribosome, mitochondrial
Mediator complex
Spliceosome
Ubiquitin E3 ligase (AHR, ARNT, DDB1, TBL3, CUL4B, RBX1
RAD6A−KCMF1−UBR4 complex
PTIP−HMT complex
Prohibitin−HIRA complex
GRB2−SHP−2 complex, PDGF stimulated
PAC1−PAC2 complex
others
C RPCO
1.0
0.8
0.6
0.4
0.2
Precision
0.0
0.4
0.8
Fraction of TP
CENP−A NAC−CAD complex
STAGA complex, SPT3−linked
CCR4−NOT complex
Condensin II
GATOR2 complex
Arp2/3 protein complex
RAD51B−RAD51C−RAD51D−XRCC2−XRCC3 complex
Mediator complex
Spliceosome
LARC complex (LCR−associated remodeling complex)
others

Appendix Figure S7: Contribution diversity plots depicting true-positive (TP) pairs contributions from CORUM complexes in onion normalized DepMap 20Q2 data (Data ref: Broad DepMap, 2020). The top ten contributing complexes are listed in the legend, with the light gray category representing all complexes represented at lower frequency. Note that figure panel A and B are replicated from Figure 3C. Figure panel C is replicated from Figure 3C and 4B.

A, Contribution diversity plot from onion normalized DepMap data generated with SNF (Wang *et al*, 2014) integrated AE-normalized layers (AEO).

B, Contribution diversity plot from onion normalized DepMap data generated with SNF integrated PCA-normalized layers (PCO).

C, Contribution diversity plot from onion normalized DepMap data generated with SNF integrated RPCA-normalized layers (RPCO).

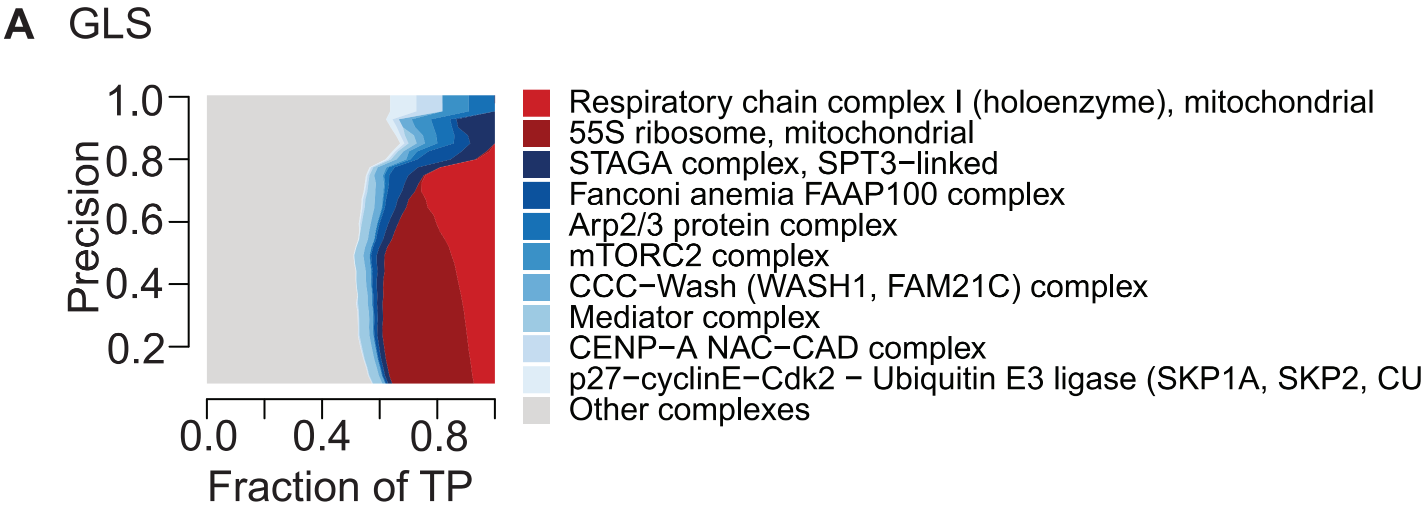
A
GLS
Precision
1.0
0.8
0.6
0.4
0.2
0.0
0.4
0.8
Fraction of TP
Respiratory chain complex I (holoenzyme), mitochondrial
55S ribosome, mitochondrial
STAGA complex, SPT3−linked
Fanconi anemia FAAP100 complex
Arp2/3 protein complex
mTORC2 complex
CCC−Wash (WASH1, FAM21C) complex
Mediator complex
CENP−A NAC−CAD complex
p27−cyclinE−Cdk2 − Ubiquitin E3 ligase (SKP1A, SKP2, CU
Other complexes

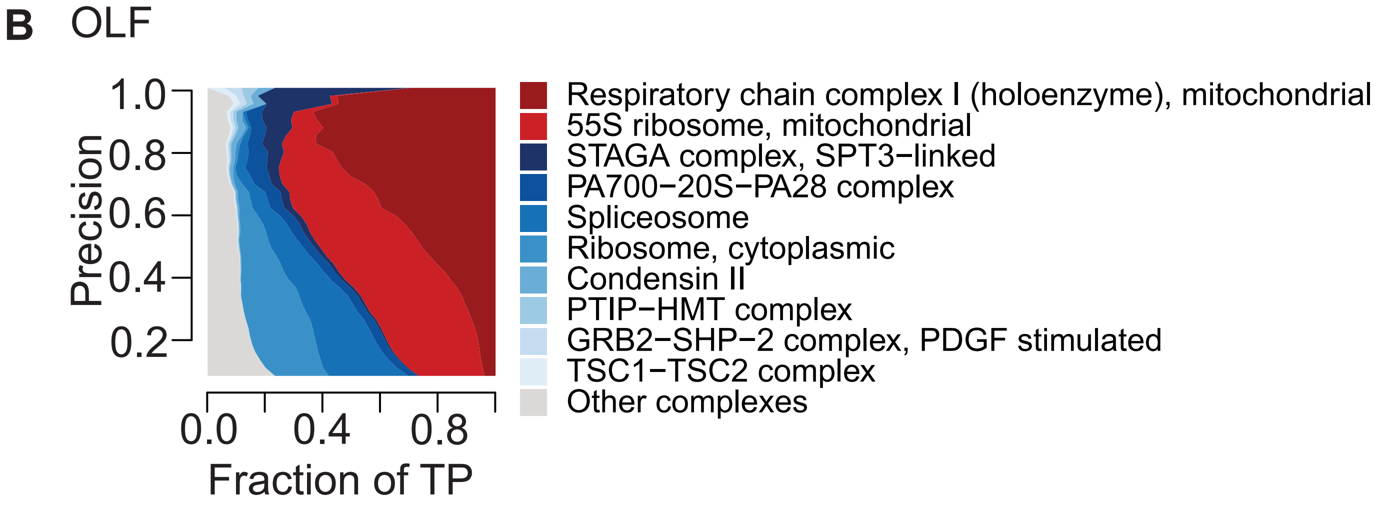
B
OLF
Precision
1.0
0.8
0.6
0.4
0.2
0.0
0.4
0.8
Fraction of TP
Respiratory chain complex I (holoenzyme), mitochondrial
55S ribosome, mitochondrial
STAGA complex, SPT3−linked
PA700−20S−PA28 complex
Spliceosome
Ribosome, cytoplasmic
Condensin II
PTIP−HMT complex
GRB2−SHP−2 complex, PDGF stimulated
TSC1−TSC2 complex
Other complexes

Appendix Figure S8: Contribution diversity plots depicting TP pairs contributions from CORUM complexes in generalized least squares (GLS) normalized (Wainberg *et al*, 2021), and olfactory receptor (OLF) normalized (Boyle *et al*, 2018) DepMap 20Q2 data (Data ref: Broad DepMap, 2020). The top ten contributing complexes are listed in the legend, with the light gray category representing all complexes represented at lower frequency. Note that figure panel A and B are replicated from Figure 4B.

A, Contribution diversity plot from GLS normalized DepMap data.

B, Contribution diversity plot from OLF normalized DepMap data.

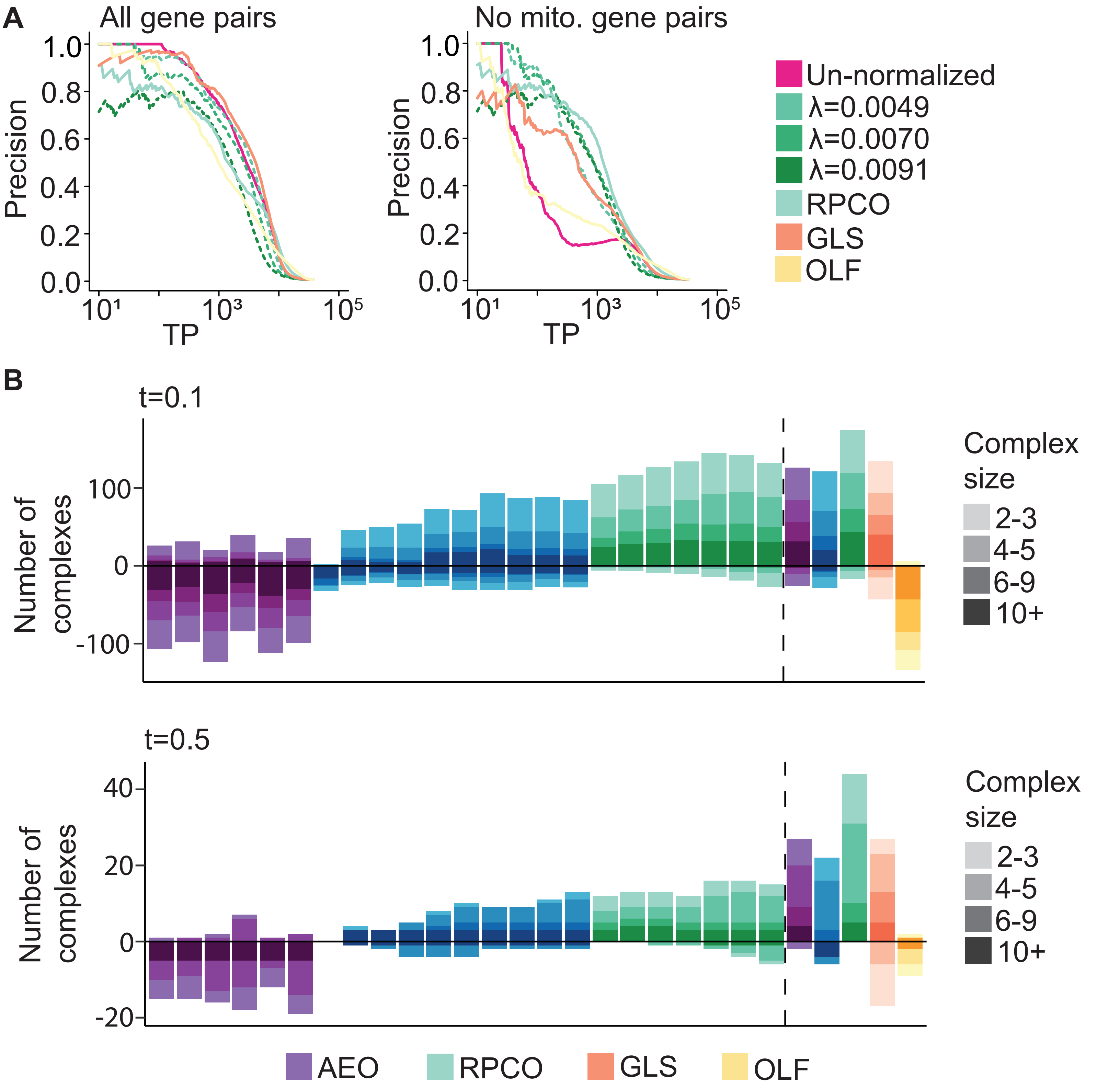

A
All gene pairs
No mito. gene pairs
Precision
1.0
0.8
0.6
0.4
0.2
0.0
10^1
10^3
10^5
TP
Un-normalized
λ=0.0049
λ=0.0070
λ=0.0091
RPCO
GLS
OLF
B
t=0.1
Number of complexes
100
0
-100
Complex size
2-3
4-5
6-9
10+
t=0.5
40
20
0
-20
AEO
RPCO
GLS
OLF

Appendix Figure S9: The performance comparison of individual normalized layer networks with the networks from Onion-normalization and other methods applied to DepMap 20Q2 (Data ref: Broad DepMap, 2020).

A, FLEX precision-recall (PR) performance analysis of networks from original DepMap, robust PCA (RPCA) normalization with hyperparameter λ set to 0.0049, 0.007 and 0.0091, onion normalization with robust PCA (RPCO), generalized least squares (GLS) normalization (Wainberg *et al*, 2021), and olfactory receptor (OLF) normalization (Boyle *et al*, 2018) with CORUM protein complexes as the standard. (Left) All CORUM complex gene pairs as true-positive. (Right) Mitochondrial gene pairs are removed from the evaluation. The x-axis of both plots depicts the absolute number of true positives (TPs) recovered in log scale.

B, Number of complexes for which area under the PR curve (AUPRC) values increase and decrease with respect to chosen AUPRC thresholds due to normalization as compared to un-normalized data. The bars on the left side of the dotted line correspond to AE-normalized layers (latent space size = 1, 2, 3, 4, 5, 10), PCA-normalized layers (first 1, 3, 5, 7, 9, 11, 13, 15, 17, 19 principal components removed) and RPCA layers ($\tilde{\lambda}$= 0.0049, 0.0056, 0.0063, 0.007, 0.0077, 0.0084, 0.0091). The bars on the right side of the dotted line correspond to Onion-normalized data from the PCA, PRCA, and AE layers as well as GLS and OLF. The color gradient for each method represents four bins with complexes containing 2 to 3 genes, 4 to 5 genes, 6 to 9 genes, and 10 or more genes. (Top) AUPRC threshold, t = 0.1. (Bottom) t = 0.5.

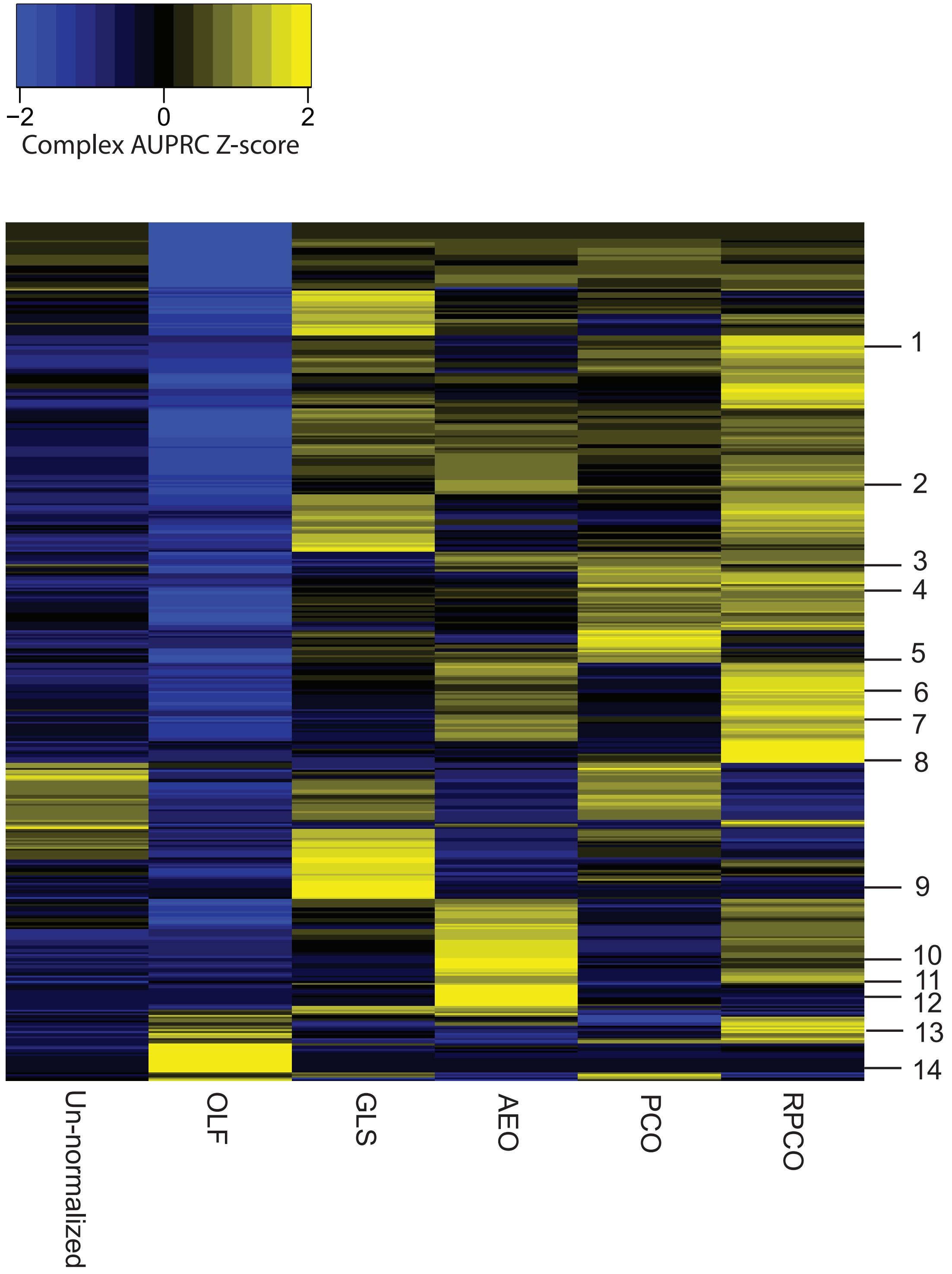

1. KRIT1-CCM2-ICAP1 complex
2. EBAFb complex
3. CDK8 subcomplex
4. GINS complex
5. FA complex
6. SAP complex
7. BRCA1 C complex
8. TBCD-ARL2-tubulin(beta)-TBCE complex
9. NoRC complex
10. AP1B1-AP1G2-AP1M-1AP1S1 complex
11. NELF complex
12. ITGAV-ITGB3-SLC3A2 complex
13. ANCO1-HDAC3 complex
14. CCND3-CDK6 complex

Appendix Figure S10: CORUM complex z-scores of AUPRC values for onion normalization. Z-scores across rows for un-normalized DepMap 20Q2 data (Data ref: Broad DepMap, 2020) compared to data from generalized least squares (GLS) normalization from Wainberg et al. (Wainberg *et al*, 2021), olfactory receptor (OLF) normalization from Boyle et al. (Boyle *et al*, 2018), onion normalization with AE (AEO), onion normalization with PCA (PCO), and onion normalization with robust PCA (RPCO).

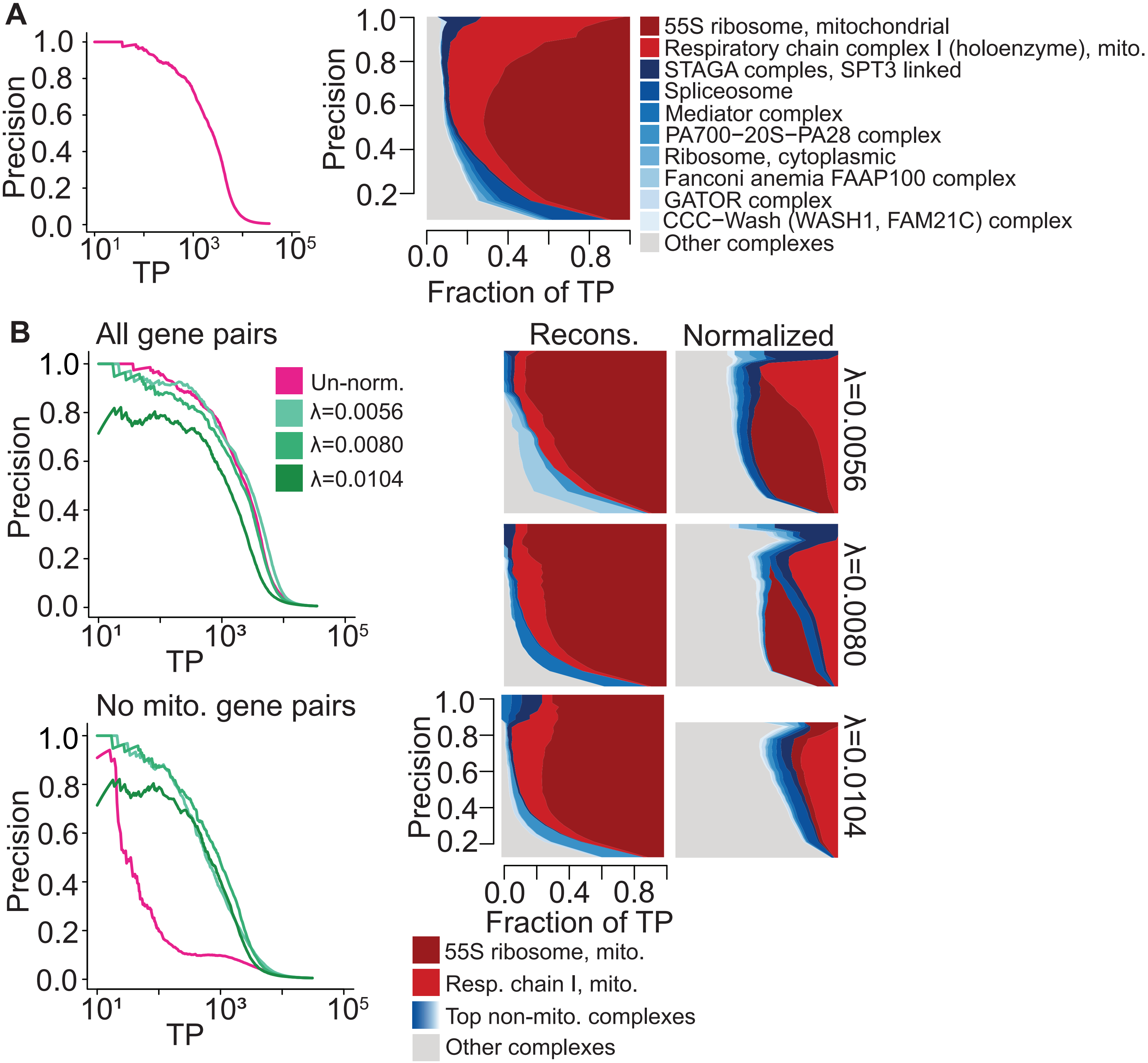
A
Precision
TP
Fraction of TP
55S ribosome, mitochondrial
Respiratory chain complex I (holoenzyme), mito.
STAGA complex, SPT3 linked
Spliceosome
Mediator complex
PA700−20S−PA28 complex
Ribosome, cytoplasmic
Fanconi anemia FAAP100 complex
GATOR complex
CCC−Wash (WASH1, FAM21C) complex
Other complexes
B
All gene pairs
Un-norm.
λ=0.0056
λ=0.0080
λ=0.0104
No mito. gene pairs
Recons.
Normalized
λ=0.0056
λ=0.0080
λ=0.0104
55S ribosome, mito.
Resp. chain I, mito.
Top non-mito. complexes
Other complexes

Appendix Figure S11: Exploration of mitochondrial bias within the DepMap 22Q4 Chronos (Data ref: Broad DepMap, 2022) and RPCA normalization across hyperparameters.
A, (Left) Precision- recall (PR) performance analysis of un-normalized DepMap 22Q4 Chronos gene similarity network evaluated against CORUM protein complex standard. The x-axis depicts the number of true positives (TPs) in log-scale. (Right) Contribution diversity plot of CORUM complexes in un-normalized DepMap data. This plot is constructed by sliding a precision cutoff from high to low (indicated by the y-axis), and at each point, plotting a stacked bar plot across the x-axis at that point reflecting the breakdown of complex membership of the TP pairs identified at that threshold. The top ten contributing complexes are listed in the legend, with the light gray category representing all complexes represented at lower frequency.
B, (Left) PR performance analysis of RPCA-normalized data generated with hyperparameter λ set to 0.0049, 0.007 and 0.0091 evaluated against CORUM protein complex as standard. (Right) Corresponding contribution diversity plots illustrating complex contributions in RPCA-reconstructed and RPCA-normalized data. The x-axis depicts the absolute number of true positives (TPs) recovered in log scale.

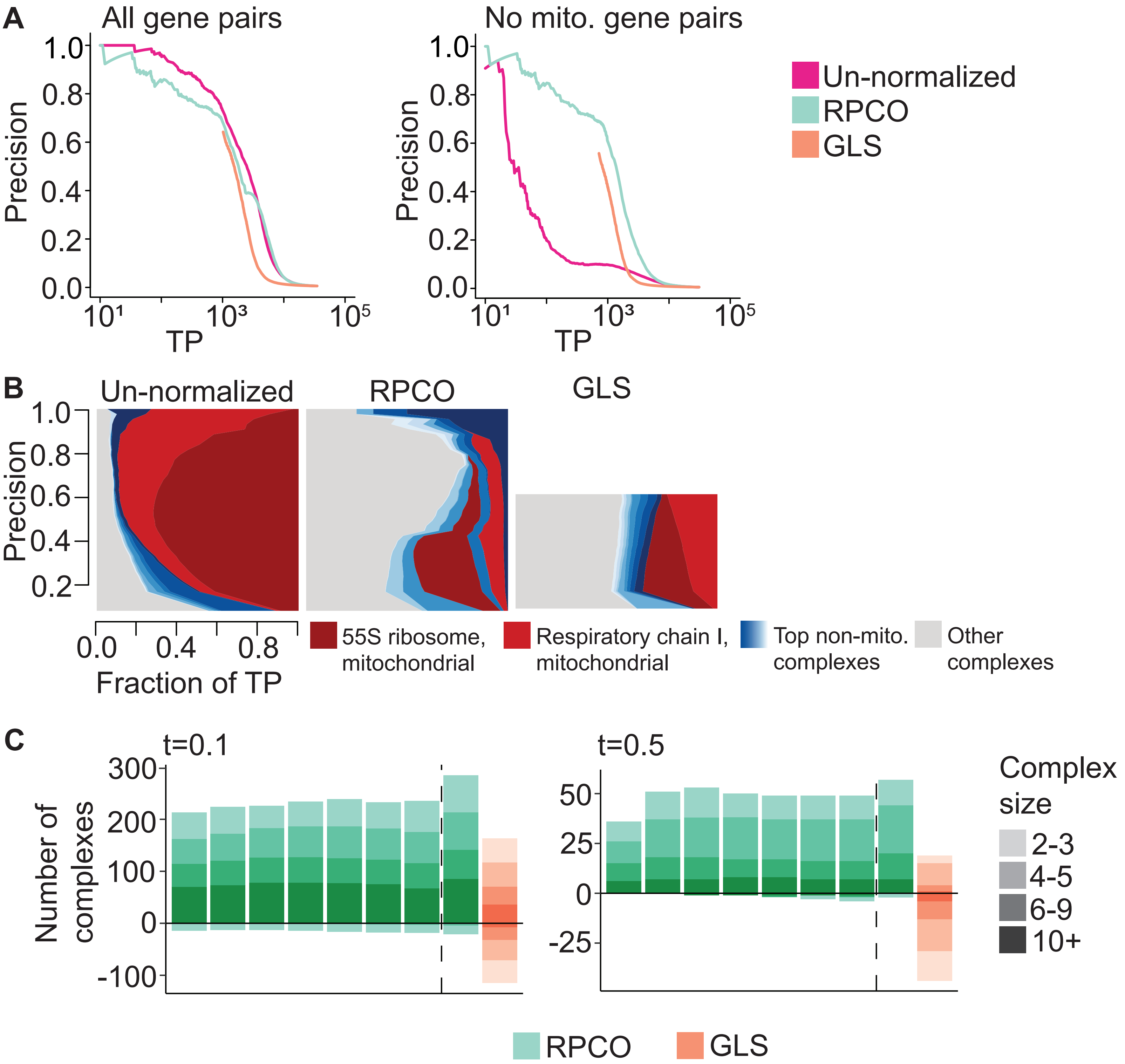
A
All gene pairs
No mito. gene pairs
Precision
TP
Un-normalized
RPCO
GLS
B
Un-normalized
RPCO
GLS
Precision
Fraction of TP
55S ribosome, mitochondrial
Respiratory chain I, mitochondrial
Top non-mito. complexes
Other complexes
C
t=0.1
t=0.5
Number of complexes
Complex size
2-3
4-5
6-9
10+
RPCO
GLS

Appendix Figure S12: Onion-normalization applied to DepMap 22Q4 Chronos data (Data ref: Broad DepMap, 2022) with RPCA as the normalization technique.
A, FLEX precision-recall (PR) performance analysis of original DepMap 22Q4 Chronos data, onion normalization with robust PCA (RPCO), and generalized least squares (GLS) normalization (Wainberg *et al*, 2021) evaluated against CORUM protein complex standard. The x-axis depicts the absolute number of true positives (TPs) recovered in log scale.
B, Contribution diversity of CORUM complexes for the original DepMap, RPCO, and GLS. Fractions of predicted true positives (TP) from different complexes are plotted at various precision levels on the y-axis. Note that the left panel is replicated from Appendix Figure S11A (right panel).
C, Number of complexes for which area under the PR curve (AUPRC) values increase and decrease with respect to chosen AUPRC thresholds due to normalization as compared to un-normalized data. The bars on the left side of the dotted line correspond to RPCA-normalized layers ($\tilde{\lambda}$= 0.0056, 0.0064, 0.0072, 0.0080, 0.0088, 0.0096, 0.0104). The bars on the right side of the dotted line correspond to the RPCO-normalized data from the layers as well as GLS data. The color gradient for each method represents four bins with complexes containing 2 to 3 genes, 4 to 5 genes, 6 to 9 genes, and 10 or more genes. (Left) AUPRC threshold, t = 0.1. (Right) t = 0.5.

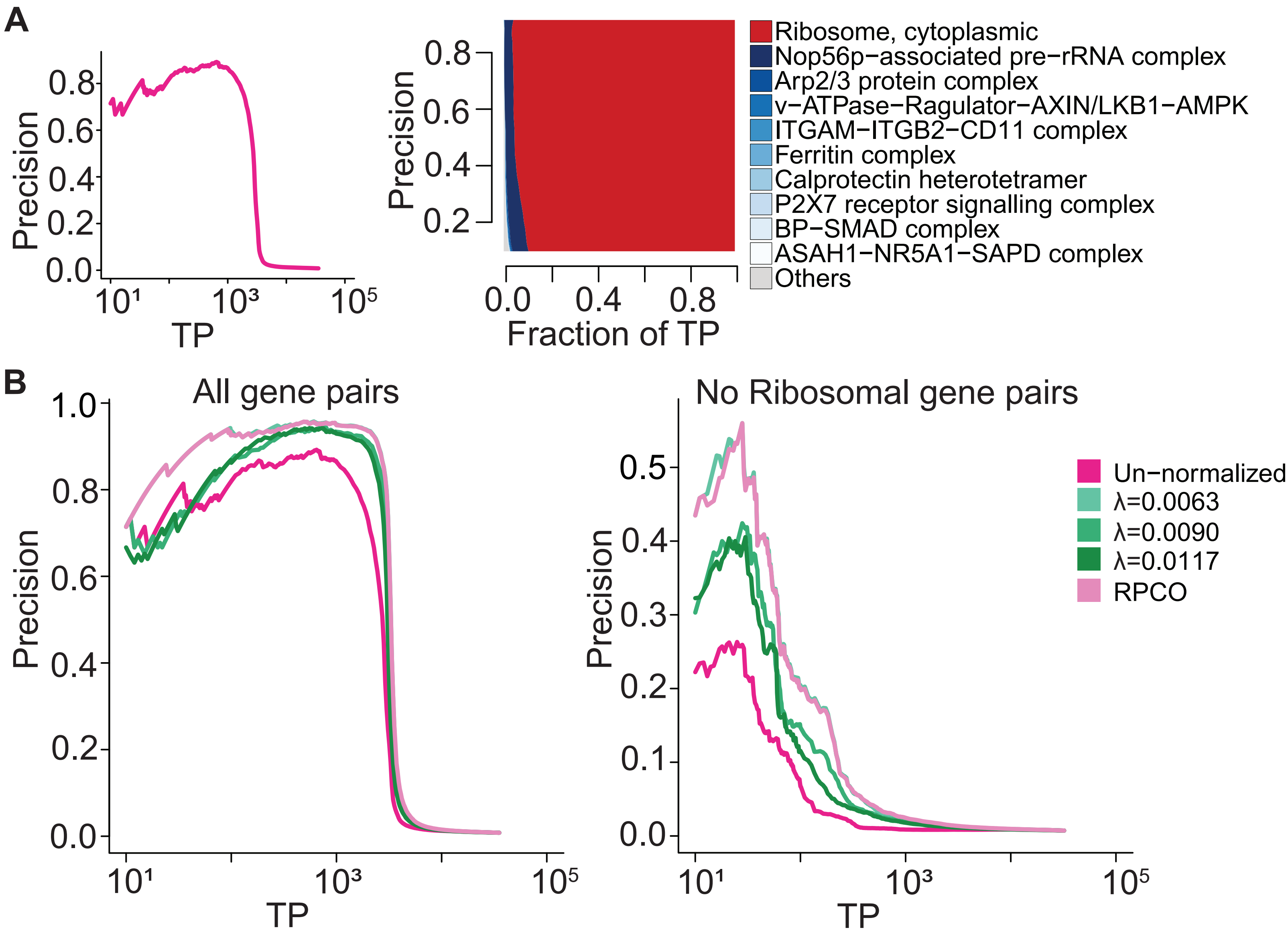
A
Precision
TP
Fraction of TP
Ribosome, cytoplasmic
Nop56p−associated pre−rRNA complex
Arp2/3 protein complex
v−ATPase−Ragulator−AXIN/LKB1−AMPK
ITGAM−ITGB2−CD11 complex
Ferritin complex
Calprotectin heterotetramer
P2X7 receptor signalling complex
BP−SMAD complex
ASAH1−NR5A1−SAPD complex
Others
B
All gene pairs
No Ribosomal gene pairs
Un−normalized
λ=0.0063
λ=0.0090
λ=0.0117
RPCO

Appendix Figure S13: Onion-normalization applied to RNA-seq gene expression data (Data ref: 10x Genomics, 2019) with RPCA as the normalization technique and maximum-weight as integration method.

A, (Left) Precision-recall (PR) performance analysis of un-normalized gene expression data evaluated against CORUM protein complexes. The x-axis depicts the number of true positives (TPs) in log scale. (Right) Contribution diversity plot illustrating TP pairs contributions from CORUM complexes in un-normalized gene expression data. The x-axis is the fraction of gene pairs correctly predicted as functionally related at different precision levels in the y-axis. The legend shows the top ten contributing complexes.

B, (Left) PR performance analysis of RPCA-normalized data generated with λ set to 0.0063, 0.009, and 0.0117 as well as RPCO-normalized data evaluated against CORUM protein complexes. (Right) PR performance of RPCA-normalized data generated with λ set to 0.0063, 0.009, and 0.0117 as well as RPCO-normalized data evaluated against CORUM protein complexes excluding cytoplasmic ribosomal gene pairs from the evaluation process. The x-axis depicts the absolute number of true positives (TPs) recovered in log scale.

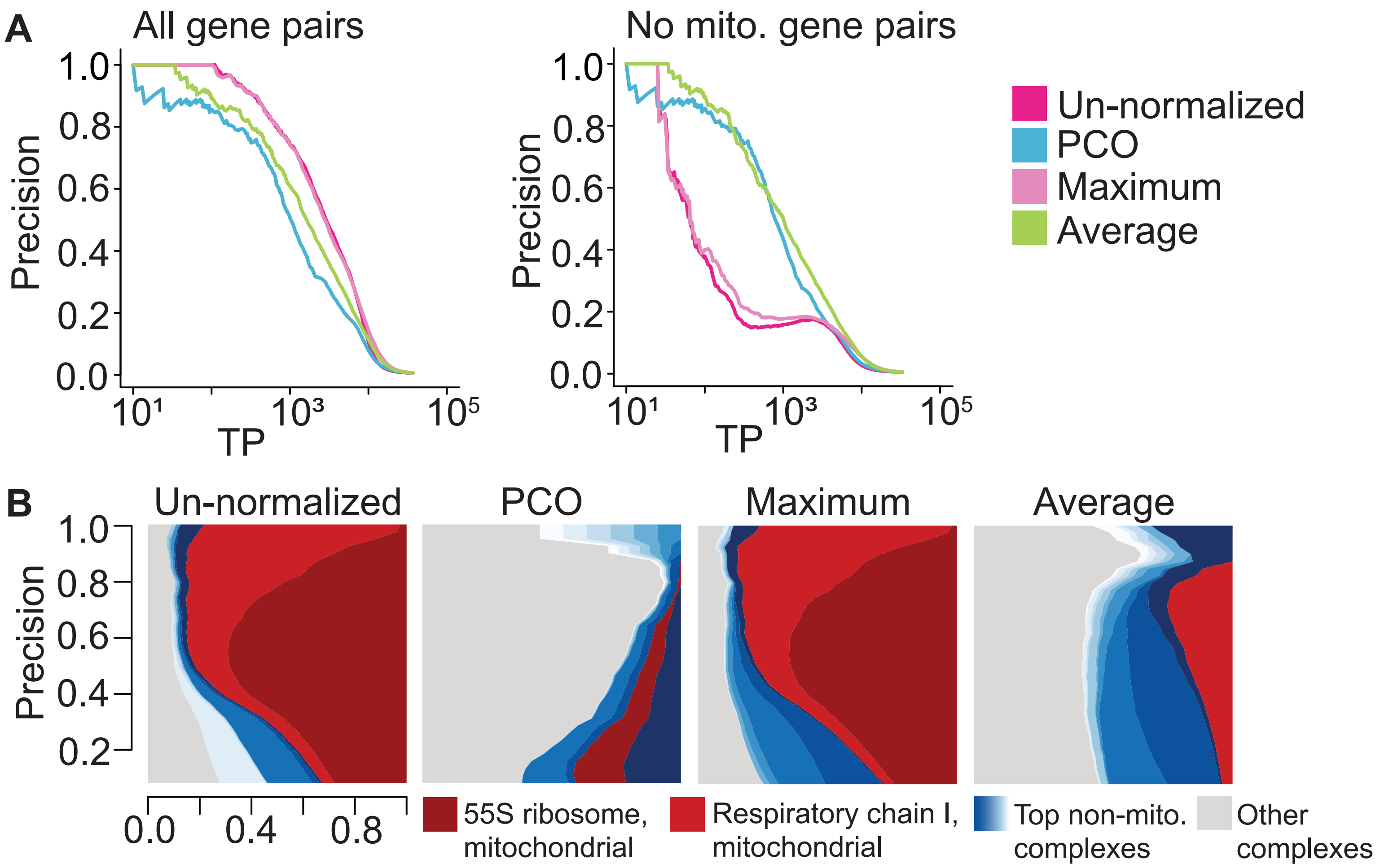

A
All gene pairs
No mito. gene pairs
Precision
1.0
0.8
0.6
0.4
0.2
0.0
10¹
10³
10⁵
TP
Un-normalized
PCO
Maximum
Average
B
Un-normalized
PCO
Maximum
Average
Precision
0.0
0.4
0.8
55S ribosome, mitochondrial
Respiratory chain I, mitochondrial
Top non-mito. complexes
Other complexes

Appendix Figure S14: Onion-normalization with PCA as dimensionality reduction method and maximum or average similarity as integration method applied to DepMap 20Q2 (Data ref: Broad DepMap, 2020).

A, Comparison of Precision-recall (PR) performance of original DepMap data, SNF (Wang *et al*, 2014) integrated PCA-normalized layers (PCO), average of PCA-normalized layers and maximum across PCA-normalized layers evaluated against CORUM complex standard. Layers are generated by removing the first n principal components where n = 1, 3, 5, 7, 9, 11, 13, 15, 17, 19. The x-axis depicts the absolute number of true positives (TPs) recovered in log scale.

B, Contribution diversity plots from original DepMap data, SNF integrated PCA-normalized layers (PCO), average of PCA-normalized layers and maximum across PCA-normalized layers evaluated against CORUM complex standard. The x-axis depicts the absolute number of true positives (TPs) recovered in log scale. Note that the left panel is replicated from Figure 1C (right panel) and the second panel is replicated from Figure 3C.

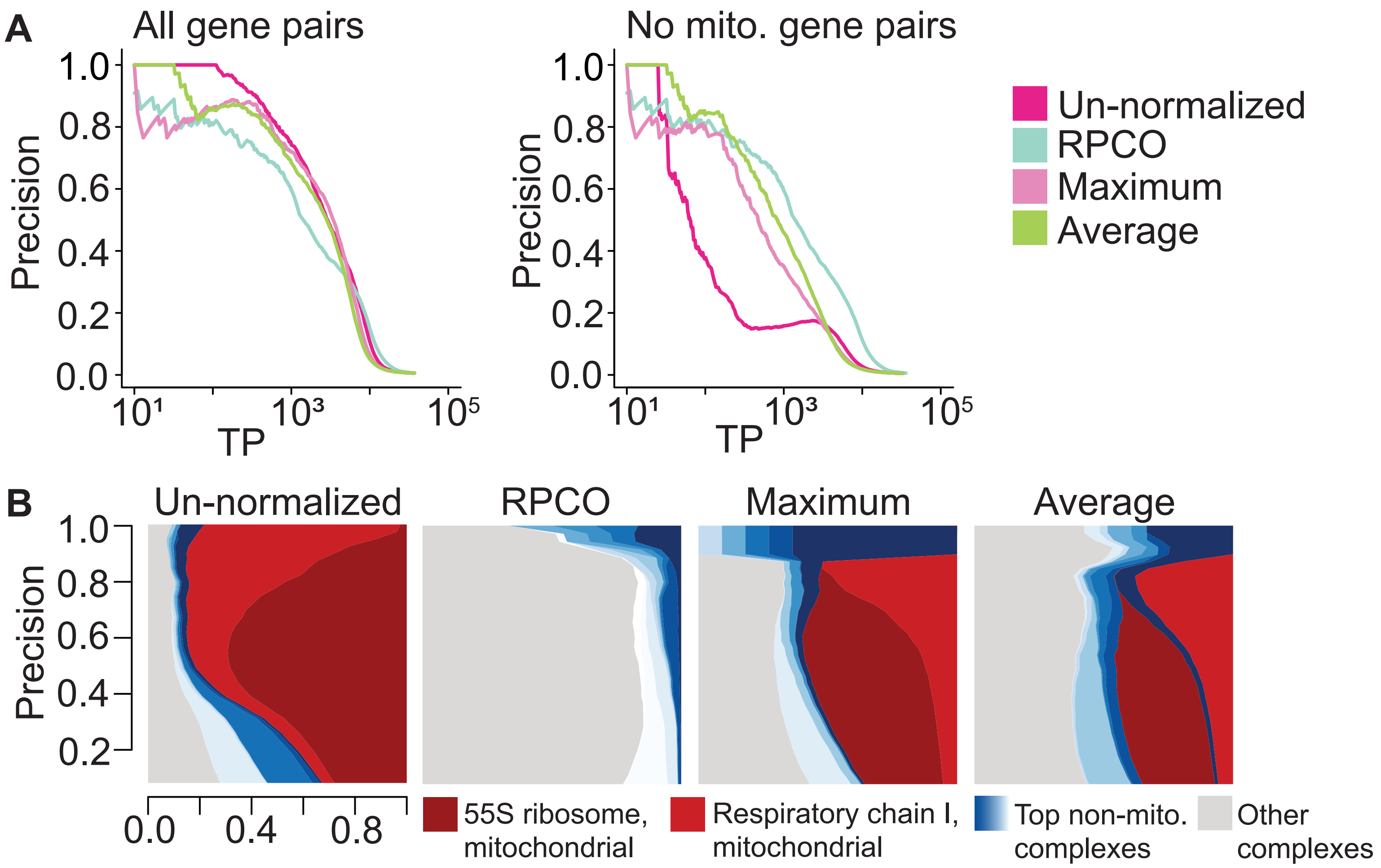

A
All gene pairs
No mito. gene pairs
Precision
1.0
0.8
0.6
0.4
0.2
0.0
10¹
10³
10⁵
TP
Un-normalized
RPCO
Maximum
Average
B
Un-normalized
RPCO
Maximum
Average
Precision
0.0
0.4
0.8
55S ribosome, mitochondrial
Respiratory chain I, mitochondrial
Top non-mito. complexes
Other complexes

Appendix Figure S15: Onion-normalization with RPCA as dimensionality reduction method and maximum or average similarity as integration method applied to DepMap 20Q2 (Data ref: Broad DepMap, 2020)

A, Comparison of Precision-recall (PR) performance of original DepMap data, SNF (Wang *et al*, 2014) integrated RPCA-normalized layers (RPCO), average of RPCA-normalized layers and maximum across RPCA-normalized layers evaluated against CORUM complex standard. Layers are generated by setting RPCA hyperparameter λ to approximately 0.0049, 0.0056, 0.0063, 0.007, 0.0077, 0.0084, and 0.0091. The x-axis depicts the absolute number of true positives (TPs) recovered in log scale.

B, Contribution diversity plots from original DepMap data, SNF integrated RPCA-normalized layers (RPCO), average of RPCA-normalized layers and maximum across RPCA-normalized layers evaluated against CORUM complex standard. Note that the left panel is replicated from Figure 1C (right panel), 3C, 4B, and Appendix Figure S14B. The second panel is replicated from Figure 3C and 4B.

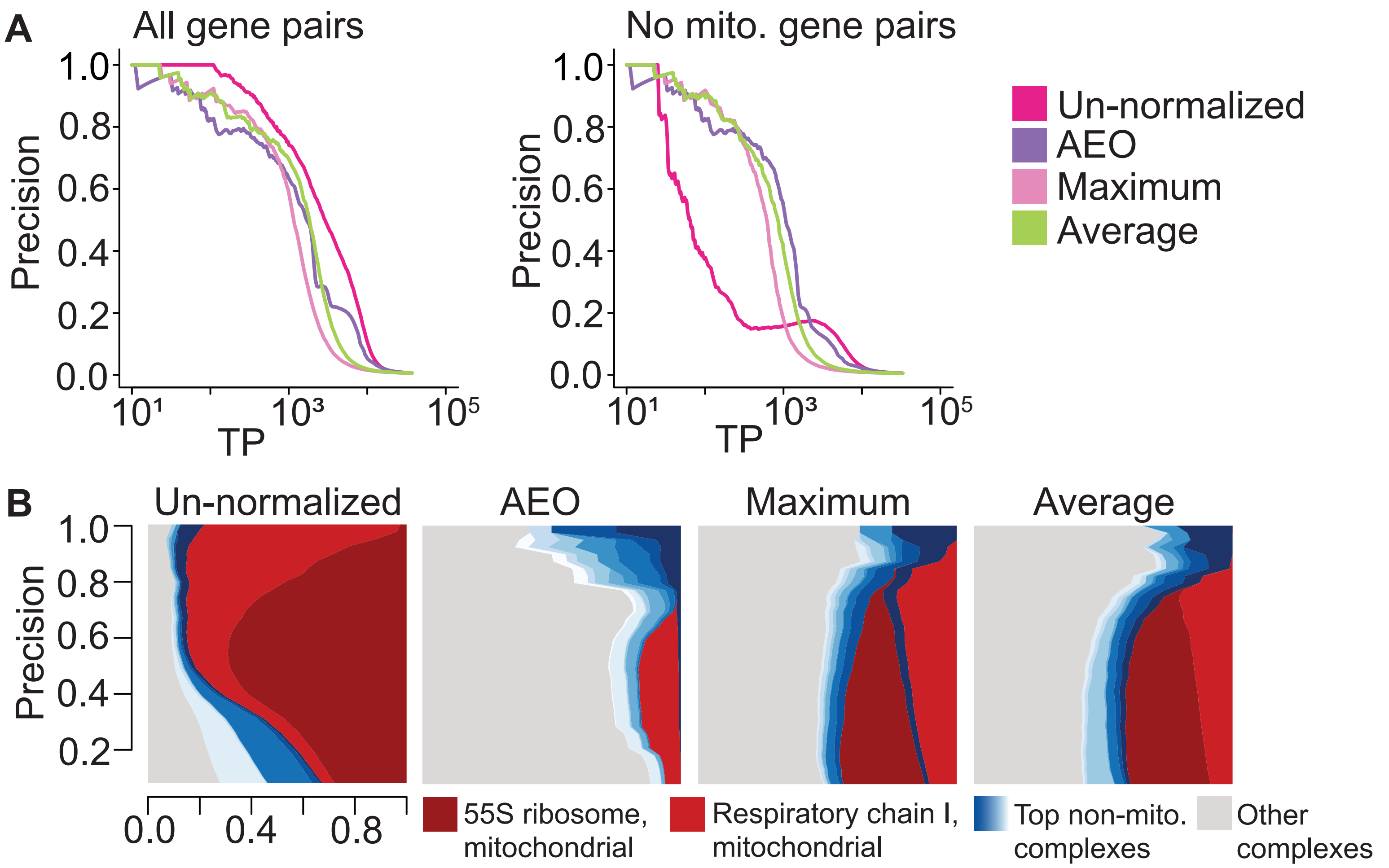
A
All gene pairs
No mito. gene pairs
Precision
1.0
0.8
0.6
0.4
0.2
0.0
TP
10^1
10^3
10^5
Un-normalized
AEO
Maximum
Average
B
Un-normalized
AEO
Maximum
Average
Precision
0.0
0.4
0.8
55S ribosome, mitochondrial
Respiratory chain I, mitochondrial
Top non-mito. complexes
Other complexes

Appendix Figure S16: Onion-normalization with AE as dimensionality reduction method and maximum or average similarity as integration method applied to DepMap 20Q2 (Data ref: Broad DepMap, 2020).

A, Comparison of Precision-recall (PR) performance of original DepMap data, SNF (Wang *et al*, 2014) integrated AE-normalized layers (AEO), average of RPCA-normalized layers and maximum across RPCA-normalized layers evaluated against CORUM complex standard. Layers are generated by dialing auto-encoder latent space size to 1, 2, 3, 4, 5, and 10. The x-axis depicts the absolute number of true positives (TPs) recovered in log scale.

B, Contribution diversity plots from original DepMap data, SNF integrated AE-normalized layers (AEO), average of RPCA-normalized layers and maximum across RPCA-normalized layers evaluated against CORUM complex standard. Note that the left panel is replicated from Figure 1C (right panel), 3C, 4B, and Appendix Figure S14B and S15B. The second panel is replicated from Figure 3C.

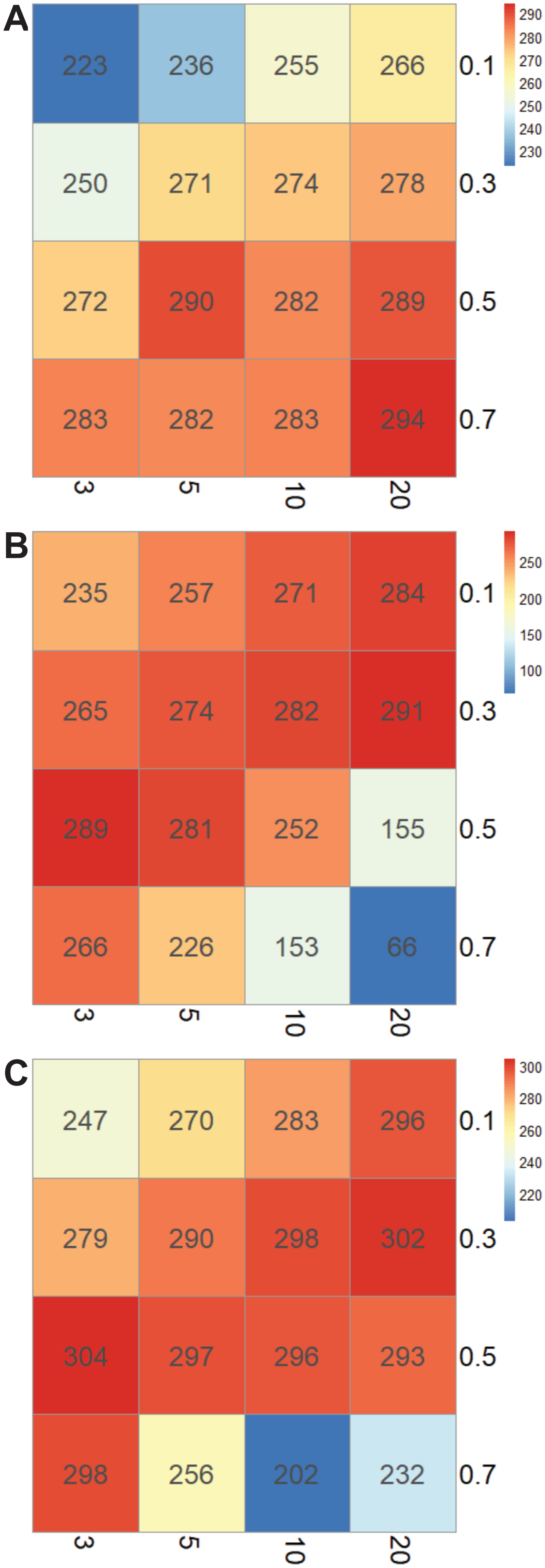
A
223
236
255
266
0.1
250
271
274
278
0.3
272
290
282
289
0.5
283
282
283
294
0.7
3
5
10
20
290
280
270
260
250
240
230
B
235
257
271
284
0.1
265
274
282
291
0.3
289
281
252
155
0.5
266
226
153
66
0.7
3
5
10
20
250
200
150
100
C
247
270
283
296
0.1
279
290
298
302
0.3
304
297
296
293
0.5
298
256
202
232
0.7
3
5
10
20
300
280
260
240
220

Appendix Figure S17: SNF hyperparameter exploration for onion normalization applied to DepMap 20Q2 (Data ref: Broad DepMap, 2020). The number of CORUM protein complexes which contribute any true positive pairs at a precision threshold of 0.5 are plotted for SNF parameters of σ = 0.1, 0.3, 0.5, 0.7 on the y-axis and k = 3, 5, 10, 20 on the x-axis.
A, Autoencoder-normalized data as input to onion normalization.
B, PCA-normalized data as input to onion normalization.
C, Robust PCA-normalized data as input to onion normalization.

References

Boyle EA, Pritchard JK & Greenleaf WJ (2018) High-resolution mapping of cancer cell networks using co-functional interactions. *Molecular Systems Biology* 14: e8594

Broad DepMap (2020) DepMap 20Q2 Public. (https://figshare.com/articles/DepMap_20Q2_Public/12280541/4) [DATASET]

Broad DepMap (2022) DepMap 22Q4 Public. (https://figshare.com/articles/dataset/DepMap_22Q4_Public/21637199/2) [DATASET]

Wainberg M, Kamber RA, Balsubramani A, Meyers RM, Sinnott-Armstrong N, Hornburg D, Jiang L, Chan J, Jian R, Gu M, *et al* (2021) A genome-wide atlas of co-essential modules assigns function to uncharacterized genes. *Nat Genet* 53: 638–649

Wang B, Mezlini AM, Demir F, Fiume M, Tu Z, Brudno M, Haibe-Kains B & Goldenberg A (2014) Similarity network fusion for aggregating data types on a genomic scale. *Nat Methods* 11: 333–337

5k Peripheral blood mononuclear cells (PBMCs) from a healthy donor (v3 chemistry), Single Cell Gene Expression Dataset by Cell Ranger 3.0.2. (2019) 10x Genomics (https://support.10xgenomics.com/single-cell-gene-expression/datasets/3.0.2/5k_pbmc_v3) [DATASET]
