# Supplementary material for: Dimensionality reduction methods for extracting functional networks from large‐scale CRISPR screens
Source: Mol Syst Biol. 2023 Sep 26;19(11):e11657. doi: 10.15252/msb.202311657 (PMC10632734; doi:10.15252/msb.202311657)
Supplement: Supplementary file 2 — Expanded View Figures PDF [file MSB-19-e11657-s004.pdf]

Expanded View Figures

Figure EV1. CORUM complex z-scores of AUPRC values for PCA normalization.

AUPRC values per complex (rows) for the un-normalized DepMap 20Q2 data (Data ref: Broad DepMap, 2020) (far left column) are compared to PCA-normalized DepMap data with the first 1, 3, 5, 7, 9, 11, 13, 15, 17, or 19 PCs removed. AUPRC values have been z-score-normalized per row for visualization purposes.

© 2023 The Authors

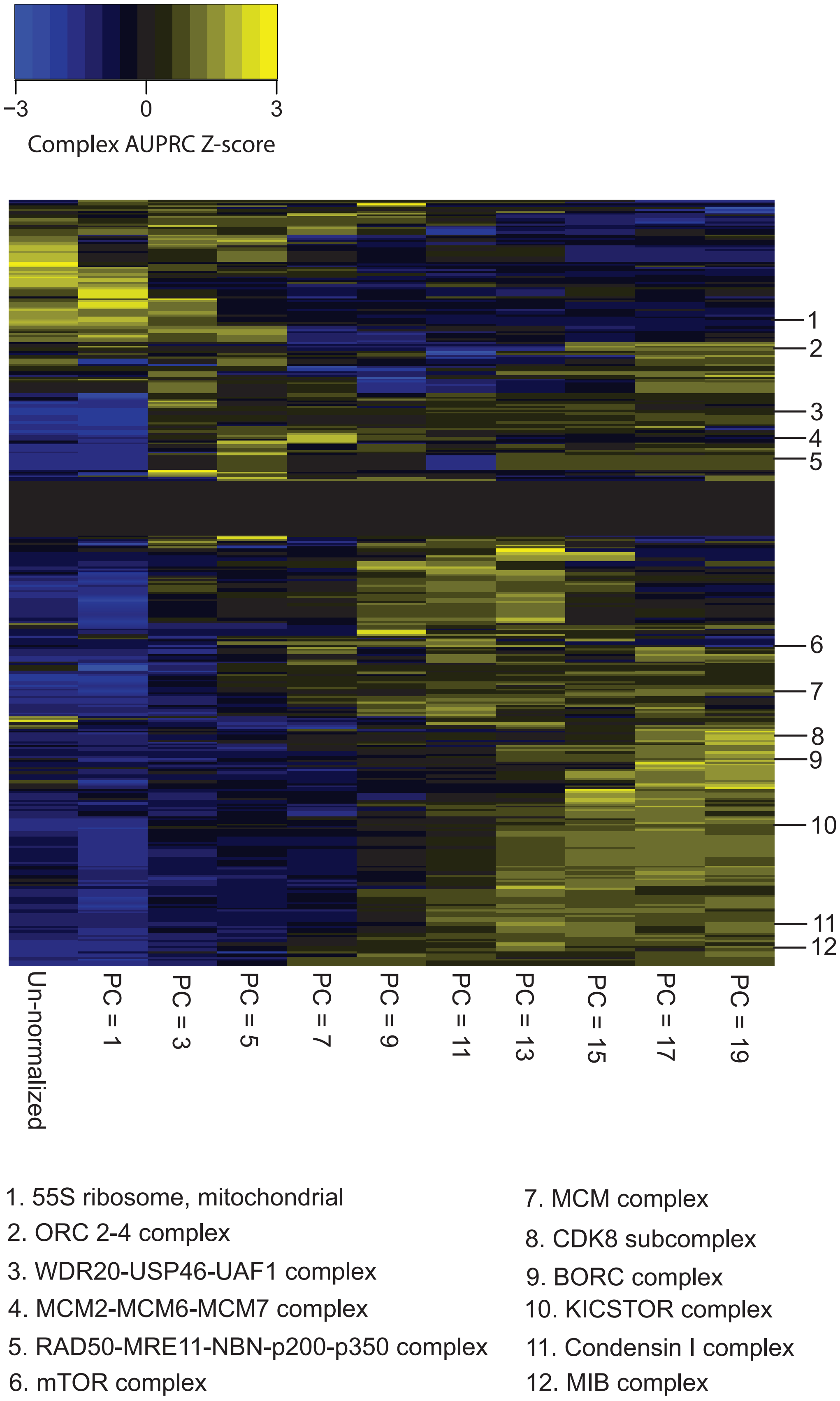

Figure EV1.

 © 2023 The Authors

Figure EV2. CORUM complex z-scores of AUPRC values for robust PCA normalization.

AUPRC values per complex (rows) for the un-normalized DepMap 20Q2 data (Data ref: Broad DepMap, 2020) (far left column) are compared to robust RPCA-normalized DepMap data for $\lambda \approx 0.0049, 0.0056, 0.0063, 0.007, 0.0077, 0.0084, 0.0091$. AUPRC values have been z-score-normalized per row for visualization purposes.

© 2023 The Authors

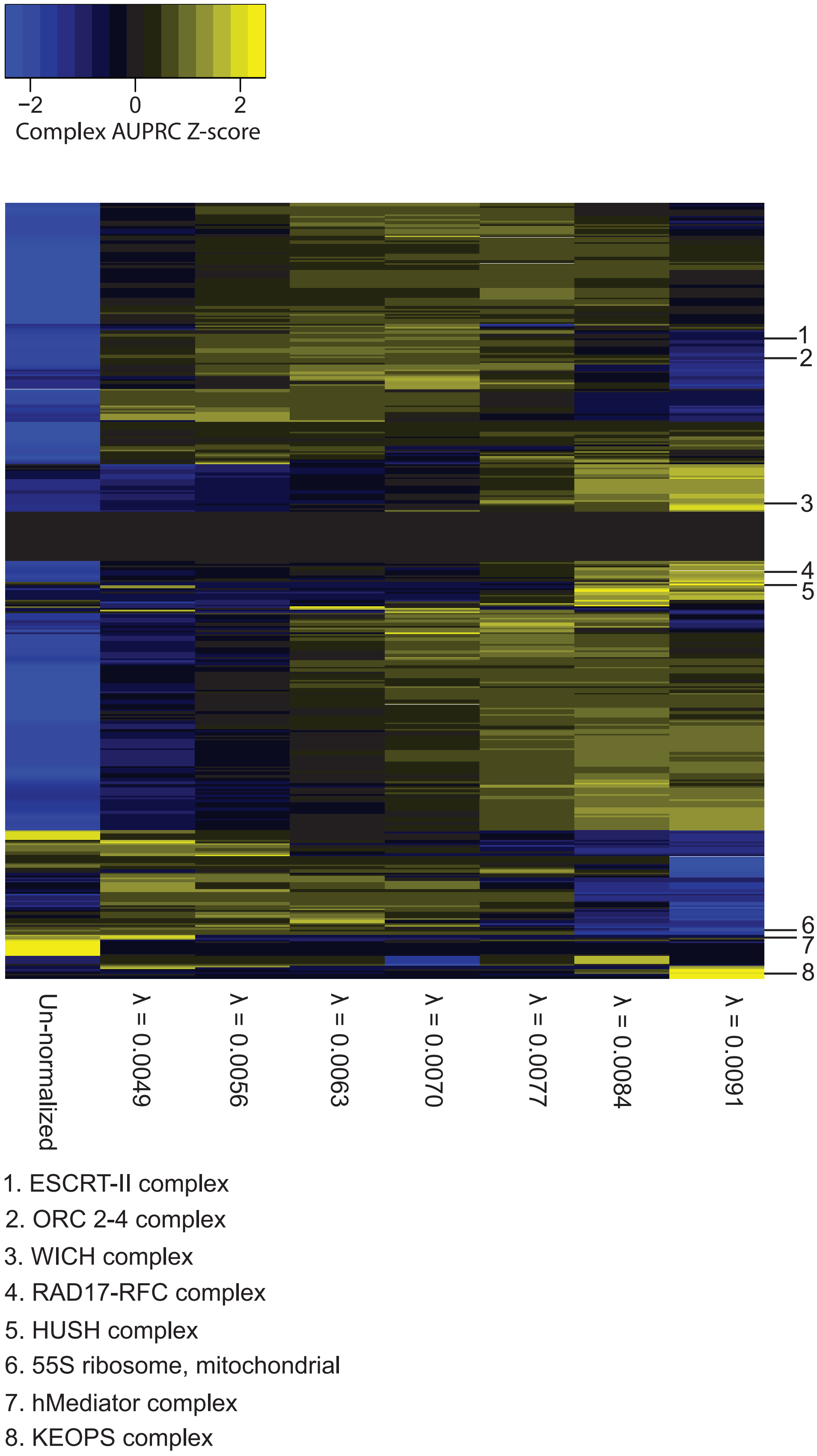

Figure EV2.

 © 2023 The Authors

Figure EV3. CORUM complex z-scores of AUPRC values for autoencoder normalization.

AUPRC values per complex (rows) for the un-normalized DepMap 20Q2 data (Data ref: Broad DepMap, 2020) (far left column) compared to AE-normalized DepMap data with latent space size (LS) = 1, 2, 3, 4, 5, 10. AUPRC values have been z-score-normalized per row for visualization purposes.

© 2023 The Authors

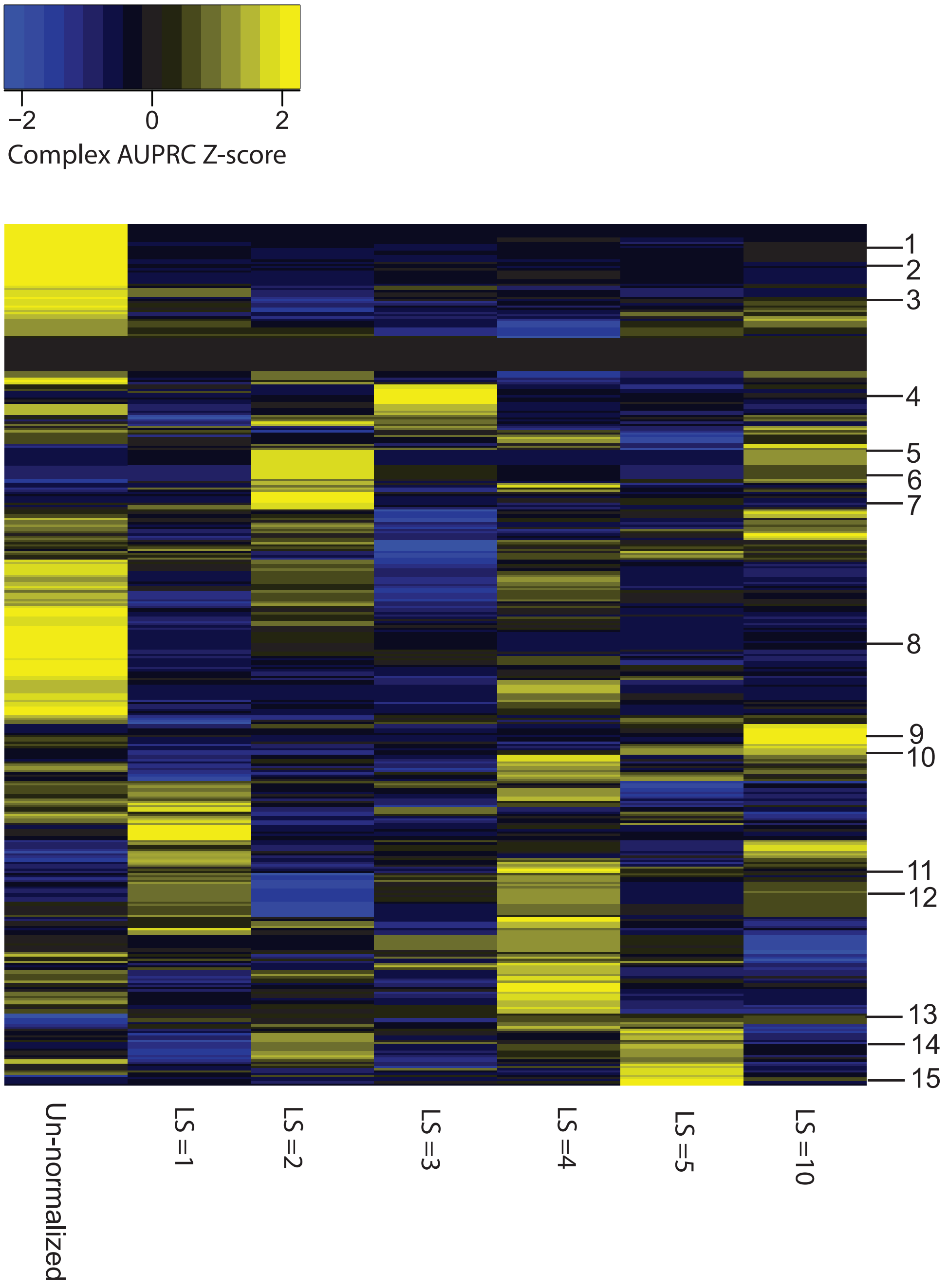

1. 55S ribosome
2. 39S ribosomal subunit
3. Respiratory chain complex
4. MBD1-MCAF1-SETDB1 complex
5. DNA-PK-Ku complex
6. DNA ligase IV-XRCC4 complex
7. LSm2-8 complex
8. mTOR complex
9. KATNA1-KATNAL1-KATNB1-KATNBL1 complex
10. Chromosomal passenger complex CPC
11. ANCO1-HDAC3 complex
12. BAF complex
13. Wave-2 complex
14. PAC3-PAC4 complex
15. FA complex

Figure EV3.

© 2023 The Authors

Figure EV4. *K*-nearest neighbor classifier tissue-lineage prediction results comparing un-normalized and RPCO-normalized DepMap 20Q2 (Data ref: Broad DepMap, 2020) cell line similarity networks.

A Overall F1, Precision, and Recall scores (weighted mean across classes) across different values of *K* (*x*-axis). Dashed line represents the scores from a baseline random classifier.

B Class-level (tissue-lineage) F1, Precision, and Recall scores for $K = 5$ where *x*-axis depicts tissue lineage.

© 2023 The Authors

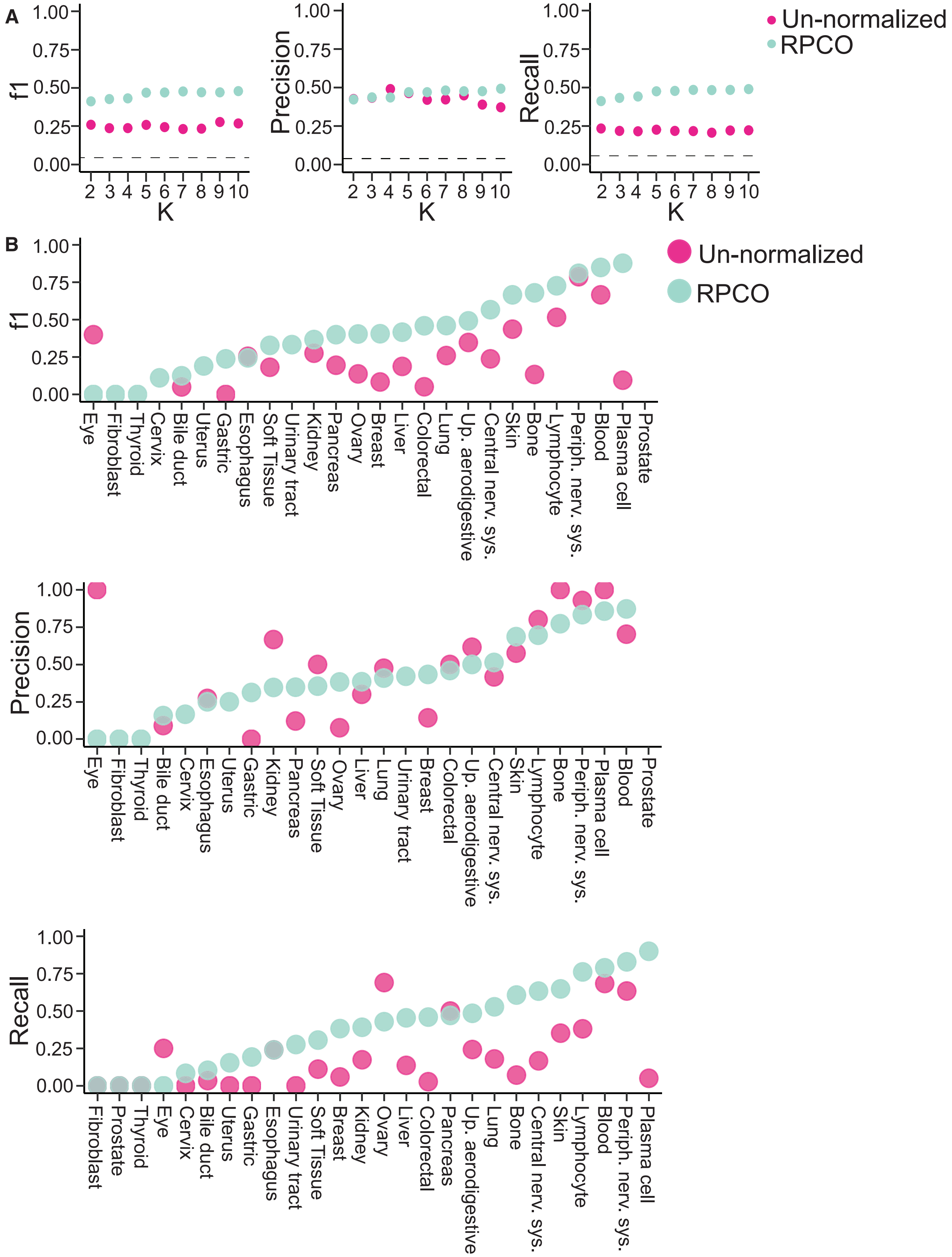

Figure EV4.

 © 2023 The Authors

Figure EV5. Confusion matrix from *K*-nearest neighbor classifier (K = 5) showing prediction results for each class (tissue lineage) of cell lines from un-normalized and RPCO-normalized DepMap 20Q2 (Data ref: Broad DepMap, 2020) cell line similarity networks.

X- and y-axes are class labels. The top row depicts the true number of cell lines in each class, and the right-most column is the number of predicted cell lines for each class. The diagonal represents true positives. Each row represents false positives, and each column represents false negatives sans the diagonal entry.

© 2023 The Authors

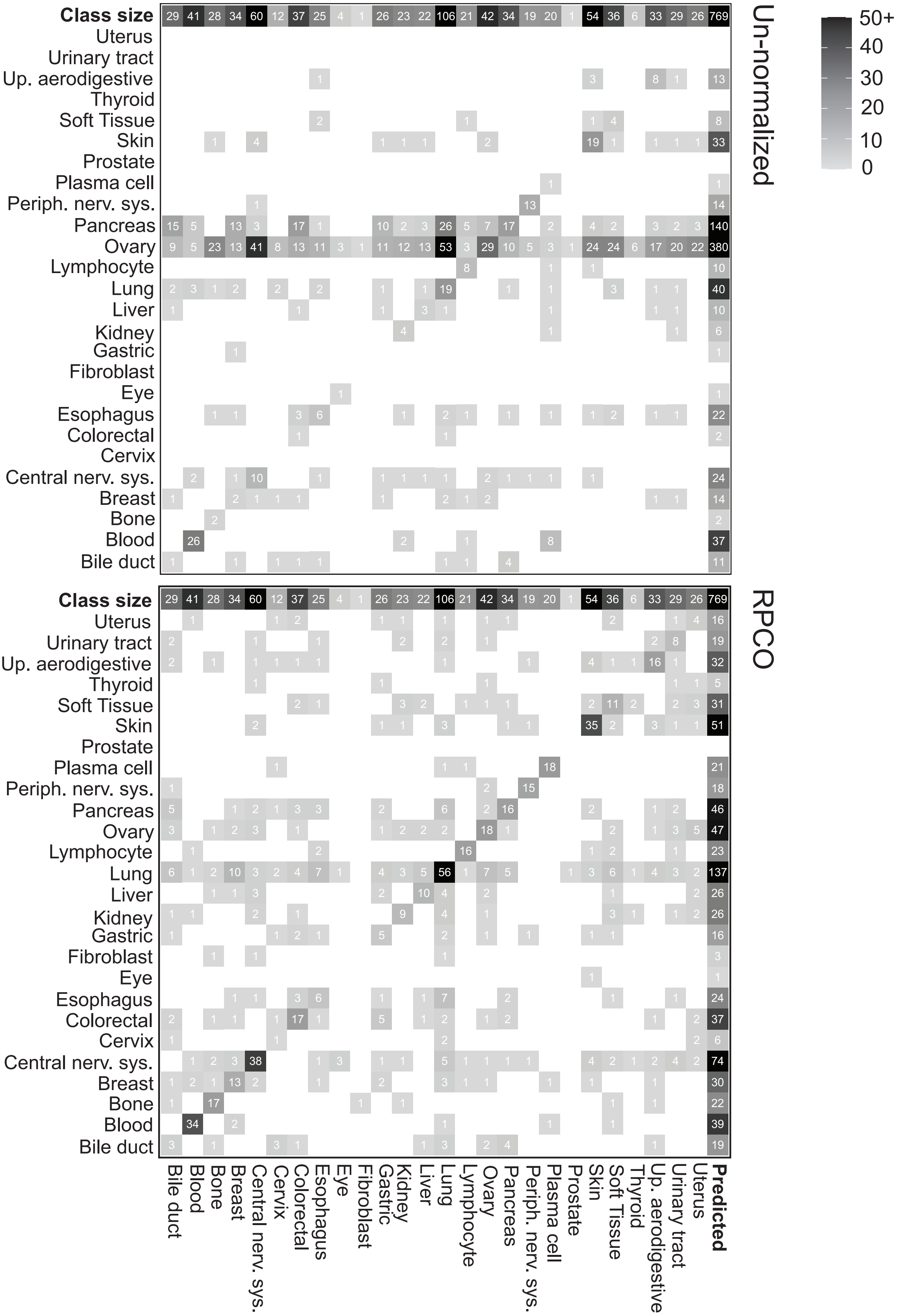

Figure EV5.

© 2023 The Authors
