# Supplementary material for: Dimensionality reduction methods for extracting functional networks from large‐scale CRISPR screens
Source: Mol Syst Biol. 2023 Sep 26;19(11):e11657. doi: 10.15252/msb.202311657 (PMC10632734; doi:10.15252/msb.202311657)
Supplement: Supplementary file 3 — PDF+ [file MSB-19-e11657-s002.pdf]

Method

TRANSPARENT PROCESS · OPEN ACCESS

molecular systems biology

Dimensionality reduction methods for extracting functional networks from large-scale CRISPR screens

Arshia Zernab Hassan[1,†], Henry N Ward[2,†], Mahfuzur Rahman[1], Maximilian Billmann[1,3], Yoonkyu Lee[2] & Chad L Myers[1,2,*]

Abstract

CRISPR-Cas9 screens facilitate the discovery of gene functional relationships and phenotype-specific dependencies. The Cancer Dependency Map (DepMap) is the largest compendium of whole-genome CRISPR screens aimed at identifying cancer-specific genetic dependencies across human cell lines. A mitochondria-associated bias has been previously reported to mask signals for genes involved in other functions, and thus, methods for normalizing this dominant signal to improve co-essentiality networks are of interest. In this study, we explore three unsupervised dimensionality reduction methods—autoencoders, robust, and classical principal component analyses (PCA)—for normalizing the DepMap to improve functional networks extracted from these data. We propose a novel "onion" normalization technique to combine several normalized data layers into a single network. Benchmarking analyses reveal that robust PCA combined with onion normalization outperforms existing methods for normalizing the DepMap. Our work demonstrates the value of removing low-dimensional signals from the DepMap before constructing functional gene networks and provides generalizable dimensionality reduction-based normalization tools.

Keywords auto-encoder; gene co-essentiality network; normalization; robust principal component analysis; unsupervised dimensionality reduction
Subject Categories Chromatin, Transcription & Genomics; Computational Biology; Methods & Resources
DOI 10.15252/msb.202311657 | Received 19 March 2023 | Revised 28 August 2023 | Accepted 5 September 2023 | Published online 26 September 2023
Mol Syst Biol. (2023) 19: e11657

Introduction

Deciphering the functional relationships among genes is imperative for understanding the mechanism of diseases with genetic components. Whole-genome CRISPR screening is one state-of-the-art method for identifying phenotype-specific genetic dependencies for diseases like cancer (Shalem *et al*, 2014; Wang *et al*, 2014b; Tsherniak *et al*, 2017). In addition to identifying cancer-specific dependencies, high-throughput data generated from whole-genome CRISPR screens can be mined to map functional relationships between genes (Boyle *et al*, 2018; Pan *et al*, 2018; Kim *et al*, 2019; Buphamalai *et al*, 2021; Wainberg *et al*, 2021). Therefore, the development of novel algorithms to process, normalize, and mine whole-genome CRISPR screening data could prove particularly fruitful for identifying such functional relationships.

Most CRISPR screens use CRISPR-Cas9 guides to introduce targeted knockouts across the vast majority of the human genome in human cell culture. In brief, the workflow for a typical screen involves the infection of human cell culture with a lentiviral vector containing a library of $\sim$ 70,000 guide (g)RNAs targeting around 18,000 genes. After passaging the cell population over several days, sequencing performed at various timepoints measures the dropout of gRNAs from the population. At the end of the experiment, computational analyses are performed to quantify observed fitness effects relative to controls, such as known non-essential guides or screens performed in wild-type cells. Current experimental techniques for performing whole-genome CRISPR screens are perhaps best exemplified by the Cancer Dependency Map (DepMap) project's efforts to discover genetic dependencies across human cell lines (Meyers *et al*, 2017; Tsherniak *et al*, 2017; Behan *et al*, 2019; Dempster *et al*, 2019a, 2019b; Dharia *et al*, 2021; Pacini *et al*, 2021). As of the 22Q4 version, the Cancer Dependency Map project had performed such CRISPR screens to identify cancer-specific genetic dependencies across 1,078 cell lines (Meyers *et al*, 2017; Dempster *et al*, 2019b, 2021; Pacini *et al*, 2021; Data ref: Broad DepMap, 2022).

In addition to directly identifying cancer-specific genetic dependencies, co-essentiality between genes can be measured and used to group genes into functional modules by measuring correlations between CERES scores in the DepMap—a type of analysis pioneered

1 Department of Computer Science and Engineering, University of Minnesota – Twin Cities, Minneapolis, MN, USA
2 Bioinformatics and Computational Biology Graduate Program, University of Minnesota – Twin Cities, Minneapolis, MN, USA
3 Institute of Human Genetics, University of Bonn, School of Medicine and University Hospital Bonn, Bonn, Germany
*Corresponding author. Tel: +1 612 624 8306; E-mail: chadm@umn.edu
†These authors contributed equally to this work

© 2023 The Authors. Published under the terms of the CC BY 4.0 license.

in the yeast genetic interaction research community (Baryshnikova et al, 2010; Costanzo et al, 2016). Indeed, this profile similarity analysis has been directly applied to the DepMap dataset to reveal functional similarities between human genes (Boyle et al, 2018; Pan et al, 2018; Kim et al, 2019; Buphamalai et al, 2021; Wainberg et al, 2021; Gheorghe & Hart, 2022). However, previous research has posited that profile similarities in the DepMap are confounded by technical variation unrelated to the cancer-specific phenotypes of interest (Rahman et al, 2021).

To address this problem, two methods for computationally enhancing cancer-specific signals and identifying the source of variation attributable to technical factors from the DepMap have been proposed. Boyle et al (2018) proposed to remove principal components derived from olfactory receptor gene profiles, which are assumed to contain variation irrelevant to cancer-specific dependencies, from the data. A separate method proposed by Wainberg et al to enhance signals within the DepMap applied generalized least squares (GLS) to account for dependence among cell lines (Wainberg et al, 2021). Our own functional evaluation of DepMap co-essentiality network using external gold-standards such as CORUM (Comprehensive Resource of Mammalian protein complex) protein co-complex annotations (Giurgiu et al, 2019) revealed substantial bias related to mitochondrial complexes, which dominate typical correlation analyses of DepMap profiles (Rahman et al, 2021). These signals are highly biologically relevant, but their dominance may eclipse contributions of genes in smaller complexes, which also represent cancer-specific dependencies. Because these existing normalization techniques have shown mixed results for boosting signal within smaller and non-mitochondrial complexes, in this study, we explore the use of unsupervised dimensionality reduction approaches for normalizing the DepMap dataset.

We explore classical principal component analysis (PCA; Wold et al, 1987) as well as two state-of-the-art dimensionality reduction normalization methods' abilities to boost the signal of cancer-specific dependencies and remove mitochondrial signal from the DepMap. Specifically, we apply a variant of PCA called robust PCA (RPCA; Candès et al, 2011) as well as autoencoder neural networks (AE; Hinton & Salakhutdinov, 2006) to learn and remove confounding low-dimensional signal from the DepMap. In addition, we propose a novel method named "onion" normalization as a general-purpose technique for integrating multiple layers of normalized data across different hyperparameter values into a single normalized network. The goal of the proposed onion normalization methods is to enable the construction of improved gene–gene similarity networks from the DepMap dataset, which has been a major recent focus of analyses of these data (Boyle et al, 2018; Wainberg et al, 2021; Gheorghe & Hart, 2022) but we note is distinct from other important applications of the DepMap goals such as direct clustering of the cell lines/genes (Pan et al, 2022), or more focused target/drug discovery-oriented analyses (Chiu et al, 2021; Ma et al, 2021; Shimada et al, 2021). We apply onion normalization using either PCA-normalized, RPCA-normalized, or AE-normalized data as input. Our benchmarking analyses of the normalized versions of the DepMap demonstrate that, while autoencoder normalization most efficiently captures and removes mitochondrial-associated signal from the DepMap, aggregating signals across different layers with onion normalization applied to RPCA-normalized networks is most effective at enhancing functional relationships between genes in the DepMap dataset.

Results

Removing low-dimensional signal from the DepMap boosts the performance of non-mitochondrial complexes

Dimensionality reduction techniques aim to transform a high-dimensional dataset into a low-dimensional one, and although they are typically applied under the assumption that low-dimensional signal is desirable (Way & Greene, 2017; Ding et al, 2018; Lopez et al, 2018; Lotfollahi et al, 2019; Sun et al, 2019), we flip that assumption in order to normalize DepMap data. We posit that two properties of the DepMap hold: we assume that true genetic dependencies are rare, based on estimations from large-scale yeast genetic interaction studies (Costanzo et al, 2016), and we assume that dominant low-dimensional signal in the DepMap is likely to represent mitochondrial-associated bias that is plausibly driven by technical variation or non-specific biological variation. For example, in the only genome-wide study of genetic interactions to date, it was estimated that an average gene interacts with others roughly 3% of the time (Costanzo et al, 2016). Therefore, instead of assuming that low-dimensional representations of DepMap data are desirable for data mining and visualization purposes, we instead propose to capture and remove that dominant signal from the DepMap (Fig 1A and B). We applied multiple dimensionality reduction methods to the DepMap to accomplish this goal, beginning with classical PCA normalization. To explore the extent to which normalization improves the detection of functional relationships between genes and removes mitochondrial bias from the DepMap, we applied benchmarking analyses with a software package developed for this purpose called FLEX (Rahman et al, 2021).

Benchmarking analyses with FLEX based on the CORUM protein complex standard (Giurgiu et al, 2019) reveal the extent of mitochondrial dominance in the DepMap for both the original dataset and all normalized versions. To summarize this benchmarking process, a gene-level similarity matrix is created from the per-gene dependency scores by calculating Pearson correlation coefficients (PCCs) between all pairs of genes. Taking these similarity scores and a set of gold standard co-annotations for genes as input, FLEX generates precision-recall curves (PR curves) that measure how many true positive gene pairs in the gold standard set are recapitulated by PCCs taken at different similarity thresholds. More detailed information such as which complexes drive the performance of PR curves are also output by FLEX and are illustrated graphically by diversity plots. To interpret these plots, a visually larger area corresponds to more contribution to the overall PR curve from a complex at the corresponding precision threshold. An examination of the original DepMap's CORUM PR curve performance alongside a diversity plot reveals that most performance in the PR curve is driven by two mitochondria-related complexes (Fig 1C). For example, the diversity plot shows that about 80% of the true positive gene pairs at precision point 0.8 are from gene pairs belonging to mitochondrial complexes. Specifically, the majority of true positive gene pairs at various precision cut-offs are annotated to be in 55S ribosome and respiratory chain mitochondrial complexes represented by the

 © 2023 The Authors

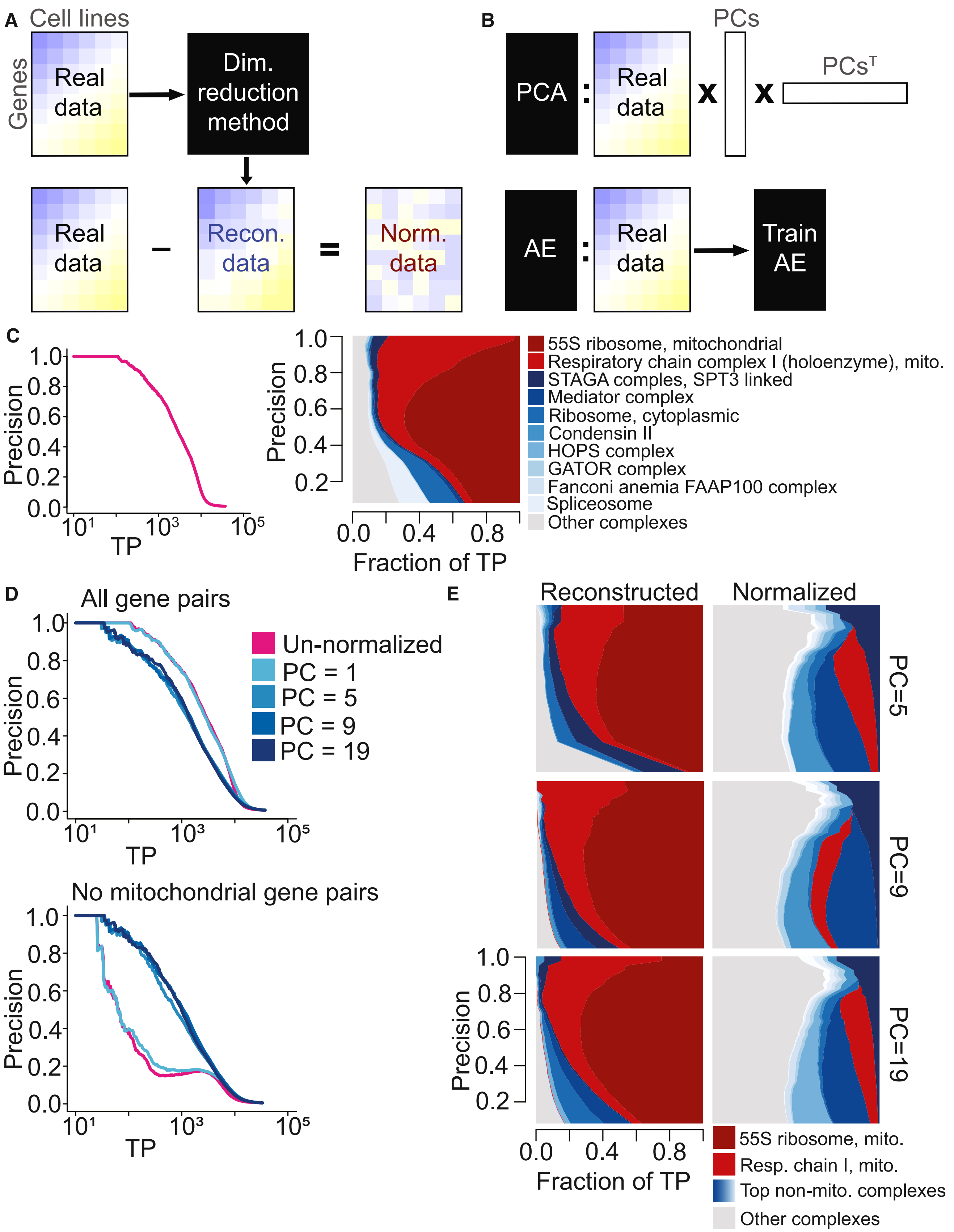
A
Cell lines
Genes
Real data
Dim. reduction method
Real data
Recon. data
Norm. data
B
PCA
Real data
PCs
PCs^T
AE
Real data
Train AE
C
Precision
TP
Fraction of TP
55S ribosome, mitochondrial
Respiratory chain complex I (holoenzyme), mito.
STAGA comples, SPT3 linked
Mediator complex
Ribosome, cytoplasmic
Condensin II
HOPS complex
GATOR complex
Fanconi anemia FAAP100 complex
Spliceosome
Other complexes
D
All gene pairs
Un-normalized
PC = 1
PC = 5
PC = 9
PC = 19
No mitochondrial gene pairs
E
Reconstructed
Normalized
PC=5
PC=9
PC=19
55S ribosome, mito.
Resp. chain I, mito.
Top non-mito. complexes
Other complexes

Figure 1.

Figure 1. Normalization schematic and exploration of mitochondrial bias within the DepMap with Principal Component Analysis (PCA) normalization.

A A dimensionality reduction method is applied to the original DepMap data to extract a low-dimensional representation of the data. Reconstructed data are generated from that, which is subtracted from the original DepMap to normalize it.

B (Top) Principal Component Analysis (PCA) generates reconstructed DepMap data by multiplying the DepMap against selected Principal Components (PC) derived from it and the transpose of those PCs. (Bottom) Autoencoders generate reconstructed data post-training by passing in the original DepMap as input.

C (Left) Precision-recall (PR) performance analysis of original DepMap 20Q2 data (Data ref: Broad DepMap, 2020) evaluated against CORUM protein complexes. The *x*-axis depicts the absolute number of true positives (TPs) recovered in log scale. (Right) Contribution diversity plot of CORUM complexes in un-normalized DepMap data. This plot is constructed by sliding a precision cutoff from high to low (indicated by the *y*-axis), and at each point, plotting a stacked bar plot across the *x*-axis at that point reflecting the breakdown of complex membership of the TP pairs identified at that threshold. The top 10 contributing complexes are listed in the legend, with the light gray category representing all complexes represented at lower frequency.

D (Top) Precision-recall (PR) performance analysis of PCA-normalized DepMap data with the first 5, 9, and 19 principal components removed evaluated against CORUM protein complexes. (Bottom) PR performance with mitochondrial gene pairs removed from evaluation. The *x*-axis of both plots depicts the absolute number of true positives (TPs) recovered in log scale.

E The contribution diversity plots depict contributions of TP pairs from various CORUM complexes in PCA-reconstructed data and PCA-normalized data generated by removing the first 5, 9, and 19 principal components.

large red area across the plot. These two complexes are the highest contributing complexes in terms of true positive pairs and contribute a disproportionate amount to the strong PR curve performance. Therefore, to ascertain how much signal the DepMap contains for all other protein complexes, we generated PR curves that exclude a set of mitochondrial gene pairs and observed a drastic but expected drop in overall performance (Fig 1D, Materials and Methods).

As a reference dimensionality reduction technique, we first examined the extent to which classical PCA (Wold *et al*, 1987) captures mitochondrial signal and boosts signal from other complexes post-normalization. In the PCA-normalization approach, PCA is first applied to gene perturbation profiles to capture low-dimensional signal. Then, the original dataset is projected onto a subset of the strongest PCs to generate a "reconstructed" version of the DepMap. Directly subtracting the reconstructed DepMap from the original DepMap produces a PCA-normalized version of the DepMap that does not contain the signal from the selected PCs.

While PCA normalization has already been applied to DepMap versions starting from 2019 Q3 to remove several principal components, this is insufficient to reduce the mitochondrial dominance of the dataset or to boost signal within smaller complexes (Meyers *et al*, 2017; Dempster *et al*, 2019b; Data ref: Broad DepMap, 2019a). Repeating analyses detailed in Rahman *et al* (2021), which analyzed the 18Q3 and 19Q2 versions of the DepMap, for the 20Q2 version, which is used for all analyses in this manuscript, reveals that co-dependency profiles are still dominated by mitochondrial signals (Meyers *et al*, 2017; Dempster *et al*, 2019b; Data ref: Broad DepMap, 2018, 2019b, 2020). In addition to removing this signal, successful normalization methods have the potential to uncover relationships masked by this signal, which can be measured by observing boosts in the performance of smaller complexes in terms of their contributions to CORUM PR curves.

Surprisingly, removing a large number of principal components from the DepMap improves the dataset's ability to capture signal within non-mitochondrial complexes (Fig 1D and E). We applied PCA-normalization to the DepMap 20Q2 dataset (Meyers *et al*, 2017; Dempster *et al*, 2019b; Data ref: Broad DepMap, 2020) and removed a varying number of principal components—either 1, 5, 9, or 19. In addition to generating standard CORUM PR curves with FLEX as described above, to measure the ability of each dataset to recover signal within non-mitochondrial complexes, we also generated PR curves where mitochondrial gene pairs were removed as positive examples from the CORUM standard (Fig 1D). While this only affects gene pairs where both genes are members of a set of 1,266 genes (see Materials and Methods), these mitochondrial-attenuated PR curves nevertheless reveal that removing 5 or more principal components boosts signal for non-mitochondrial complexes compared to the original DepMap. Diversity plots generated with FLEX confirm this observation (Fig 1E, Appendix Figs S1 and S2). We conclude that functional signal for most protein complexes remains and even improves while mitochondrial signal in the DepMap decreases after removing many principal components. These observations suggest that the strongest low-dimensional components of the DepMap are likely to represent technical variation, or at least non-specific variation that clouds more specific functional information, and that removing a large number of low-dimensional components is valuable in measuring other functional relationships.

In the following section, we introduce two state-of-the-art dimensionality reduction techniques for normalizing the DepMap before characterizing their ability to both reduce the dominance of mitochondrial-associated signal and boost the performance of smaller complexes.

Autoencoder and robust PCA normalization robustly capture and remove technical variation from the DepMap

Autoencoders are a type of deep neural network method designed for unsupervised dimensionality reduction (Hinton & Salakhutdinov, 2006). They function by optimizing the generation of reconstructed profiles that are similar to a training dataset after passing the training data through a neural network constructed in an "hourglass" shape. A crucial parameter of autoencoders is the latent space size, referred to as *LS* throughout, which is the number of nodes contained in the bottleneck layer at the center of the hourglass.

Strikingly, our analysis shows that deep convolutional autoencoders trained with a single-dimensional latent space can both generate realistic reconstructed profiles as well as capture and remove the majority of signal contributed by mitochondrial complexes in the DepMap. Similar to PCA normalization, after training the autoencoder and observing high gene-wise correlations between reconstructed profiles and the original profiles, we created AE-normalized data by directly subtracting the reconstructed matrix from the original data, thereby removing the low-dimensional signal. FLEX benchmarking shows that AE-normalized data for $LS = 1$, where the

© 2023 The Authors

bottleneck layer consists of only a single node, strongly reduces the dominance of mitochondrial complexes while boosting the signal of non-mitochondrial complexes (Fig 2A; Appendix Figs S3 and S4), similar to PCA normalization with many principal components. This provides evidence that the mitochondrial signal in the DepMap is low-dimensional and can be captured efficiently with an autoencoder model.

The second normalization technique that we apply to the DepMap is robust principal component analysis (Candès *et al*, 2011). RPCA, a modified version of PCA, is an unsupervised technique used to decompose a matrix into two components: a low-dimensional component and a sparse component, which are assumed to be superimposed. In this context, we expect the low-rank component to capture technical or non-specific biological variation and the sparse component to capture true genetic dependencies. Indeed, when we applied RPCA to the DepMap, it separated most of the dominant mitochondrial signals into the low-rank component (the "reconstructed" dataset) while the sparse component retained high-quality information about other functional relationships (the "normalized" dataset; Fig 2B; Appendix Figs S5 and S6). Dialing λ, a hyperparameter of RPCA, controls the dimensionality of the low-rank component, with smaller values increasing the dimensionality of the low-rank component.

Autoencoder and RPCA normalization consistently generated realistic reconstructed data and boosted the performance of smaller complexes across different values of LS and λ, respectively. Autoencoders trained with different values of LS generated reconstructed data with similarly high Pearson correlations to the original DepMap dataset, consistent with the observation that an autoencoder with a bottleneck layer consisting of a single node efficiently captures most mitochondrial signal in the DepMap. However, RPCA runs for larger values of λ resulted in reconstructed datasets with substantially improved correlation to the original DepMap, similar to the behavior of classical PCA (Fig 2C). Both autoencoder and RPCA normalization contributed consistent performance increases for non-mitochondrial complexes within CORUM PR curves (Fig 2A and B).

Interestingly, closer examination of the complexes with improved signal revealed that different complexes peaked in terms of performance at different hyperparameter settings for all methods (Figs EV1–EV3). Therefore, we sought to apply a method that could integrate normalized datasets across several different hyperparameter choices to maximize performance in detecting varied functional relationships in normalized data.

Onion normalization integrates normalized data across hyperparameter values

The final normalization technique we propose directly addresses this problem and involves the integration of several "layers" of normalized data—where different layers are versions of the DepMap normalized based on specific hyperparameter values, such as AE-normalized data for varying values of LS—in order to assimilate rare signals that may not be present in all layers of the data. The core assumption of "onion" normalization, which is supported by our previous analyses of both PCA-normalized and AE-normalized data, is that dialing the parameter values of a specific normalization method yields normalized gene effect scores containing information specific to individual layers as well as information common to multiple layers. As a result, similarity networks created using differently normalized networks may convey information with substantial variation, with each one capturing informative relationships between genes. Thus, to summarize the diverse information contained in separate layers of normalized data and to avoid computational and analytical redundancy, "onion" normalization aims to incorporate many different layers of normalized data into a single network.

We used a previously published, unsupervised technique called similarity network fusion (SNF) to perform this integration (Wang *et al*, 2014a). SNF operates by integrating several similarity networks using a network fusion technique based on multiview learning that considers the neighborhood and sparsity information of individual networks, which can integrate networks with subtle differences in an unbiased manner.

A key strength of onion normalization is that any effective dimensionality reduction method can be employed in the normalization step to generate different layers of the "onion." The similarity network layers to be fused are created from the same data normalized by varying key parameters of the chosen normalization method. For this study, we compared onion normalization applying PCA normalization with varying numbers of PCs (PCO), autoencoder normalization with varying latent space sizes (AEO), and RPCA normalization with varying lambda values (RPCO; Fig 3A).

FLEX benchmarking reveals that onion normalization improves performance compared to individual layers of normalized data for all normalization methods, with RPCO normalization showing the strongest performance of the three approaches (Fig 3B and D). Mitochondrial-attenuated PR curves reveal a substantial performance benefit for all onion-normalized datasets compared to the original DepMap. Moreover, due to improved performance for boosting weaker signal later in the PR curve (i.e., at thresholds corresponding to higher recall), RPCO outperforms both PCO and AEO (Fig 3B). Diversity plots of CORUM PR curves suggest that RPCO-normalization greatly reduces the mitochondrial dominance observed in the original DepMap dataset (Fig 3C; Appendix Fig S7). However, a closer analysis of the complexes driving the RPCO diversity plot reveals that, in addition to a partial reduction of mitochondrial-associated signal, signal within non-mitochondrial complexes is boosted such that the 10 complexes driving PR curve performance no longer include mitochondrial-associated complexes. Thus, rather than normalizing mitochondrial signal entirely out of the DepMap, RPCO normalization instead boosts signal within smaller, non-mitochondrial complexes such that the strongest gene–gene similarities are no longer dominated by mitochondria-related genes. All onion-normalized datasets also outperform their individual normalized layers for boosting signal within smaller complexes (Fig 3D).

A detailed analysis of complexes with boosted signal across normalization techniques shows that RPCO normalization best improves the signal contained in complexes with low signal in the original DepMap. We plotted the number of complexes with strongly boosted or weakened signal, defined as those with AUPRCs that differ at selected AUPRC cutoffs (either 0.1 or 0.5) in normalized data compared to the original DepMap, and binned those across complex size for all normalization techniques (Fig 3D). This analysis shows that integration with onion normalization, especially with RPCA, outperforms all individually normalized layers at boosting

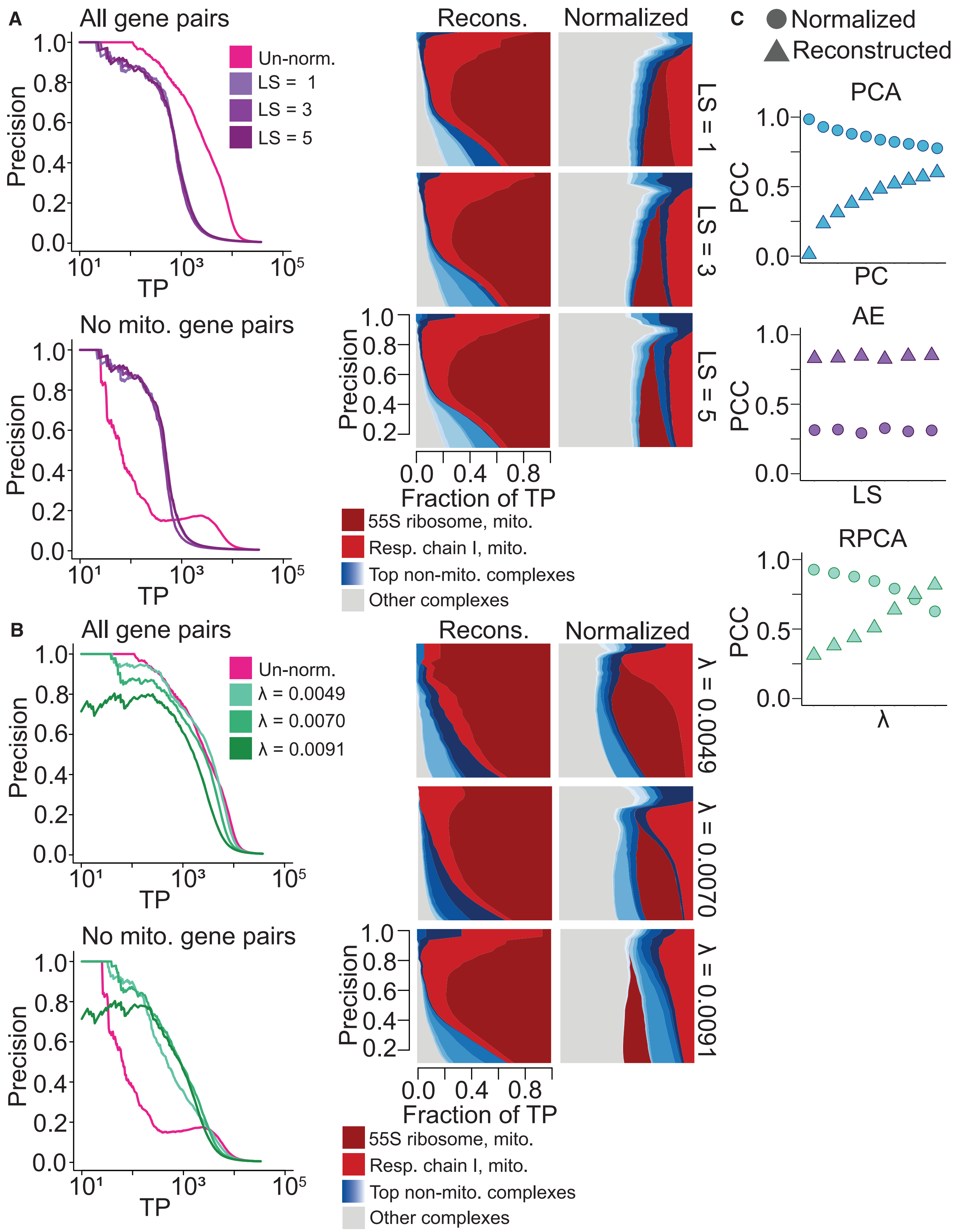

Figure 2.

 © 2023 The Authors

◀ **Figure 2. Exploration of Principal Component Analysis (PCA), robust PCA (RPCA), and autoencoder (AE) normalization across hyperparameters.**

A (Left) Precision-recall (PR) performance analysis of AE-normalized DepMap 20Q2 data (Data ref: Broad DepMap, 2020) generated with latent space sizes 1, 3 and 5 evaluated against CORUM protein complexes. The *x*-axis depicts the absolute number of true positives (TPs) recovered in log scale. (Right) Corresponding contribution diversity plots depicting TP pairs contributions from various CORUM complexes in AE-reconstructed and AE-normalized data.

B (Left) PR performance analysis of RPCA-normalized DepMap data generated with hyperparameter λ set to 0.0049, 0.007 and 0.0091 evaluated against CORUM protein complexes. The *x*-axis depicts the absolute number of true positives (TPs) recovered in log scale. (Right) Corresponding contribution diversity plots illustrating TP pairs' contributions from CORUM complexes in RPCA-reconstructed and RPCA-normalized data.

C Scatter plot of Pearson's correlation coefficients between un-normalized DepMap data and reconstructed data as well as between un-normalized data and normalized data generated by PCA, AE, and RPCA normalization. *Y*-axis contains Pearson's correlation coefficient values, and the *x*-axis contains the number of removed principal components (first 1, 3, 5, 7, 9, 11, 13, 15, 17, 19) for PCA-normalization, latent space sizes (1, 2, 3, 4, 5, 10) for AE normalization and λ (approximately 0.0049, 0.0056, 0.0063, 0.007, 0.0077, 0.0084, 0.0091) for RPCA normalization.

the signal contained across complexes of different sizes. For example, even though autoencoder normalization efficiently removes mitochondrial signal, it also removes signal from many non-mitochondrial complexes—a drawback rescued by integration with onion normalization.

Similar benchmarking analyses show that RPCO and AEO normalization outperform the GLS normalization technique proposed by Wainberg *et al* (2021) and the olfactory receptor normalization (OLF) technique proposed by Boyle *et al* (2018). Mitochondrial-attenuated PR curves show improved performance of RPCO over AEO and GLS, which perform similarly (Fig 4A), while diversity plots reveal that both AEO and RPCO reduce mitochondrial-associated signal more distinctly than GLS (Fig 4B; Appendix Fig S8). Plotting per-complex AUPRC values based on the difference between normalized and un-normalized data for all methods details a similar pattern for thresholds of 0.1 and 0.5, where RPCO performs best and AEO and GLS perform similarly (Fig 4C). For the complexes with the most pronounced difference between unnormalized and normalized data at a threshold of 0.7 AUPRC, both RPCO and AEO perform similarly and substantially outperform GLS. Although several individual normalized layers from RPCA, PCA, and AE perform comparably to GLS, the combination of all layers (RPCO) results in the strongest performance and outperforms GLS (Appendix Figs S9 and S10). Across all evaluations, OLF normalization does not substantially reduce mitochondrial signal or boost signal contained within non-mitochondrial complexes compared to the other three methods. Furthermore, we found similar performance from RPCA- and RPCO-normalization techniques when applied to a more recent version of the DepMap (DepMap 2022 Q4 Chronos scores; Meyers *et al*, 2017; Dempster *et al*, 2019b, 2021; Pacini *et al*, 2021; Data ref: Broad DepMap, 2022) and benchmarked against GLS, confirming that the RPCO-normalization is robust across DepMap scoring pipelines (Appendix Figs S11 and S12).

Network analysis of onion-normalized DepMap data uncovers biologically relevant clusters

To visually examine functional relationships between genes pre- and post-RPCO normalization and the expected reduction in mitochondrial signal, we created correlation networks for both versions of the DepMap in Cytoscape version 3.7.2 (Shannon *et al*, 2003) using the yFiles organic layout algorithm. We performed this for five, ten, and fifteen thousand of the top-ranked edges sorted in decreasing order of correlations for pre- and post-normalization data, plotting the five and fifteen-thousand edge networks (Fig 5A and B). Rather than forming a handful of connected components centered around hub genes, RPCO-normalized data formed up to 2,073 discrete clusters for the fifteen-thousand edge network (Fig 5A). However, pre-normalization DepMap data represented nearly an order of magnitude fewer clusters for the fifteen-thousand edge network, 290, with the majority of edges instead concentrated into a single connected component with many mitochondrial-associated edges (Fig 5B). Comparing the number of genes represented across networks further illustrates that RPCO normalization uncovers relationships previously masked by mitochondrial-associated signal, with 10,493 more genes in the fifteen thousand edge RPCO network than the corresponding pre-normalization network (Fig 5C).

An investigation of clusters derived from RPCO-normalized data which lack signal in the original DepMap reveals potentially novel functions for the genes KPRP, DNTTIP1, TMEM59L, and ELMSAN1. Twelve out of thirteen of a cluster of genes with a mean z-score of 43.8 in RPCO-normalized data, compared to a z-score of 1.9 in the original DepMap, are enriched for GO terms related to metal homeostasis (Fig 5D). The remaining gene, KPRP, is mostly uncharacterized and is not annotated to any GO biological process term. Therefore, we hypothesize that KPRP is also involved in metal homeostasis, perhaps working in conjunction with its nearest neighbor MT1X. A separate cluster of twelve genes, with a z-score of 30 in RPCO-normalized data compared to a z-score of 1.5 in the original DepMap, is enriched for MAP kinase signaling-related genes such as MAPK14 (Fig 5E). Intriguingly, while the gene ELMSAN1 (since renamed to MIDEAS) is known to be involved with histone deacetylation but little else, it is connected to both MAPK14 and MAP2K3. Through these connections, the similarly uncharacterized genes DNTTIP1 and TMEM59L are associated with this cluster as well, indicating a potential connection between ELMSAN1, DNTTIP1, TMEM59L, and MAPK14 activity.

Onion normalization improves prediction of cell lines' tissue-of-origin

Our previous analyses focused on refinement of gene similarity networks derived from the DepMap data. We reasoned that onion normalization may also improve detection of similarities between cell lines' dependency profiles. Previous work has explored the extent to which cell lines with similar mutations or similar tissues-of-origin exhibit common dependencies (e.g., Dharia *et al*, 2021). To test this, we implemented a simple *K*-nearest neighbor (kNN) classifier to predict tissue-of-origin from dependency profiles and optimized the

© 2023 The Authors

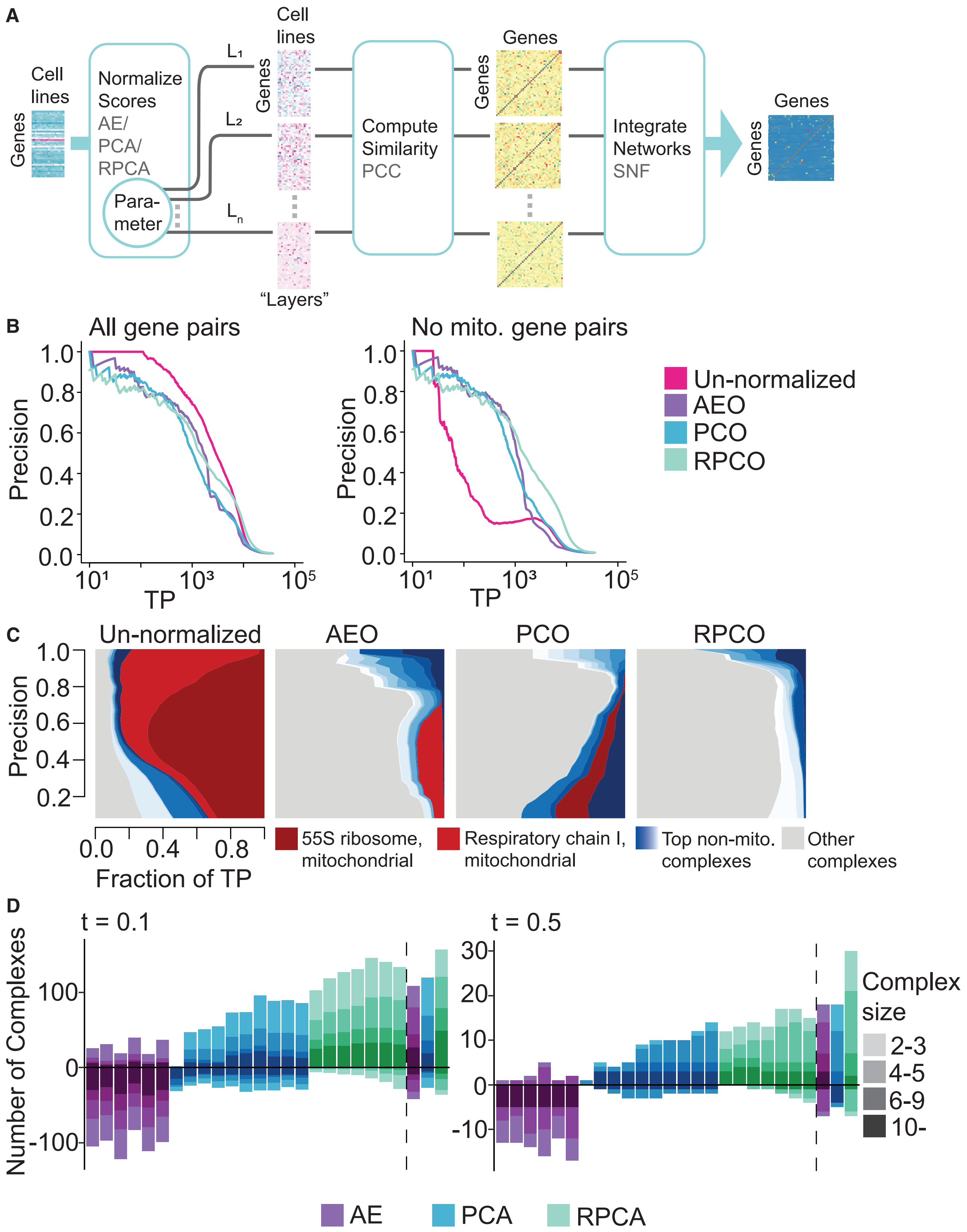

Figure 3.

 © 2023 The Authors

Figure 3. Onion normalization schematic and benchmarking for different normalization techniques as input.

A Similarity networks are generated from data normalized with a chosen technique—autoencoders (AE), Principal Component Analysis (PCA) or robust PCA (RPCA)—for different choices of hyperparameters, which are then combined with a network integration technique.

B FLEX precision-recall (PR) performance analysis of original DepMap 20Q2 data (Data ref: Broad DepMap, 2020) and onion normalized data with AE (AEO), PCA (PCO) or RPCA (RPCO) as normalization methods against CORUM protein complexes as the standard. (Left) All CORUM co-complex gene pairs as true positives. (Right) Mitochondrial gene pairs are removed from the evaluation. The *x*-axis of both plots depicts the absolute number of true positives (TPs) recovered in log scale.

C Contribution diversity of CORUM complexes for the original DepMap, AEO, PCO, and RPCO data. Fractions of true positives (TP) from different complexes are plotted at various precision levels on the *y*-axis. Note that the left panel is replicated from Fig 1C (right panel).

D Number of complexes for which area under the PR curve (AUPRC) values increase and decrease with respect to chosen AUPRC thresholds due to normalization as compared to un-normalized data. The bars on the left side of the dotted line correspond to AE-normalized layers (latent space size = 1, 2, 3, 4, 5, 10), PCA-normalized layers (first 1, 3, 5, 7, 9, 11, 13, 15, 17, 19 principal components removed) and RPCA layers ($\lambda \approx$ 0.0049, 0.0056, 0.0063, 0.007, 0.0077, 0.0084, 0.0091). The bars on the right side of the dotted line correspond to SNF integrated data of the respective layers for all three methods. The color gradient for each method represents four bins with complexes containing 2–3 genes, 4–5 genes, 6–9 genes, and 10 or more genes. (Left) $t = 0.1$. (Right) $t = 0.5$.

choice of k (see Materials and Methods). The kNN classifier was provided either similarity based on the un-normalized dataset, or a similarity network derived from RPCO normalization applied to the cell line similarity matrix based on the DepMap 20Q2 dataset (Meyers *et al*, 2017; Dempster *et al*, 2019b; Data ref: Broad DepMap, 2020). We evaluated precision-recall statistics for each possible tissue-of-origin, which reflects the ability of the kNN to correctly predict the corresponding tissue-of-origin based on each cell line's nearest neighbors. We found that the RPCO-normalized network supported a substantial increase in the median F1 score for tissue-of-origin prediction (from 0.2 to 0.4 for $k = 5$) and for 24 out of 26 tissues, the normalization resulted in equal or better performance (Figs EV4 and EV5). This indicates that cell line similarity networks also benefit from onion normalization.

Onion normalization enhanced signals in gene expression data

To explore the generalizability of our onion normalization methods to other genome-scale datasets, we applied onion normalization to a single-cell gene expression dataset generated from healthy peripheral blood mononuclear cells (PBMCs) using Chromium scRNA-seq technology and Cell Ranger (Zheng *et al*, 2017; Data ref: 10x Genomics, 2019). The pre-processed data contain log-normalized expression readouts for 12,410 genes across 1,195 cells. A FLEX PR curve from the un-normalized data benchmarked against the CORUM protein standard shows the detection of 2,000 true positive gene pairs at a precision threshold of 0.8 (Appendix Fig S13A). However, the corresponding diversity plot shows that the majority of the strong performance (high precision) indicated by the PR curve comes from the cytoplasmic ribosome complex. PR-curves from RPCA- and RPCO-normalized data outperform un-normalized data by increasing the number of true positive (TP) gene pairs from 2,000 to 5,000 at a precision threshold of 0.8 (Appendix Fig S13B left). Moreover, PR curves without ribosomal gene pairs in the evaluation reveal that the normalized data performs better than the un-normalized data (Appendix Fig S13B right). For example, at a precision threshold of 0.2 the un-normalized data have around 50 TP gene pairs, whereas RPCO normalization has 100. This suggests that the normalization process enhances signals in gene pairs within non-ribosomal complexes. For example, a closer look at the per-complex AUPRC values reveals that RPCO normalization increased AUPRC for the Ferritin complex from 0.036 to 0.25 and for the Cofilin-actin-CAP1 complex from 0.028 to 0.146. This indicates that an optimized onion normalization method can be used to generally boost signals in gene expression data as well as CRISPR screen data.

Discussion

In this study, we explored the use of unsupervised dimensionality techniques to identify functional relationships between genes within whole-genome CRISPR screening data and proposed a novel method called "onion" normalization for integrating signal between different "layers" of normalized data. While deep learning with autoencoders efficiently removed unwanted mitochondrial signal from the DepMap, this performance came at the expense of signal within smaller, non-mitochondrial complexes. Onion normalization rescued this poor performance for small complexes while still reducing mitochondrial signal and outperformed all proposed and state-of-the-art normalization methods when paired with robust principal component analysis (RPCO).

Co-essentiality maps derived from RPCO-normalized data show an unprecedented ability to recover signal from most of the genome when contrasted against the un-normalized DepMap and previous DepMap-derived co-essentiality maps. The fifteen-thousand edge RPCO network, constructed in a completely unsupervised way by measuring Pearson correlations above a given threshold, contained a total of ~ 12 k genes with an average of 2.5 neighbors per gene. The same approach applied to the original DepMap captured only ~ 1,500 genes with an average of 19.7 neighbors per gene, likely due to the dominance of mitochondrial-associated hub genes within the network. Previous co-essentiality maps constructed from the DepMap either filtered out the majority of the genome or initialized the network structure based on a set of pre-existing clusters (Kim *et al*, 2019; Wainberg *et al*, 2021), techniques ill-suited for mapping the functions of understudied genes. RPCO-normalization overcomes these limitations and allows us to ascribe putative functions to previously weakly connected genes.

We emphasize that the main purpose of the proposed onion normalization methods is to enable the construction of improved gene–gene similarity networks from the DepMap dataset. Our analysis also suggests it can also be used for refining cell line-level similarity networks (e.g., for identification of cell lines that exhibit common dependencies). However, there are many other important applications of the DepMap data including direct clustering of the cell lines/genes, more focused target/drug discovery-oriented analyses,

© 2023 The Authors

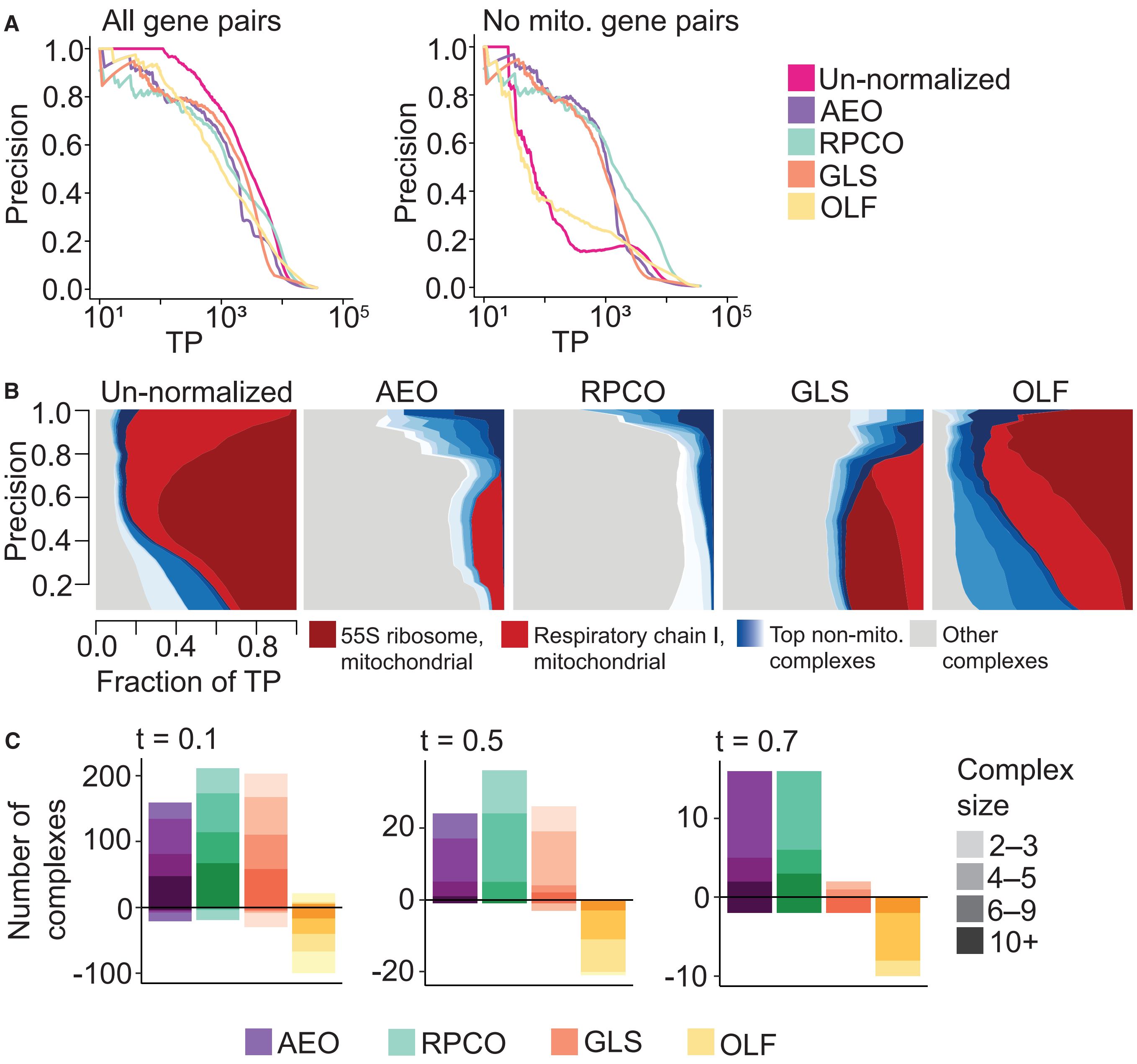

Figure 4. Benchmarking onion normalization against other methods.

A FLEX precision-recall (PR) performance analysis of original DepMap 20Q2 data (Data ref: Broad DepMap, 2020) and data from onion normalization with autoencoders (AEO), onion normalization with robust PCA (RPCO), generalized least squares (GLS) normalization from Wainberg *et al* (2021), and olfactory receptor (OLF) normalization from Boyle *et al* (2018) against CORUM protein complexes as the standard. (Left) All CORUM co-complex gene pairs as true positives. (Right) Mitochondrial gene pairs are removed from the evaluation. The *x*-axis of both plots depicts the absolute number of true positives (TPs) recovered in log scale.

B Contribution diversity of CORUM complexes for the original DepMap, AEO, RPCO, GLS, and OLF data. Fractions of true positives (TP) from different complexes are plotted at various precision levels on the *y*-axis. Note that the left panel is replicated from Fig 1C (right panel) and 3C, and the second and third panels are replicated from Fig 3C.

C Number of complexes for which area under the PR curve (AUPRC) values increase and decrease with respect to chosen AUPRC thresholds due to normalization as compared to un-normalized data for AEO, RPCO, GLS, and OLF data. The color gradient for each method represents four bins with complexes containing 2–3 genes, 4–5 genes, 6–9 genes, and 10 or more genes. (Left) $t = 0.1$. (Middle) $t = 0.5$. (Right) $t = 0.7$.

or analysis of individual genetic dependencies identified by the DepMap profiles. Onion normalization is not applicable to many of those other downstream applications.

Our exploration provides a compendium of resources for studying functional relationships within the DepMap at an improved resolution, including a novel co-essentiality map and the onion

 © 2023 The Authors

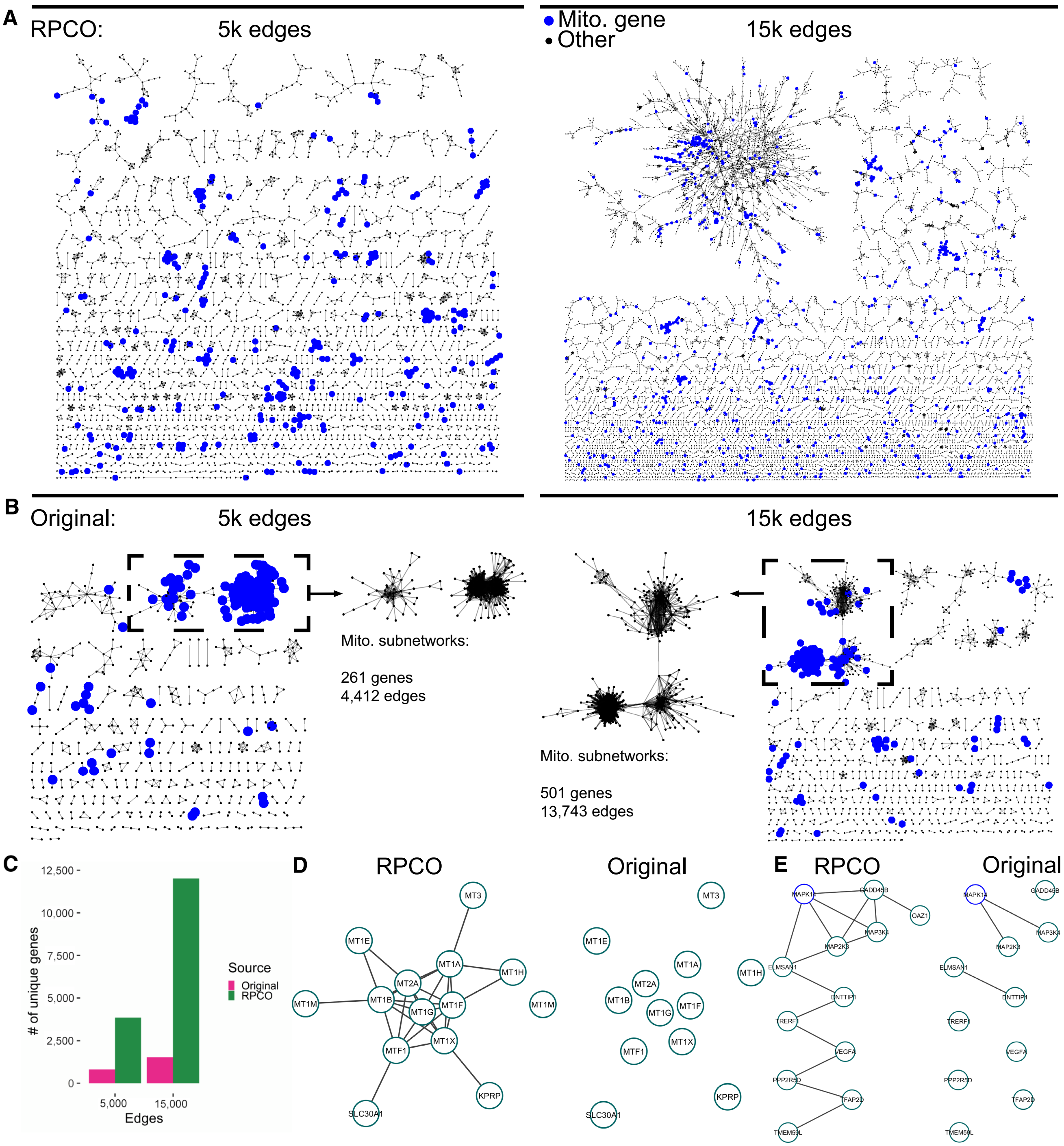

Figure 5. Network analysis of RPCO-normalized and original DepMap data.

A Top-ranked edges between genes from RPCO-normalized DepMap 20Q2 data (Data ref: Broad DepMap, 2020) laid out with the yFiles organic layout algorithm in Cytoscape (Shannon *et al*, 2003), with mitochondrial-associated genes highlighted in blue. (Left) The top 5,000 edges for $n = 3{,}850$ genes. (Right) The top 15,000 edges for $n = 12{,}017$ genes.

B Top-ranked edges based on Pearson correlations between genes from original DepMap data laid out with the yFiles organic layout algorithm in Cytoscape, with mitochondrial-associated genes highlighted in blue. The largest connected components of the networks are inset and represent many mitochondrial-associated genes. (Left) The top 5,000 edges for $n = 810$ genes. (Right) The top 15,000 edges for $n = 1{,}524$ genes.

C The number of genes represented in the RPCO and original DepMap networks plotted in panels A and B.

D Cluster derived from the 15,000 edge RPCO network representing metal homeostasis genes. (Left) Edges present in 15,000 edge RPCO network. (Right) Edges present in 15,000 edge original DepMap network.

E MAPK14-centric cluster derived from the 15,000 edge RPCO network. (Left) Edges present in 15,000 edge RPCO network. (Right) Edges present in 15,000 edge original DepMap network.

© 2023 The Authors

normalization method. While our results show a strong performance benefit for robust principal component analysis, future work could investigate both deep learning approaches for normalizing the DepMap and onion normalization applied to different input normalization approaches. Perhaps other deep-learning approaches that learn meaningful latent spaces, such as variational autoencoders (Kingma & Welling, 2022), could better learn and remove mitochondrial signal without reducing signal within mitochondrial-associated complexes. As the key technical limitation of onion normalization is its high memory cost, which scales with the number of layers, future work could also investigate the choice of optimal hyperparameters across different layers of normalized data. Additionally, onion normalization is a general framework that our initial analyses suggest may be applicable to other types of genomic data such as bulk and single-cell RNA-seq.

Materials and Methods

Reagents and Tools table

Reagent/Resource	Source	Identifier
**Dataset**		
DepMap 2020 Q2 Genome-wide CRISPR screens	https://figshare.com/articles/DepMap_20Q2_Public/12280541/4	N/A
• Achilles_gene_effect.csv	Meyers *et al* (2017), Dempster *et al* (2019b), Data ref: Broad DepMap (2020)	
• sample_info.csv		
DepMap 2022 Q4 Genome-wide CRISPR screens	https://figshare.com/articles/dataset/DepMap_22Q4_Public/21637199/2	N/A
• CRISPRGeneEffect.csv	Meyers *et al* (2017), Dempster *et al* (2019b, 2021), Pacini *et al* (2021), Data ref: Broad DepMap (2022)	
10x Genomics scRNA-seq gene expression dataset	https://support.10xgenomics.com/single-cell-gene-expression/datasets/3.0.2/5k_pbmc_v3	N/A
• 5k_pbmc_v3_filtered_feature_bc_matrix.tar	Data ref: 10x Genomics (2019)	
**Software**		
argparse R package v2.0.3	https://cran.r-project.org/web/packages/argparse/index.html<br>Davis *et al* (2023)	N/A
clusterProfiler R package v3.16.1	Wu *et al* (2021)	N/A
crfsuite R package v0.4.1	https://cran.r-project.org/web/packages/crfsuite/index.html<br>Wijffels *et al* (2022)	N/A
cvms R package	https://cran.r-project.org/web/packages/cvms/index.html<br>Olsen *et al* (2023)	N/A
Cytoscape v3.4.0	Shannon *et al* (2003)	N/A
devtools R package v2.4.3	https://cran.r-project.org/web/packages/devtools/index.html<br>Wickham *et al* (2022)	N/A
dplyr R package v1.0.8	https://cran.r-project.org/web/packages/dplyr/index.html<br>Wickham *et al* (2023b)	N/A
FLEX R package	https://github.com/csbio/FLEX_R<br>Rahman *et al* (2021)	N/A
Generalized least squares (GLS) normalization	https://github.com/kundajelab/coessentiality<br>Wainberg *et al* (2021)	N/A
ggplot2 R package v3.3.5	https://cran.r-project.org/web/packages/ggplot2/index.html<br>Wickham *et al* (2023a)	N/A
ggthemes R package v4.2.4	https://cran.r-project.org/web/packages/ggthemes/index.html<br>Arnold *et al* (2021)	N/A
gplots R package v3.1.1	https://cran.r-project.org/web/packages/gplots/index.html<br>Warnes *et al* (2022)	N/A
gridExtra R package v2.3	https://cran.r-project.org/web/packages/gridExtra/index.html<br>Auguie & Antonov (2017)	N/A

 © 2023 The Authors

Reagents and Tools table (continued)

Reagent/Resource	Source	Identifier
NumPy v1.17.4	https://numpy.org/	N/A
Pandas v0.25.1	https://zenodo.org/record/8239932	N/A
pheatmap R package v1.0.12	https://cran.r-project.org/web/packages/pheatmap/index.html	N/A
	Kolde (2019)	
Python version 3.7.3	https://www.python.org/	N/A
PyTorch v1.6.0	https://pytorch.org/	N/A
R versions 3.6.3 and 4.1.3	https://www.r-project.org/	N/A
ramify R package v0.3.3	https://cran.r-project.org/web/packages/ramify/index.html	N/A
	Greenwell (2016)	
RColorBrewer R package v1.1–3	https://cran.r-project.org/web/packages/RColorBrewer/index.html	N/A
	Neuwirth (2022)	
RcppCNPy R package v0.2.11	https://cran.r-project.org/web/packages/RcppCNPy/index.html	N/A
	Eddelbuettel & Wu (2022)	
reshape R package v0.8.9	https://cran.r-project.org/web/packages/reshape/index.html	N/A
	Wickham (2022a)	
rpca R package v0.2.3	https://cran.r-project.org/web/packages/rpca/index.html	N/A
	Candès *et al* (2011), Sykulski (2015)	
Seurat R package v4.3.0	https://cloud.r-project.org/web/packages/Seurat/index.html	N/A
	Hao *et al* (2021), Butler *et al* (2023)	
SNFtool R package v2.3.1	https://cran.r-project.org/web/packages/SNFtool/index.html (Wang *et al*, 2021)	N/A
stringi R package v1.7.6	https://cran.r-project.org/web/packages/stringi/index.html	N/A
	Gagolewski & Tartanus (2023)	
stringr R package v1.4.0	https://cran.r-project.org/web/packages/stringr/index.html	N/A
	Wickham (2022b)	
tidyr R package v1.2.0	https://cran.r-project.org/web/packages/tidyr/index.html	N/A
	Wickham *et al* (2023c)	

Methods and Protocols

Principal component analysis normalization

We applied the following steps to create PCA-normalized data from the original DepMap data -

1 As a pre-processing step, NA values were replaced with gene-wise mean CERES scores in the DepMap 20Q2 data (Achilles_gene_effect.csv) (Meyers *et al*, 2017; Dempster *et al*, 2019b; Data ref: Broad DepMap, 2020).

2 We then applied the R function prcomp (an SVD-based R implementation of PCA) to the DepMap data with scale and center parameters set to true and generated corresponding principal component (PC) outputs. The rotation variable of the PCA output corresponds to loadings of the principal components. Multiplying DepMap CERES scores with the complete rotation matrix transforms the data to a coordinate space defined by the principal components. Multiplying this resulting matrix with the transpose of the PC loadings matrix re-transforms data into the original coordinate space.

3 In our method, the original DepMap data matrix $M_{r \times c}$ is multiplied by only a subset of the principal component loadings matrix ($L_{c \times c}$) and its transpose. This creates a 'PCA-reconstructed' version of the original data matrix from the low dimensional signal-space defined by that particular subset of principal components (equation 1.1).(1.1)

$$M_{r\times c}^{reconstructed} = M_{r\times c} \times L_{c\times n} \times L_{c\times n}^{T}$$

4 The n-PC PCA-reconstruction of the original data is thus generated using the first n columns of the rotation matrix. Subtracting the PCA-reconstructed matrix from the original data matrix generates the n-PC removed PCA-normalized version of the data (equation 1.2).(1.2)

$$M_{r\times c}^{normalized} = M_{r\times c} - M_{r\times c}^{reconstructed}$$

Robust principal component analysis normalization

Robust principal component analysis (RPCA) decomposes matrix $X_{r\times c}$ into low-rank, $L_{r\times c}$, and sparse, $S_{r\times c}$, component matrices so that they satisfy equation 1.3 (Candès *et al*, 2011). RPCA is an unsupervised method, designed to optimize the values of L and S to minimize Equation 1.4, where $\|L\|_*$ is the nuclear-norm of L and $\|S\|_1$ is the l_1-norm of S. λ is a hyperparameter whose suggested value is $1 \div \sqrt{\max(r,c)}$.

© 2023 The Authors

$$X = L + S \quad (1.3)$$

$$\min \|L\|_* + \lambda\|S\|_1 \text{ s.t.} X = L + S \quad (1.4)$$

We applied the following steps to create RPCA-normalized data from the original DepMap data -

1 NA values were first replaced with gene-wise mean CERES scores in the DepMap 20Q2 data (Achilles_gene_effect.csv; Data ref: Broad DepMap, 2020).
2 We then applied the rpca function from the rpca R-package (an R implementation of RPCA, Sykulski, 2015) to the DepMap data. Variables S and L in rpca output are the RPCA-normalized data and RPCA-reconstructed data, respectively.

Autoencoder normalization

We applied the following steps to create AE-normalized data from the original DepMap data -

1 The 20Q2 DepMap data (Data ref: Broad DepMap, 2020) Achilles_gene_effect.csv, was processed in the following way to prepare data for fitting with an autoencoder model.
   a. NA values were replaced with gene-wise mean CERES scores.
   b. The dataset was row-standardized.
   c. The 0.12% of resulting z-scores below −4 or above 4 were clipped to −4 or 4, respectively.
   d. The entire dataset was min-max scaled to fall between −1 and 1.
2 A deep convolutional autoencoder was then trained on the DepMap for 1 epoch and a latent space size of LS = 1, 2, 3, 4, 5 or 10. The encoder architecture consisted of a 1D convolutional layer converting from 1 channel into 10 with a subsequent 1D max pooling layer, another 1D convolutional layer converting from 10 channels into 20 with a subsequent 1D max pooling layer, and flattening followed by a linear layer with size equal to the chosen latent space. The decoder architecture consisted of inverse operations with max unpooling, transposed convolutional layers and a final linear layer to reshape output into the original input size. All convolutional kernel sizes were set to 3 and all pooling kernel sizes were set to 2.
3 The 'reconstructed' data (decoder output) generated from the latent space is subtracted from the original DepMap to create 'normalized' data.

Onion normalization

The onion normalization method combines signals from different normalized data (that we refer to as 'layers') generated by dialing parameter values of a normalization method. It has three components – (i) normalizing gene effect scores with a dimensionality reduction method, (ii) creating similarity networks from normalized data, and finally (iii) integrating the similarity-networks into a single network.

1 Any effective dimensionality reduction method can be employed in the normalization step. The layers to be fused are produced from the same data normalized by varying a parameter of the normalization method. We created such layers by applying PCA, RPCA, or AE normalization methods to the 20Q2 DepMap data (Data ref: Broad DepMap, 2020) as described in their respective sections. For example, we created six AE-normalized layers using AE normalization with latent space sizes of 1, 2, 3, 4, 5, and 10. Similarly, we removed the first n principal components (for n = 1, 3, 5, 7, 9, 11, 13, 15, 17, or 19) and generated 10 PCA-normalized layers. For RPCA normalization, we regulated λ applying the formula $f \div \sqrt{\max(r,c)}$ for f = 0.7, 0.8, 0.9, 1, 1.1, 1.2, 1.3, r = 18,119, c = 769 and generated seven RPCA-normalized layers for integration.
2 From each normalized layer, we created a gene-level similarity network by computing Pearson correlation coefficients among the gene profiles.
3 For the network integration module of the onion method, we selected the Similarity Network Fusion (SNF) approach developed by Wang *et al* (2014a) as it outperformed baseline integration techniques we explored (Appendix Figs S14–S16). SNF is a network fusion technique based on multiview learning that enhances or diminishes network edge weights by considering the neighborhood and sparsity information of the individual networks. We converted Pearson correlation coefficients to distance metrics by subtracting them from 1 before applying a scaled exponential similarity kernel (the affinityMatrix function in the SNF R package, Wang *et al*, 2021) to generate an affinity score matrix. These affinity matrices generated from each layer of normalized data are then integrated into one network with the SNF package.

SNF has three relevant hyperparameters. The first parameter, σ, is a standard deviation regulator of the exponential similarity kernel and is used to create the affinity matrices. Another hyperparameter, k, regulates the number of neighboring vertices to be considered during calculating edge weights in the integrated network and is used both in the affinity matrix creation and the final integration stages. A third hyperparameter controls the number of iterations in the integration stage. We dialed σ = 0.1, 0.3, 0.5, 0.7 and k = 3, 5, 10, 20 in integrating AE, PCA, and RPCA normalized layers and settled on σ = 0.3, k = 5 for PCO and σ = 0.5, k = 5 for RPCO and AEO, based on how much diversity it can introduce during the evaluation process (Appendix Fig S17). We set the number of iterations to 10 for all methods. While integrating AE-normalized layers, we also included the similarity network generated from the unnormalized data as a layer, fusing a total of seven layers.

Normalization of DepMap 2022 Q4 Chronos scores

We investigated the effect of RPCA normalization, RPCO normalization as well as the GLS method (Wainberg *et al*, 2021) on the DepMap 2022 Q4 Chronos single KO effect scores (CRISPRGeneEffect.csv; Data ref: Broad DepMap, 2022).

1 Applying RPCA normalization, we generated seven normalized layers (gene × cell lines) by setting hyperparameter λ of the RPCA method to $f \div \sqrt{\max(r,c)}$, where r = 17,453, c = 1,078, and f = 0.7, 0.8, 0.9, 1, 1.1, 1.2, 1.3.
2 These normalized data were then converted into seven gene–gene profile similarity networks using Pearson Correlation Coefficients as the similarity metric.
3 These networks were integrated using the SNF method (hyperparameter σ = 0.5 and k = 5) (Wang *et al*, 2014a) to create the RPCO-normalized network.
4 The GLS pipeline (Wainberg *et al*, 2021) was applied to the same 2022 Q4 Chronos DepMap dataset using the standard settings.

 © 2023 The Authors

Functional evaluations

To evaluate normalization methods we used the CRISPR screen benchmarking package FLEX (Rahman *et al*, 2021) and the CORUM protein complex database (Giurgiu *et al*, 2019) as FLEX's gold standard to benchmark against. FLEX's evaluation is based on the idea that gene-level similarity scores, calculated from gene knock-out profiles, connote functional similarity among genes and a higher similarity score between two genes implies membership in the same protein complex. FLEX orders gene pairs from high to low similarity scores and evaluates complex membership predictions at different precision points against the CORUM standard. The precision-recall (PR) curve from FLEX depicts how many true positive (TP) gene pairs are both strongly correlated within the data and members of the same CORUM protein complex. For all PR analysis plots, we plot precision on the y-axis and the absolute number of true positives (TPs) on the x-axis. We note that this deviates from the strict definition of recall, which is a fractional value (number of TPs divided by the total number of positive examples). In this context, we prefer to plot the absolute number of TPs to provide more information about the total number of gene pairs captured at each threshold. The x-axis would simply be scaled by a single constant if recall were plotted instead.

This visualization is augmented by contribution diversity plots, which illustrate specific complexes that contribute to true positive (TP) gene pairs at various precision points on the y-axis. These plots are constructed by sliding a precision cutoff from high to low (indicated by the y-axis), and at each point, plotting a stacked bar plot across the x-axis at that point reflecting the breakdown of complex membership of the TP pairs identified at that threshold. For example, if there are X total TP gene pairs at a cutoff that results in a precision of 0.8, the diversity plot will contain a stacked bar plot centered at y = 0.80 stretching across the x-axis, where each section of the bar plot represents the fraction of pairs contributed by a specific protein complex among those X TP pairs. This stacked bar plot is recomputed at each precision point to reflect the set of TP pairs satisfying the corresponding cutoff. As a result, a visually larger area from a complex denotes more TP contribution from that complex across the y-axis. In all diversity plots across the manuscript, the top 10 contributing complexes are shown in red or blue shades and all other complexes contributing at a lower frequency are represented in gray.

Another evaluation metric in FLEX is the per-complex area under the PR curve (AUPRC) value. In calculating AUPRC for a complex, gene pairs belonging to that complex are considered as positive examples whereas gene pairs from other complexes are set as negative examples. A higher per-complex AUPRC indicates more gene pairs associated with that complex have been identified based on their similarity scores. Conversely, a lower per-complex AUPRC means that scores for the within-complex genes are poorly correlated compared to between-complex gene pairs.

FLEX also facilitates removing specific annotated gene pairs from the evaluation process so that they contribute to neither true positives nor false-positives. To evaluate the influence of mitochondrial complexes in the DepMap data, we compiled 1,266 mitochondrial genes from three sources to remove from our analysis. A total of 1,136 genes were collected from the Human MitoCarta3.0, an inventory of human mitochondrial proteins and pathways by the Broad Institute (Rath *et al*, 2021). All genes from the KEGG OXIDATIVE PHOSPHORYLATION and the REACTOME RESPIRATORY ELECTRON TRANSPORT pathways were included in the list. 436 genes were also assembled by an expert based on information from pathways and CORUM complexes, and the union of these lists formed a reference list of mitochondrial-associated genes. To modify FLEX analyses according to this list and better examine non-mitochondrial signal within the DepMap, gene pairs were excluded from FLEX analyses where both genes are contained in the mitochondrial gene list. Gene pairs that contain only one or no mitochondrial genes are not removed.

Network analysis

Networks were constructed from the original 20Q2 DepMap and the RPCO-normalized DepMap datasets by taking the top five, ten, or fifteen-thousand edges based on the strength of Pearson correlations across each respective dataset (Data ref: Broad DepMap, 2020). Network layouts were performed with the yFiles organic layout algorithm in Cytoscape version 3.7.2 (Shannon *et al*, 2003). All connected components within each network were treated as separate clusters and analyzed for enrichment. Enrichments tests were performed with hypergeometric tests using the clusterProfiler R package version 3.16.1 by Wu *et al* (2021) against human Gene Ontology-biological process and MSigDB C2-curated pathway annotations and a background set of all genes in the given network at a Benjamini–Hochberg FDR of 0.2.

Analysis on cell line similarity network

We analyzed the effect of RPCO normalization on the DepMap 20Q2 (Data ref: Broad DepMap, 2020) cell line similarity network. To create a RPCO-normalized network the following steps were taken:

1 We applied RPCA (Candès *et al*, 2011) to DepMap 20Q2 cell line profiles (Ceres scores across genes) and generated seven normalized layers (cell lines × genes) by setting hyperparameter λ of the RPCA method to $f \div \sqrt{\max(r,c)}$, where $r = 769$, $c = 18{,}119$, and $f = 0.7, 0.8, 0.9, 1, 1.1, 1.2, 1.3$.
2 Seven cell line similarity networks were created from the normalized data using Pearson Correlation Coefficient as a similarity metric
3 The seven networks were integrated using SNF (Wang *et al*, 2014a) ($\sigma = 0.5$, $k = 5$) to generate a RPCO-normalized network.

For the tissue-lineage prediction task the following methods were applied,

1 A tissue label was assigned to a cell line using k-nearest neighbor with majority voting. The highest similarity score neighbor label was assigned in case of a tie.
2 The overall precision, recall and F1 scores were calculated using weighted mean of scores from individual classes. The true tissue labels for cell lines are derived from the sample_info.csv file provided with DepMap 20Q2.
3 The baseline prediction scores were calculated by a random classifier and taking the average of 100 iterations.

© 2023 The Authors

Analysis on gene-expression data

To investigate the RPCO normalization on scRNA-seq gene expression data, we applied the following steps:

1. The scRNA-seq gene expression dataset (5k_pbmc_v3_filtered_feature_bc_matrix.tar) was downloaded from 10xGENOMICS (Data ref: 10x Genomics, 2019). The dataset was generated from Peripheral blood mononuclear cells (PBMCs) using Chromium and Cell Ranger.
2. We applied the Seurat R package (Hao *et al*, 2021; Butler *et al*, 2023) to filter the dataset.
   a. We removed genes for which the number of cells with non-zero values is smaller than or equal to 50.
   b. We also filtered out cells for which the number of unique genes detected in each cell is $\leq$ 100 and $\geq$ 4,500.
   c. We only included cells for which the percentage of reads that map to the mitochondrial genome is lower than 7.
   d. The final matrix contains 12,410 genes and 1,195 cells, around 20% of which is non-zero. We log-normalized the data using Seurat function NormalizeData with default parameters.
3. We applied RPCA to the pre-processed scRNA-seq data and generated seven RPCA-normalized layers by setting hyperparameter lambda to $f \div \sqrt{\max(r,c)}$, where $r = 12{,}000$, $c = 1{,}200$, and $f = 0.7, 0.8, 0.9, 1, 1.1, 1.2, 1.3$.
4. Seven gene–gene similarity networks were generated from the normalized data using Pearson Correlation Coefficients as the similarity metric.
5. The networks were integrated by taking the maximum weight for each gene pair across the seven networks.
6. To demonstrate the dominance of cytoplasmic ribosomal gene pairs in the analysis results (Appendix Fig S13B right), we removed 81 Ribosome (cytoplasmic) complex-associated genes during the FLEX evaluation process.

Data availability

The computer code and data produced in this study are available in the following databases:

- Code to reproduce the main figures: GitHub (https://github.com/ArshiaZHassan/ONION_git).
- Code for autoencoder-normalization: GitHub (https://github.com/csbio/ae-norm).
- Data to reproduce the main figures and associated outputs: Zenodo (https://zenodo.org/record/7671685#.Y_gi9nbMK5c).

Expanded View for this article is available online.

Acknowledgements

This research was funded by grants from the National Science Foundation (MCB 1818293), the National Institutes of Health (R01HG005084, R01HG005853). H.N.W was partially supported by the Doctoral Dissertation Fellowship from the U. of Minnesota and a National Institutes of Health (NIH) Biotechnology Training Grant (T32GM008347). M.B. was partially supported by the German Research Foundation DFG (Bi 2086/1–1). Open Access funding enabled and organized by Projekt DEAL.

Author contributions

Arshia Zernab Hassan: Conceptualization; data curation; software; formal analysis; investigation; visualization; writing – original draft; writing – review and editing. **Henry N Ward:** Conceptualization; data curation; software; formal analysis; investigation; visualization; writing – original draft; writing – review and editing. **Mahfuzur Rahman:** Resources; software; investigation; writing – review and editing. **Maximilian Billmann:** Resources; investigation; writing – review and editing. **Yoonkyu Lee:** Resources; investigation; writing – review and editing. **Chad L Myers:** Conceptualization; supervision; funding acquisition; investigation; project administration; writing – review and editing.

Disclosure and competing interests statement

The authors declare that they have no conflict of interest.

References

10x Genomics (2019) 5k peripheral blood mononuclear cells (PBMCs) from a healthy donor (v3 chemistry), single cell gene expression dataset by Cell Ranger 3.0.2. (https://support.10xgenomics.com/single-cell-gene-expression/datasets/3.0.2/5k_pbmc_v3). [DATASET]

Arnold JB, Daroczi G, Werth B, Weitzner B, Kunst J, Auguie B, Rudis B, Wickham H, Talbot J, London J (2021) ggthemes: Extra Themes, Scales and Geoms for 'ggplot2'. R package v4.2.4.

Auguie B, Antonov A (2017) gridExtra: miscellaneous functions for 'Grid' graphics. R package v2.3.

Baryshnikova A, Costanzo M, Kim Y, Ding H, Koh J, Toufighi K, Youn J-Y, Ou J, San Luis B-J, Bandyopadhyay S *et al* (2010) Quantitative analysis of fitness and genetic interactions in yeast on a genome scale. *Nat Methods* 7: 1017–1024

Behan FM, Iorio F, Picco G, Gonçalves E, Beaver CM, Migliardi G, Santos R, Rao Y, Sassi F, Pinnelli M *et al* (2019) Prioritization of cancer therapeutic targets using CRISPR–Cas9 screens. *Nature* 568: 511–516

Boyle EA, Pritchard JK, Greenleaf WJ (2018) High-resolution mapping of cancer cell networks using co-functional interactions. *Mol Syst Biol* 14: e8594

Broad DepMap (2018) DepMap Achilles 18Q3 public. (https://figshare.com/articles/DepMap_Achilles_18Q3_public/6931364/1). [DATASET]

Broad DepMap (2019a) DepMap 19Q3 Public. (https://figshare.com/articles/DepMap_19Q3_Public/9201770/2). [DATASET]

Broad DepMap (2019b) DepMap 19Q2 Public. (https://figshare.com/articles/DepMap_19Q2_Public/8061398/1). [DATASET]

Broad DepMap (2020) DepMap 20Q2 Public. (https://figshare.com/articles/DepMap_20Q2_Public/12280541/4). [DATASET]

Broad DepMap (2022) DepMap 22Q4 Public. (https://figshare.com/articles/dataset/DepMap_22Q4_Public/21637199/2). [DATASET]

Buphamalai P, Kokotovic T, Nagy V, Menche J (2021) Network analysis reveals rare disease signatures across multiple levels of biological organization. *Nat Commun* 12: 6306

Butler A, Choudhary S, Darby C, Farrell J, Hafemeister C, Hao Y, Hartman A, Hoffman P, Jain J, Kowalski M *et al* (2023) Seurat: tools for single cell genomics.

Candès EJ, Li X, Ma Y, Wright J (2011) Robust principal component analysis? *J ACM* 58: 11:1 11:37

Chiu Y-C, Zheng S, Wang L-J, Iskra BS, Rao MK, Houghton PJ, Huang Y, Chen Y (2021) Predicting and characterizing a cancer dependency map of tumors with deep learning. *Science. Advances* 7: eabh1275

 © 2023 The Authors

Costanzo M, VanderSluis B, Koch EN, Baryshnikova A, Pons C, Tan G, Wang W, Usaj M, Hanchard J, Lee SD *et al* (2016) A global genetic interaction network maps a wiring diagram of cellular function. *Science* 353: aaf1420

Davis TL, Newell P, Day A (2023) argparse: command line optional and positional argument parser. R package v2.0.3.

Dempster JM, Pacini C, Pantel S, Behan FM, Green T, Krill-Burger J, Beaver CM, Younger ST, Zhivich V, Najgebauer H *et al* (2019a) Agreement between two large pan-cancer CRISPR-Cas9 gene dependency data sets. *Nat Commun* 10: 5817

Dempster JM, Rossen J, Kazachkova M, Pan J, Kugener G, Root DE, Tsherniak A (2019b) Extracting biological insights from the Project Achilles Genome-Scale CRISPR screens in cancer cell lines. *bioRxiv* https://doi.org/10.1101/720243 [PREPRINT]

Dempster JM, Boyle I, Vazquez F, Root D, Boehm JS, Hahn WC, Tsherniak A, McFarland JM (2021) Chronos: a CRISPR cell population dynamics model. *bioRxiv* https://doi.org/10.1101/2021.02.25.432728 [PREPRINT]

Dharia NV, Kugener G, Guenther LM, Malone CF, Durbin AD, Hong AL, Howard TP, Bandopadhayay P, Wechsler CS, Fung I *et al* (2021) A first-generation pediatric cancer dependency map. *Nat Genet* 53: 529–538

Ding J, Condon A, Shah SP (2018) Interpretable dimensionality reduction of single cell transcriptome data with deep generative models. *Nat Commun* 9: 2002

Eddelbuettel D, Wu W (2022) RcppCNPy: read-write support for 'NumPy' files via 'Rcpp'. R package v0.2.11.

Gagolewski M, Tartanus B (2023) stringi: fast and portable character string processing facilities. R package v1.7.6.

Gheorghe V, Hart T (2022) Optimal construction of a functional interaction network from pooled library CRISPR fitness screens. *BMC Bioinformatics* 23: 510

Giurgiu M, Reinhard J, Brauner B, Dunger-Kaltenbach I, Fobo G, Frishman G, Montrone C, Ruepp A (2019) CORUM: the comprehensive resource of mammalian protein complexes—2019. *Nucleic Acids Res* 47: D559–D563

Greenwell B (2016) ramify: additional matrix functionality. R package v0.3.3.

Hao Y, Hao S, Andersen-Nissen E, Mauck WM, Zheng S, Butler A, Lee MJ, Wilk AJ, Darby C, Zager M *et al* (2021) Integrated analysis of multimodal single-cell data. *Cell* 184: 3573–3587.e29

Hinton GE, Salakhutdinov RR (2006) Reducing the dimensionality of data with neural networks. *Science* 313: 504–507

Kim E, Dede M, Lenoir WF, Wang G, Srinivasan S, Colic M, Hart T (2019) A network of human functional gene interactions from knockout fitness screens in cancer cells. *Life Sci Alliance* 2: e201800278

Kingma DP, Welling M (2022) Auto-encoding variational Bayes. *bioRxiv* https://doi.org/10.48550/arXiv.1312.6114 [PREPRINT]

Kolde R (2019) pheatmap: pretty heatmaps. R package v1.0.12.

Lopez R, Regier J, Cole MB, Jordan MI, Yosef N (2018) Deep generative modeling for single-cell transcriptomics. *Nat Methods* 15: 1053–1058

Lotfollahi M, Wolf FA, Theis FJ (2019) scGen predicts single-cell perturbation responses. *Nat Methods* 16: 715–721

Ma J, Fong SH, Luo Y, Bakkenist CJ, Shen JP, Mourragui S, Wessels LFA, Hafner M, Sharan R, Peng J *et al* (2021) Few-shot learning creates predictive models of drug response that translate from high-throughput screens to individual patients. *Nat Cancer* 2: 233–244

Meyers RM, Bryan JG, McFarland JM, Weir BA, Sizemore AE, Xu H, Dharia NV, Montgomery PG, Cowley GS, Pantel S *et al* (2017) Computational correction of copy number effect improves specificity of CRISPR–Cas9 essentiality screens in cancer cells. *Nat Genet* 49: 1779–1784

Neuwirth E (2022) RColorBrewer: ColorBrewer palettes. R package v1.1-3.

Olsen LR, Zachariae HB, Patil I, Lüdecke D (2023) cvms: cross-validation for model selection. R package v1.6.0.

Pacini C, Dempster JM, Boyle I, Gonçalves E, Najgebauer H, Karakoc E, van der Meer D, Barthorpe A, Lightfoot H, Jaaks P *et al* (2021) Integrated cross-study datasets of genetic dependencies in cancer. *Nat Commun* 12: 1661

Pan J, Kwon JJ, Talamas JA, Borah AA, Vazquez F, Boehm JS, Tsherniak A, Zitnik M, McFarland JM, Hahn WC (2022) Sparse dictionary learning recovers pleiotropy from human cell fitness screens. *Cell Systems* 13: 286–303.e10

Pan J, Meyers RM, Michel BC, Mashtalir N, Sizemore AE, Wells JN, Cassel SH, Vazquez F, Weir BA, Hahn WC *et al* (2018) Interrogation of mammalian protein complex structure, function, and membership using genome-scale fitness screens. *Cell Systems* 6: 555–568.e7

Rahman M, Billmann M, Costanzo M, Aregger M, Tong AHY, Chan K, Ward HN, Brown KR, Andrews BJ, Boone C *et al* (2021) A method for benchmarking genetic screens reveals a predominant mitochondrial bias. *Mol Syst Biol* 17: e10013

Rath S, Sharma R, Gupta R, Ast T, Chan C, Durham TJ, Goodman RP, Grabarek Z, Haas ME, Hung WHW *et al* (2021) MitoCarta3.0: an updated mitochondrial proteome now with sub-organelle localization and pathway annotations. *Nucleic Acids Res* 49: D1541–D1547

Shalem O, Sanjana NE, Hartenian E, Shi X, Scott DA, Mikkelsen TS, Heckl D, Ebert BL, Root DE, Doench JG *et al* (2014) Genome-scale CRISPR-Cas9 knockout screening in human cells. *Science* 343: 84–87

Shannon P, Markiel A, Ozier O, Baliga NS, Wang JT, Ramage D, Amin N, Schwikowski B, Ideker T (2003) Cytoscape: a software environment for integrated models of biomolecular interaction networks. *Genome Res* 13: 2498–2504

Shimada K, Bachman JA, Muhlich JL, Mitchison TJ (2021) shinyDepMap, a tool to identify targetable cancer genes and their functional connections from Cancer Dependency Map data. *eLife* 10: e57116

Sun S, Zhu J, Ma Y, Zhou X (2019) Accuracy, robustness and scalability of dimensionality reduction methods for single-cell RNA-seq analysis. *Genome Biol* 20: 269

Sykulski M (2015) rpca: RobustPCA: decompose a matrix into low-rank and sparse components. R package v0.2.3.

Tsherniak A, Vazquez F, Montgomery PG, Weir BA, Kryukov G, Cowley GS, Gill S, Harrington WF, Pantel S, Krill-Burger JM *et al* (2017) Defining a cancer dependency map. *Cell* 170: 564–576.e16

Wainberg M, Kamber RA, Balsubramani A, Meyers RM, Sinnott-Armstrong N, Hornburg D, Jiang L, Chan J, Jian R, Gu M *et al* (2021) A genome-wide atlas of co-essential modules assigns function to uncharacterized genes. *Nat Genet* 53: 638–649

Wang B, Mezlini AM, Demir F, Fiume M, Tu Z, Brudno M, Haibe-Kains B, Goldenberg A (2014a) Similarity network fusion for aggregating data types on a genomic scale. *Nat Methods* 11: 333–337

Wang T, Wei JJ, Sabatini DM, Lander ES (2014b) Genetic screens in human cells using the CRISPR-Cas9 system. *Science* 343: 80–84

Wang B, Mezlini A, Demir F, Fiume M, Tu Z, Brudno M, Haibe-Kains B, Goldenberg A (2021) SNFtool: similarity network fusion. R package v2.3.1.

Warnes GR, Bolker B, Bonebakker L, Gentleman R, Huber W, Liaw A, Lumley T, Maechler M, Magnusson A, Moeller S *et al* (2022) gplots: various R programming tools for plotting data. R package v3.1.1.

Way GP, Greene CS (2017) Extracting a biologically relevant latent space from cancer transcriptomes with variational autoencoders. *Pac Symp Biocomput* 23: 80–91

© 2023 The Authors

Wickham H (2022a) reshape: flexibly reshape data. R package v0.8.9.
Wickham H (2022b) stringr: simple, consistent wrappers for common string operations. R package v1.4.0.
Wickham H, Hester J, Chang W, Bryan J (2022) devtools: tools to make developing R packages easier. R package v2.4.3.
Wickham H, Chang W, Henry L, Pedersen TL, Takahashi K, Wilke C, Woo K, Yutani H, Dunnington D (2023a) ggplot2: create elegant data visualisations using the grammar of graphics. R package v3.3.5.
Wickham H, François R, Henry L, Müller K, Vaughan D (2023b) dplyr: a grammar of data manipulation. R package v1.0.8.
Wickham H, Vaughan D, Girlich M, Ushey K (2023c) tidyr: tidy messy data. R package v.1.2.0.
Wijffels J, Okazaki N, Quark C, Jenkins B, Nocedal J, Long J (2022) crfsuite: conditional random fields for labelling sequential data in natural language processing. R package v.0.4.1.
Wold S, Esbensen K, Geladi P (1987) Principal component analysis. *Chemometr Intell Lab Syst* 2: 37–52
Wu T, Hu E, Xu S, Chen M, Guo P, Dai Z, Feng T, Zhou L, Tang W, Zhan L *et al* (2021) clusterProfiler 4.0: a universal enrichment tool for interpreting omics data. *Innovation* 2: 100141
Zheng GXY, Terry JM, Belgrader P, Ryvkin P, Bent ZW, Wilson R, Ziraldo SB, Wheeler TD, McDermott GP, Zhu J *et al* (2017) Massively parallel digital transcriptional profiling of single cells. *Nat Commun* 8: 14049

License: This is an open access article under the terms of the Creative Commons Attribution License, which permits use, distribution and reproduction in any medium, provided the original work is properly cited.

 © 2023 The Authors

Expanded View Figures

Figure EV1. CORUM complex z-scores of AUPRC values for PCA normalization.

AUPRC values per complex (rows) for the un-normalized DepMap 20Q2 data (Data ref: Broad DepMap, 2020) (far left column) are compared to PCA-normalized DepMap data with the first 1, 3, 5, 7, 9, 11, 13, 15, 17, or 19 PCs removed. AUPRC values have been z-score-normalized per row for visualization purposes.

© 2023 The Authors

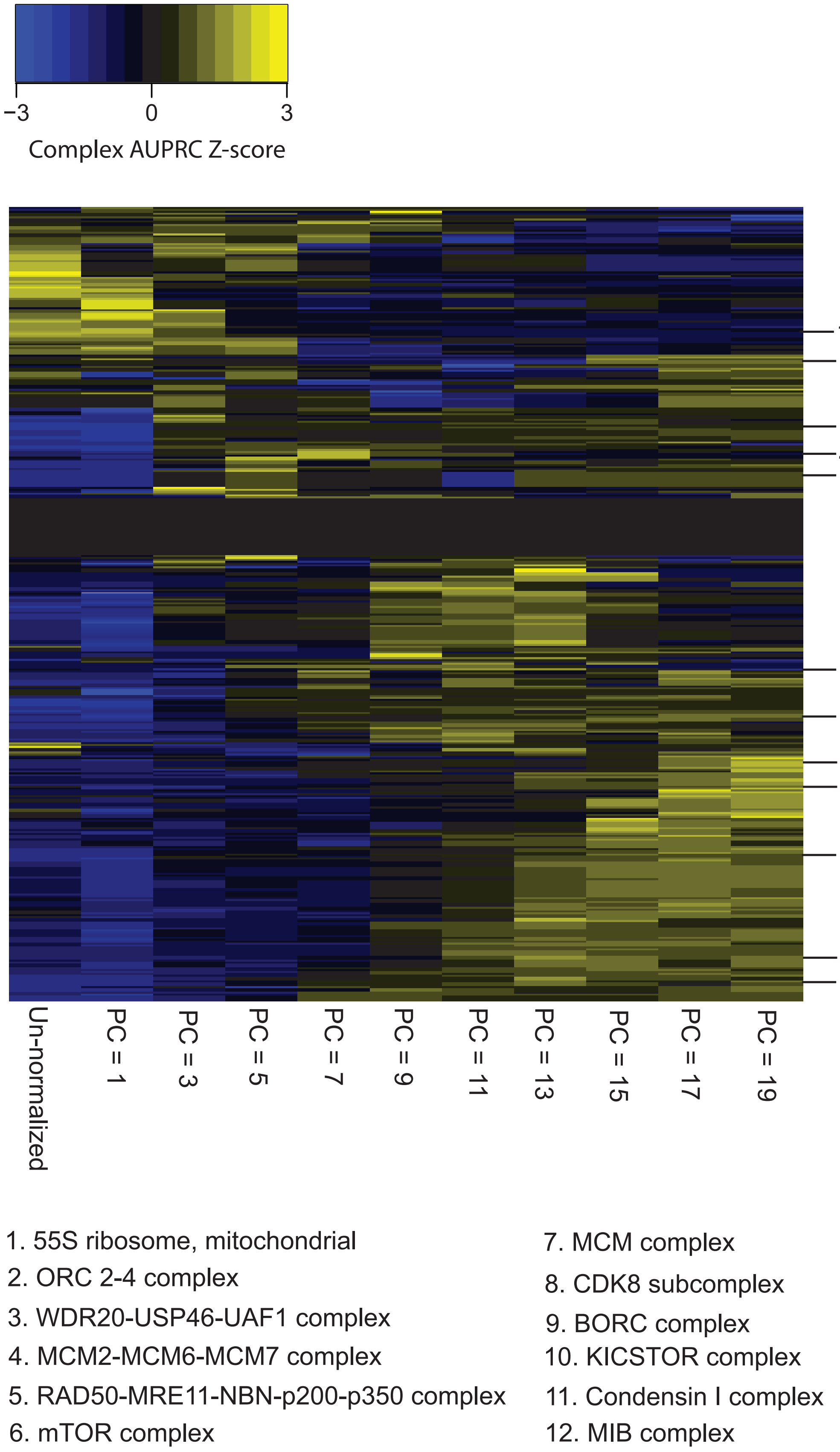

Figure EV1.

© 2023 The Authors

Figure EV2. CORUM complex z-scores of AUPRC values for robust PCA normalization.

AUPRC values per complex (rows) for the un-normalized DepMap 20Q2 data (Data ref: Broad DepMap, 2020) (far left column) are compared to robust RPCA-normalized DepMap data for $\lambda \approx$ 0.0049, 0.0056, 0.0063, 0.007, 0.0077, 0.0084, 0.0091. AUPRC values have been z-score-normalized per row for visualization purposes.

© 2023 The Authors

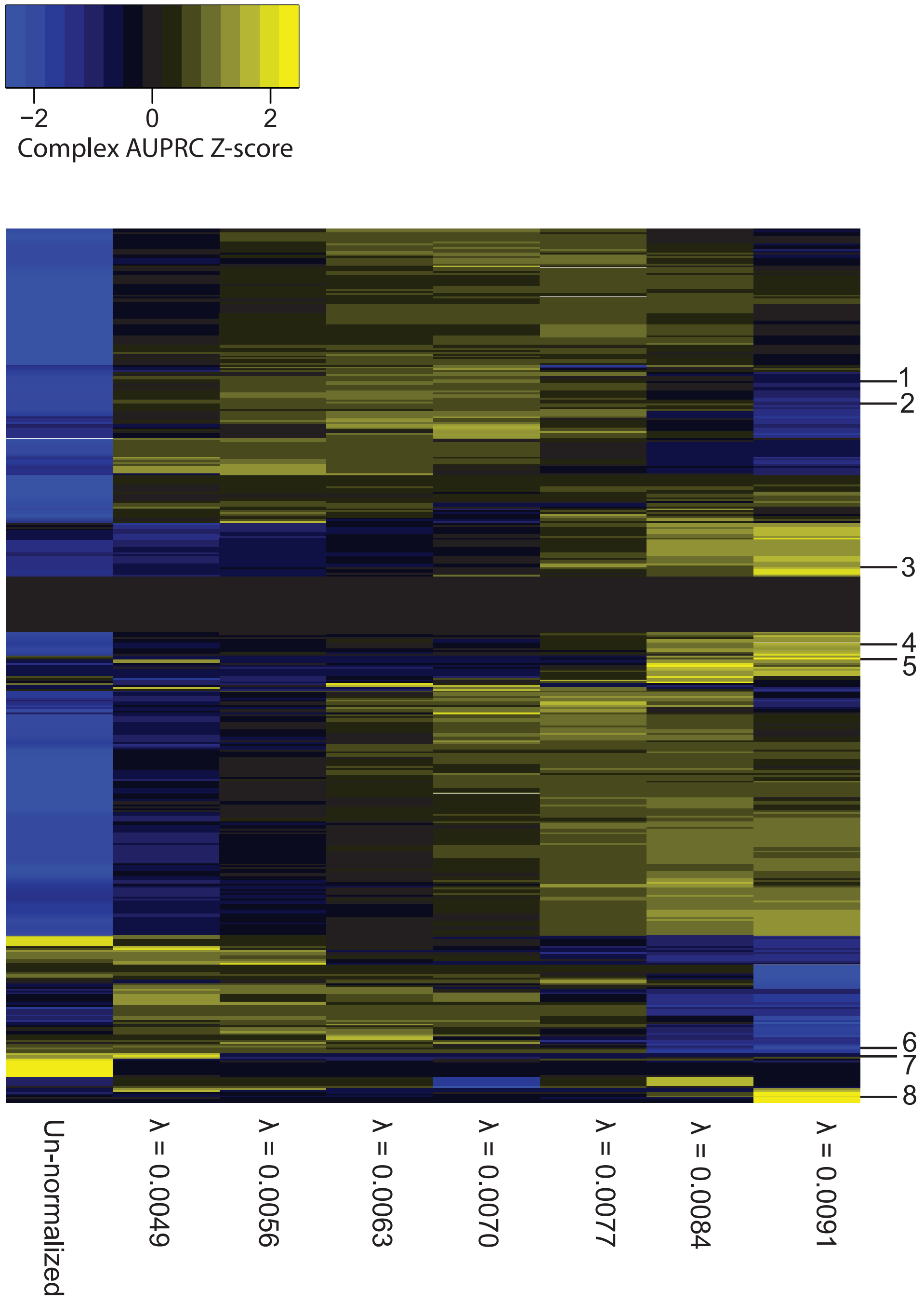

1. ESCRT-II complex
2. ORC 2-4 complex
3. WICH complex
4. RAD17-RFC complex
5. HUSH complex
6. 55S ribosome, mitochondrial
7. hMediator complex
8. KEOPS complex

Figure EV2.

© 2023 The Authors

Figure EV3. CORUM complex z-scores of AUPRC values for autoencoder normalization.

AUPRC values per complex (rows) for the un-normalized DepMap 20Q2 data (Data ref: Broad DepMap, 2020) (far left column) compared to AE-normalized DepMap data with latent space size (LS) = 1, 2, 3, 4, 5, 10. AUPRC values have been z-score-normalized per row for visualization purposes.

© 2023 The Authors

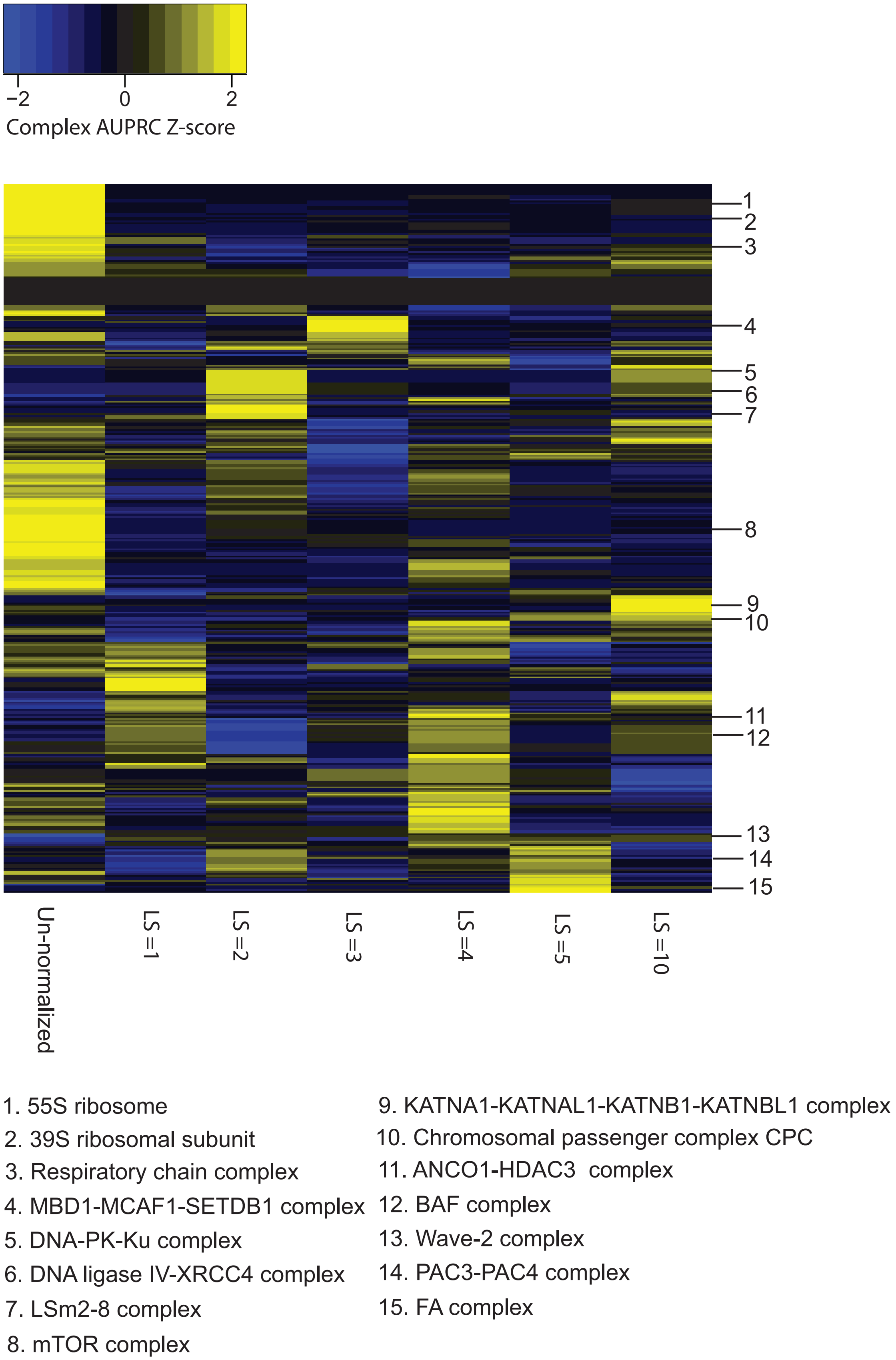

Figure EV3.

© 2023 The Authors

Figure EV4. *K*-nearest neighbor classifier tissue-lineage prediction results comparing un-normalized and RPCO-normalized DepMap 20Q2 (Data ref: Broad DepMap, 2020) cell line similarity networks.

A Overall F1, Precision, and Recall scores (weighted mean across classes) across different values of K (x-axis). Dashed line represents the scores from a baseline random classifier.

B Class-level (tissue-lineage) F1, Precision, and Recall scores for $K = 5$ where x-axis depicts tissue lineage.

© 2023 The Authors

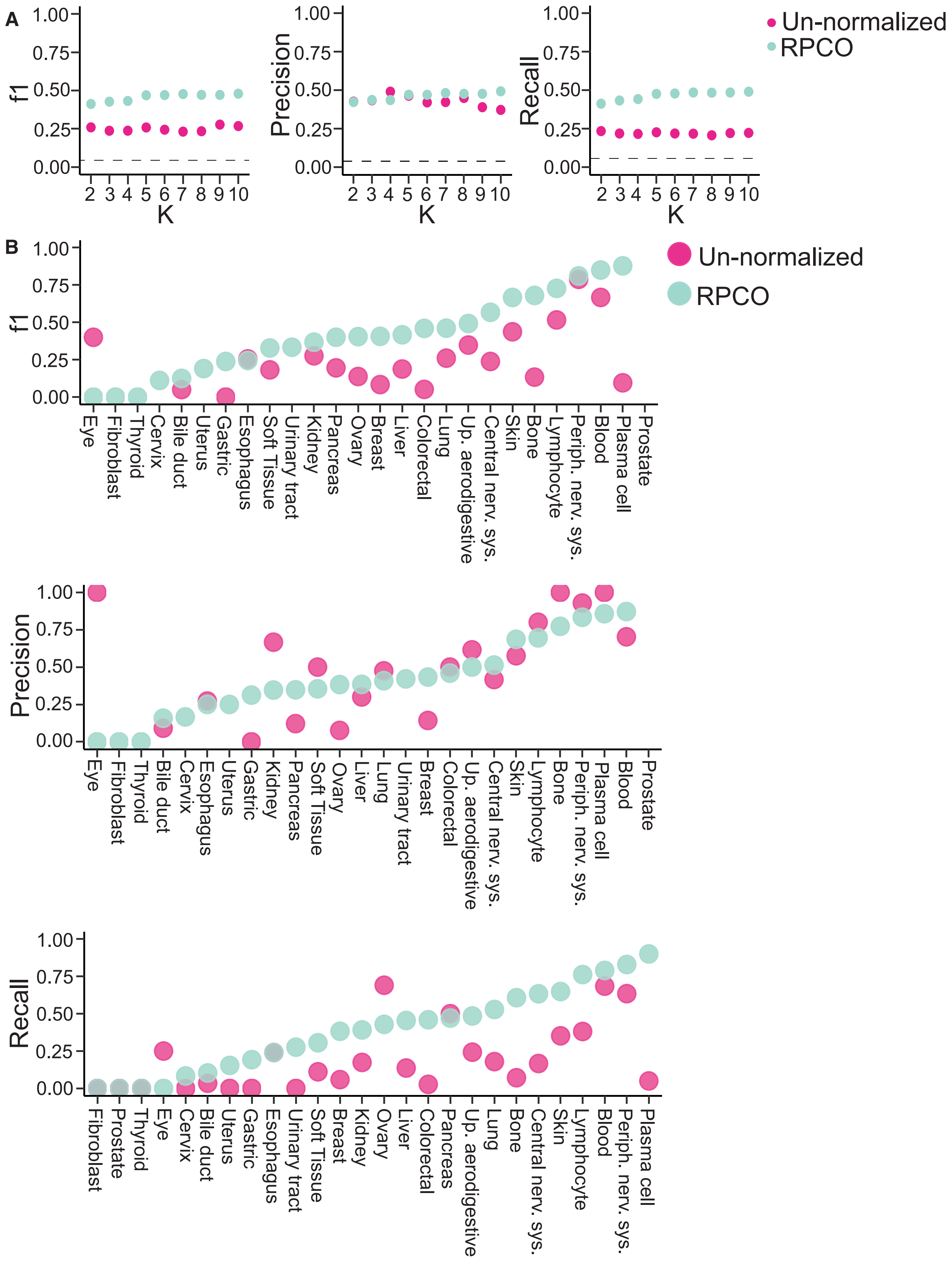

Figure EV4.

© 2023 The Authors

Figure EV5. Confusion matrix from *K*-nearest neighbor classifier (K = 5) showing prediction results for each class (tissue lineage) of cell lines from un-normalized and RPCO-normalized DepMap 20Q2 (Data ref: Broad DepMap, 2020) cell line similarity networks.

X- and y-axes are class labels. The top row depicts the true number of cell lines in each class, and the right-most column is the number of predicted cell lines for each class. The diagonal represents true positives. Each row represents false positives, and each column represents false negatives sans the diagonal entry.

© 2023 The Authors

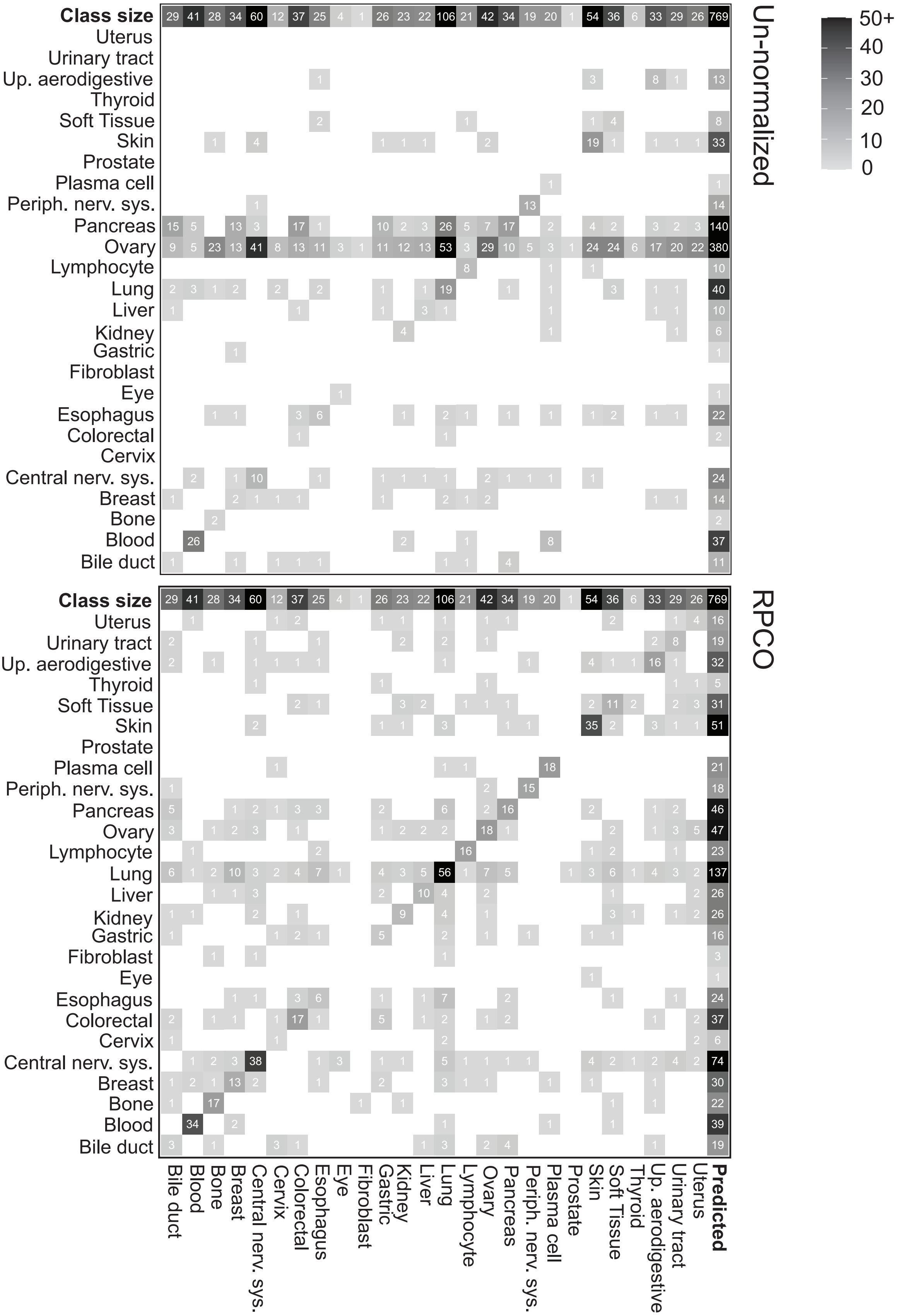

Figure EV5.

© 2023 The Authors
