# Supplementary material for: Dimensionality reduction methods for extracting functional networks from large‐scale CRISPR screens
Source: Mol Syst Biol. 2023 Sep 26;19(11):e11657. doi: 10.15252/msb.202311657 (PMC10632734; doi:10.15252/msb.202311657)
Supplement: Supplementary file 4 [file MSB-19-e11657-s003.pdf]

molecular systems biology

Dimensionality reduction methods for extracting functional networks from large-scale CRISPR screens

Arshia Zernab Hassan, Henry Ward, Mahfuzur Rahman, Maximilian Billmann, Yoonkyu Lee, and Chad Myers
DOI: 10.15252/msb.202311657

Corresponding author(s): Chad Myers (cmyers@cs.umn.edu)

Review Timeline:

Submission Date:	19th Mar 23
Editorial Decision:	27th Apr 23
Revision Received:	26th Jul 23
Editorial Decision:	8th Aug 23
Revision Received:	28th Aug 23
Accepted:	5th Sep 23

Editor: Maria Polychronidou

Transaction Report:

(Note: With the exception of the correction of typographical or spelling errors that could be a source of ambiguity, letters and reports are not edited. Depending on transfer agreements, referee reports obtained elsewhere may or may not be included in this compilation. Referee reports are anonymous unless the Referee chooses to sign their reports.)

27th Apr 2023

Manuscript Number: MSB-2023-11657
Title: Dimensionality reduction methods for extracting functional networks from large-scale CRISPR screens

Dear Chad,

Thank you again for submitting your work to Molecular Systems Biology. We have now heard back from the two reviewers who agreed to evaluate your study. As you will see below, the reviewers appreciate that the presented approach addresses a relevant problem. However, they raise a series of concerns, which we would ask you to address in a major revision.

I think that the recommendations of the reviewers are rather clear and I therefore do not see the need to repeat the comments listed below. Some of the more fundamental points raised refers to the need to clarify the novel contributions of the study and to better support the advantages over existing approaches. All issues raised would need to be satisfactorily addressed. Please let me know in case you would like to discuss in further detail any of the issues raised, I would be happy to schedule a call.

On a more editorial level, we would ask you to address the following points:

- Please provide a .doc version of the manuscript text (including legends for main figures) and individual production quality figure files for the main Figures (one file per figure).

- We have replaced Supplementary Information by the Expanded View (EV format). In this case, all additional figures can be included in a PDF called Appendix. Appendix figures should be labeled and called out as: "Appendix Figure S1, Appendix Figure S2..." etc. Each legend should be below the corresponding Figure in the Appendix. Please include a Table of Contents in the beginning of the Appendix. For detailed instructions regarding expanded view please refer to our Author Guidelines: .

- Please provide a "standfirst text" summarizing the study in one or two sentences (approximately 250 characters), three to four "bullet points" highlighting the main findings and a "synopsis image" (550px width and max 400px height, jpeg format) to highlight the paper on our homepage.

- All Materials and Methods need to be described in the main text. We would ask you to use 'Structured Methods', our new Materials and Methods format, which is mandatory for Methods and Articles with a strong methodological focus. According to this format, the Material and Methods section should include a Reagents and Tools Table (listing key reagents, experimental models, software and relevant equipment and including their sources and relevant identifiers) followed by a Methods and Protocols section in which we encourage the authors to describe their methods using a step-by-step protocol format with bullet points, to facilitate the adoption of the methodologies across labs. More information on how to adhere to this format as well as downloadable templates (.doc or .xls) for the Reagents and Tools Table can be found in our author guidelines: . An example of a Method paper with Structured Methods can be found here: .

- Please include a Data availability section describing how the data and code have been made available. This section needs to be formatted according to the example below:
The datasets and computer code produced in this study are available in the following databases:
- Chip-Seq data: Gene Expression Omnibus GSE46748 (https://www.ncbi.nlm.nih.gov/geo/query/acc.cgi?acc=GSE46748)
- Modeling computer scripts: GitHub (https://github.com/SysBioChalmers/GECKO/releases/tag/v1.0)
- [data type]: [full name of the resource] [accession number/identifier] ([doi or URL or identifiers.org/DATABASE:ACCESSION])

- For data quantification: please specify the name of the statistical test used to generate error bars and P values, the number (n) of independent experiments (specify technical or biological replicates) underlying each data point and the test used to calculate p-values in each figure legend. The figure legends should contain a basic description of n, P and the test applied. Graphs must include a description of the bars and the error bars (s.d., s.e.m.).

- Please include a "Disclosure & Competing Interests Statement" in the main text.

- Molecular Systems Biology supports formal data citations in the Reference list, to cite previously published datasets. In addition to citing the original papers that reported the data, we encourage you to also cite the relevant datasets directly in the Reference list. In the text, references to datasets are included as "Data ref: Smith et al, 2001" or "Data ref: NCBI Sequence Read Archive PRJNA342805, 2017". In the Reference list, data citations are very similar to normal literature references but must be labeled with "[DATASET]" at the end of the reference. For detailed instructions please refer to our Author Guidelines .

- When you resubmit your manuscript, please download our CHECKLIST (https://bit.ly/EMBOPressAuthorChecklist) and include the completed form in your submission.

Please note that the Author Checklist will be published alongside the paper as part of the transparent process (https://www.embopress.org/page/journal/17444292/authorguide#transparentprocess).

If you feel you can satisfactorily deal with these points and those listed by the referees, you may wish to submit a revised version of your manuscript. Please attach a covering letter giving details of the way in which you have handled each of the points raised by the referees. A revised manuscript will be once again subject to review and you probably understand that we can give you no guarantee at this stage that the eventual outcome will be favorable.

Kind regards,

Maria

Maria Polychronidou, PhD
Senior Editor
Molecular Systems Biology

We realize that it is difficult to revise to a specific deadline. In the interest of protecting the conceptual advance provided by the work, we recommend a revision within 3 months (26th Jul 2023). Please discuss the revision progress ahead of this time with the editor if you require more time to complete the revisions. Use the link below to submit your revision:

https://msb.msubmit.net/cgi-bin/main.plex

IMPORTANT: When you send your revision, we will require the following items:
1. the manuscript text in LaTeX, RTF or MS Word format
2. a letter with a detailed description of the changes made in response to the referees. Please specify clearly the exact places in the text (pages and paragraphs) where each change has been made in response to each specific comment given
3. three to four 'bullet points' highlighting the main findings of your study
4. a short 'blurb' text summarizing in two sentences the study (max. 250 characters)
5. a 'thumbnail image' (550px width and max 400px height, Illustrator, PowerPoint or jpeg format), which can be used as 'visual title' for the synopsis section of your paper.
6. Please include an author contributions statement after the Acknowledgements section (see https://www.embopress.org/page/journal/17444292/authorguide)
7. Please complete the CHECKLIST available at (https://bit.ly/EMBOPressAuthorChecklist).
Please note that the Author Checklist will be published alongside the paper as part of the transparent process (https://www.embopress.org/page/journal/17444292/authorguide#transparentprocess).
8. When assembling figures, please refer to our figure preparation guideline in order to ensure proper formatting and readability in print as well as on screen:
https://bit.ly/EMBOPressFigurePreparationGuideline
See also figure legend guidelines: https://www.embopress.org/page/journal/17444292/authorguide#figureformat
9. Please note that corresponding authors are required to supply an ORCID ID for their name upon submission of a revised manuscript (EMBO Press signed a joint statement to encourage ORCID adoption).
(https://www.embopress.org/page/journal/17444292/authorguide#editorialprocess)
Currently, our records indicate that the ORCID for your account is 0000-0002-1026-5972.

Please click the link below to modify this ORCID:
Link Not Available

10. At EMBO Press we ask authors to provide source data for the main manuscript figures. Our source data coordinator will contact you to discuss which figure panels we would need source data for and will also provide you with helpful tips on how to upload and organize the files.

The system will prompt you to fill in your funding and payment information. This will allow Wiley to send you a quote for the article processing charge (APC) in case of acceptance. This quote takes into account any reduction or fee waivers that you may be eligible for. Authors do not need to pay any fees before their manuscript is accepted and transferred to the publisher.

EMBO Press participates in many Publish and Read agreements that allow authors to publish Open Access with reduced/no publication charges. Check your eligibility: https://authorservices.wiley.com/author-resources/Journal-Authors/open-access/affiliation-policies-payments/index.html

As a matter of course, please make sure that you have correctly followed the instructions for authors as given on the submission website.

*** PLEASE NOTE *** As part of the EMBO Press transparent editorial process initiative (see our Editorial at https://dx.doi.org/10.1038/msb.2010.72), Molecular Systems Biology publishes online a Review Process File with each accepted manuscripts. This file will be published in conjunction with your paper and will include the anonymous referee reports, your point-by-point response and all pertinent correspondence relating to the manuscript. If you do NOT want this File to be published, please inform the editorial office at msb@embo.org within 14 days upon receipt of the present letter.

Reviewer #1:

The study described in the manuscript by Hassan and colleagues builds on their previously published observation that a bias related to mitochondrial complexes dominates gene essentiality profiles' similarities within cancer dependency map datasets generated via systematic pooled CRISPR-cas9 screens performed across hundreds of immortalised human cancer cell lines.

This bias masks subtle/weaker gene-pair similarities, thus hampering the power of such screens in revealing new networks of functionally related genes and cancer context-specific functional similarities. To tackle this issue, the authors propose to use unsupervised methods based on dimensionality reduction to normalise cancer dependency map datasets (across different public releases). They have tested and benchmarked different methods and developed their own technique (ONION normalisation) based on an ensemble model of different dimensionality reduction techniques whose contribution is tunable, showing that it outperforms state-of-the-art techniques.

The manuscript is well written, and the authors tackle an important, timely and relevant problem by providing a tool which might be useful to the community to derive functional genetics insights from large-scale CRISPR screens.

Whereas I do not question the goal, methodology and results presented in this manuscript, I have some major points that should be addressed/clarified before considering this manuscript further for publication in Molecular Systems Biology:

- I wonder what the scope of applicability of the ONION method is. Should the post-normalised data, in which the ribosomal protein bias and other potential biases have been removed, be used only to infer gene-gene interactions or functional modules or the authors advise using this method also to preprocess depmap data before conducting target/drug-discovery oriented analysis of CRISPR-Cas9 screen or other types of functional genetic studies? This should be clarified. In the second case, the authors should include additional analyses across their benchmarks to show that (at least) strictly context-specific dependencies (for example, statistically significant associations between mutations in known oncogenes and increased dependency on themselves or close interactors are preserved).

- The authors should also explore how their preprocessing methods impact tissue-specific similarities across cancer dependency profiles. For example, comparing the performances of a k-nearest classifier that tries to predict the tissue of origin of a cell line based on their most similar other cell lines (in terms for gene essentiality profiles) before/after applying the pre-processing methods.

- In addition, the authors do not investigate how/if/at-what-extent the heterogeneity of essentiality profiles of individual cell lines is impacted and, if so, in which sense.

- Finally, I'd be curious to see how cell-line-wise ROC/Precision-recall curves obtained benchmarking control always-essential/never-essential genes are impacted by the tested normalisation procedures.

- this study presents a degree of overlap/similarity with PMID: 35085500, which should be cited discussing differences/commonalities with the approaches presented in this study

Minor points:

- Page 5, line 112: "GI" should be explicitly defined at its first occurrence

- across figures, the presented curves are not, strictly speaking, Precision/Recall curves (as the x axis doesn't show recall levels but just True Positives). This should be amended/clarified

Reviewer #2:

"coessentiality" networks are functional interaction networks derived from genes with correlated knockout fitness profiles across a panel of CRISPR screens in diverse cell lines. These networks are analogous to functional interaction networks in yeast derived from correlated genetic interaction profiles across a panel of knockout strains. As with yeast, human correlation networks carry biases from the underlying data. Myers and colleagues have previously shown that the oxphos pathway, especially

Complex I and the mitochondrial ribosome, presents just such a bias, in no small part because their large complex sizes lead to a huge number of correlated gene pairs, which then dominate the calculated functional enrichment of the resulting correlation network. Here, Hassan et al explore three unsupervised dimensionality reduction methods for removing signals/bias from mitochondrial complexes and normalizing DepMap data. Authors propose an "onion" normalization technique to combine several normalized data layers into a single network. Benchmarking analyses shows that robust PCA combined with onion normalization outperforms existing methods for normalization.

There are several questions which should be addressed before publication:

- The authors used Depmap Ceres scores (20Q2 version) only. Is the Hassan approach robust to changes in the underlying data analysis? If the authors used Chronos (especially since Depmap only uses Chronos now) or other measures of knockout fitness defect instead, do the results change?
- The contribution diversity plots (the ones with red and blue contouring) need more explanation. As is, these plots are highly confusing, undermining the message that the authors intend to convey.
- From the contribution diversity plots, it seems that by removing the mitochondrial signals from the dataset, they are removing quite a lot of information. The authors must explain how much the underlying information is being removed by these approaches, and perhaps more saliently, must explain why the valid biological signal of the mitochondrial complexes needs to be removed from the data in the first place? In particular, please contrast with the approach of Gheorghe and Hart ,2022, who simply removed the offending annotations from the reference set used to calculate functional coherence of various network construction approaches.
- The authors compare only their onion-normalized networks (which include several normalized layers into one network) with Wainberg GLS (which is a single layer), but they do not compare their methods by themselves (without onion-ing) with Wainberg. What is the relative performance gain of the onion method over the individual component methods?

Minor points:

- In their Robust PCA methods, they vary the parameter λ. In the text and figures, that parameter is varied at values: 0.0049, 0.0056, 0.0063, 0.007, 0.0077, 0.0084, 0.0091. In their data repository Zenodo, is listed as: normalized_rpca_xx.tsv (*xx=0.7,0.8,0.9,1,1.1,1.2,1.3) ; which is confusing. All other parameters for their other methods match with the data files in Zenodo.
- Methods are explained well, and authors provide links to Github repositories for the Onion and Autoencoder normalization implementations.

Point-by-Point Response to Reviewer Comments

Reviewers' original comments are in blue.
Our responses are in black.
Text added to the main manuscript is in purple.

Reviewer #1:

The study described in the manuscript by Hassan and colleagues builds on their previously published observation that a bias related to mitochondrial complexes dominates gene essentiality profiles' similarities within cancer dependency map datasets generated via systematic pooled CRISPR-cas9 screens performed across hundreds of immortalised human cancer cell lines.

This bias masks subtle/weaker gene-pair similarities, thus hampering the power of such screens in revealing new networks of functionally related genes and cancer context-specific functional similarities. To tackle this issue, the authors propose to use unsupervised methods based on dimensionality reduction to normalise cancer dependency map datasets (across different public releases). They have tested and benchmarked different methods and developed their own technique (ONION normalisation) based on an ensemble model of different dimensionality reduction techniques whose contribution is tunable, showing that it outperforms state-of-the-art techniques.

The manuscript is well written, and the authors tackle an important, timely and relevant problem by providing a tool which might be useful to the community to derive functional genetics insights from large-scale CRISPR screens.

Whereas I do not question the goal, methodology and results presented in this manuscript, I have some major points that should be addressed/clarified before considering this manuscript further for publication in Molecular Systems Biology:

Major points:

1

I wonder what the scope of applicability of the ONION method is. Should the post-normalised data, in which the ribosomal protein bias and other potential biases have been removed, be used only to infer gene-gene interactions or functional modules or the authors advise using this method also to preprocess depmap data before conducting target/drug-discovery oriented analysis of CRISPR-Cas9 screen or other types of functional genetic studies? This should be clarified. In the second case, the authors should include additional analyses across their benchmarks to show that (at least) strictly context-specific dependencies (for example, statistically significant associations between

mutations in known oncogenes and increased dependency on themselves or close interactors are preserved).

We thank the reviewer for this helpful comment, and we agree that the scope of applications for our method wasn't clearly defined in our original manuscript. The normalization methods described in the paper (PCA, RPCA, AE, and Onion) were designed with the objective of enhancing functional information captured by gene-gene similarity networks derived from collections of CRISPR screen data, and the evaluations presented in the original manuscript were designed to evaluate performance on that task. Based on the reviewer's insightful comment #2, we also explored the extent to which cell line similarity networks could be improved with our methods, and indeed found evidence they could be (see more details in our response #2 below). We agree that exploring the impact of this normalization approach on other downstream applications is an interesting direction for future work, but we believe this is beyond the scope of the current manuscript. To make this clear, we've added sections to the introduction and to the discussion section that explicitly state that the scope of applications for our methods are improving similarity networks on either the gene or cell line dimension.

We add the following lines in the **main text Introduction section** (page 4, paragraph 2) to clarify the utility of our methods.

The goal of the proposed onion normalization methods is to enable the construction of improved gene-gene similarity networks from the DepMap dataset, which has been a major recent focus of analyses of these data (Boyle et al, 2018; Wainberg et al, 2021; Gheorghe & Hart, 2022) but we note is distinct from other important applications of the DepMap goals such as direct clustering of the cell lines/genes (Pan, et al., 2022), or more focused target/drug-discovery oriented analyses (Chiu et al, 2021; Ma et al, 2021; Shimada et al, 2021).

We add the following lines in the **main text Discussion section** (page 14, paragraph 2) to clarify the utility of our methods.

We emphasize that the main purpose of the proposed onion normalization methods is to enable the construction of improved gene-gene similarity networks from the DepMap dataset. Our analysis also suggests it can also be used for refining cell-line level similarity networks (e.g., for identification of cell lines that exhibit common dependencies). However, there are many other important applications of the DepMap data including direct clustering of the cell lines/genes, more focused target/drug-discovery oriented analyses, or analysis of individual genetic dependencies identified by the DepMap profiles. Onion normalization is not applicable to many of those other downstream applications.

2

The authors should also explore how their preprocessing methods impact tissue-specific similarities across cancer dependency profiles. For example, comparing the performances of a k-nearest classifier that tries to predict the tissue of origin of a cell line based on their most similar

other cell lines (in terms for gene essentiality profiles) before/after applying the pre-processing methods.

We thank the reviewer for this excellent suggestion. We performed the suggested analysis and indeed found that cell line similarity networks can also be improved using our normalization approach. Specifically, we applied our best-performing method, RPCO (RPCA followed by onion normalization), to the cell line dimension instead of the gene dimension of DepMap matrix. We then implemented a simple k-nearest neighbor classifier based on the resulting normalized similarity network and evaluated performance in predicting tissue of origin for each cell line. The results indicate a substantial increase in the median F1 score to tissue-of-origin prediction (from 0.2 to 0.4 for k=5) and for 24 out of 26 tissues, the normalization resulted in equal or better performance. This indicates that cell-line similarity networks also benefit from our proposed normalization approach. We added a section describing this additional line of analysis to the manuscript the results section and added two related figures (Figs. EV4 and EV5). We appreciate the reviewer suggesting this line of analysis.

We include the following sub-section in the manuscript **main text** (page 12, paragraph 2) to describe our findings in this context:

Onion-normalization improves prediction of cell lines' tissue-of-origin
Our previous analyses focused on refinement of gene similarity networks derived from the DepMap data. We reasoned that onion normalization may also improve detection of similarities between cell lines' dependency profiles. Previous work has explored the extent to which cell lines with similar mutations or similar tissues-of-origin exhibit common dependencies (e.g., Dharia *et al*, 2021). To test this, we implemented a simple K-nearest neighbor (kNN) classifier to predict tissue-of-origin from dependency profiles and optimized the choice of k (see Methods). The kNN classifier was provided either similarity based on the un-normalized dataset, or a similarity network derived from RPCO normalization applied to the cell line similarity matrix based on the DepMap 20Q2 dataset (Meyers *et al*, 2017; Dempster *et al*, 2019b) (Data ref: (Broad DepMap, 2020)). We evaluated precision-recall statistics for each possible tissue-of-origin, which reflects the ability of the kNN to correctly predict the corresponding tissue-of-origin based on each cell line's nearest neighbors. We found that the RPCO-normalized network supported a substantial increase in the median F1 score for tissue-of-origin prediction (from 0.2 to 0.4 for k=5) and for 24 out of 26 tissues, the normalization resulted in equal or better performance (Figure EV4, Figure EV5). This indicates that cell-line similarity networks also benefit from onion normalization.

We also include the following sub-section in the **Methods section** (page 19, paragraph 4) to clarify our analysis technique.

Analysis on cell-line similarity network
We applied RPCA (Candès *et al*, 2011) to DepMap 20Q2 (Data ref: (Broad DepMap, 2020)) cell-line profiles (Ceres scores across genes) and generated seven normalized layers (cell-lines x genes) by setting hyperparameter λ of the RPCA method to $f \div \sqrt{\max(r,c)}$, where r = 769, c = 18119, and f = 0.7,

0.8, 0.9, 1, 1.1, 1.2, 1.3. Seven cell-line similarity networks were created from the normalized data using Pearson Correlation Coefficient as a similarity metric and integrated using SNF (Wang *et al*, 2014a) ($\sigma = 0.5$, $k = 5$) to generate a RPCO-normalized network. For the tissue-lineage prediction task, a tissue label is assigned to a cell-line using k-nearest neighbor with majority voting. The highest similarity score neighbor label is assigned in case of a tie. The overall precision, recall and F1 scores are calculated using weighted mean of scores from individual classes. The baseline prediction scores are calculated by random classifier and taking the average of 100 iterations. The true tissue labels for cell-lines are derived from the sample_info.csv file provided with DepMap 20Q2.

To support our results, we include two expanded view **figures (Figure EV4 and Figure EV5)** illustrating the KNN classifier results from the analysis. Here are the figure legends (page 28, paragraph 6; page 29, paragraph 2) associated with the figures--

Figure EV4: K-nearest neighbor classifier tissue-lineage prediction results comparing un-normalized and RPCO-normalized DepMap 20Q2 (Data ref: (Broad DepMap, 2020)) cell-line similarity networks.
A, Overall F1, Precision, and Recall scores (weighted mean across classes) across different values of K (x-axis). Dashed line represents the scores from a baseline classifier.
B, Class-level (tissue-lineage) F1, Precision, and Recall scores for K=5 where X-axis depicts tissue lineage.

Figure EV5: Confusion matrix from K-nearest neighbor classifier (K=5) showing prediction results for each class (tissue-lineage) of cell-lines from un-normalized and RPCO-normalized DepMap 20Q2 (Data ref: (Broad DepMap, 2020)) cell-line similarity networks. X- and y-axes are class labels. The top row depicts the true number of cell-lines in each class, and the right-most column is the number of predicted cell-lines for each class. The diagonal represents true positives. Each row represents false positives, and each column represents false negatives sans the diagonal entry.

3

In addition, the authors do not investigate how/if/at-what-extent the heterogeneity of essentiality profiles of individual cell lines is impacted and, if so, in which sense.

We thank the reviewer for recommending this line of analysis. To explore this question, we calculated the Pearson correlation coefficient between all pairs of cell-line profiles based on the un-normalized data as well as seven cell-line level RPCA-normalized data (generated for a range of RPCA parameter 'lambda' values). Observing the distribution of the PCC scores, the average PCC of cell line-to-cell line similarity drops from ~0.9 for the un-normalized version to 0 in post-normalization (see Figure 1).

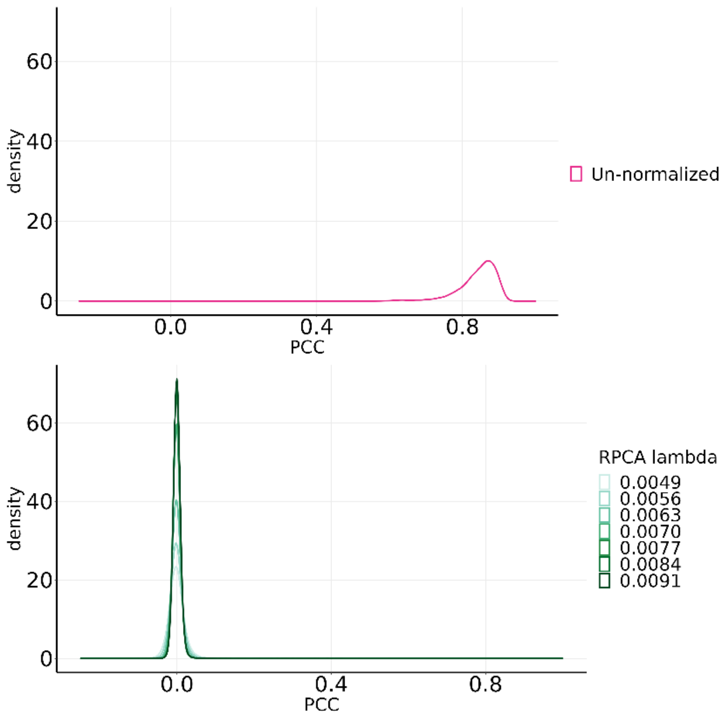

Figure 1. Distribution of Pearson Correlation Coefficients scores for pairs of cell lines from un-normalized and RPCA-normalized data.

This indicates that cell lines' essentiality profiles generally exhibit a relatively high agreement pre-normalization (unsurprisingly, as the signature of core essential genes is consistent across all cell lines), but once our normalization is applied, the essentiality profiles become much more heterogeneous. Our normalization is basically removing the low-dimensional core essential gene profile as well as other low-dimensional components (this is also supported by the results of our response #4 below). Based on our observations about tissue-of-origin prediction performance (see point #2 above), the similarity that is retained post-normalization tends to indicate more specific relationships between cell lines. This was a worthwhile analysis for our own understanding, but we don't feel it contributes to the narrative beyond the new results/figures added from our response #2, so we haven't added this figure to the manuscript.

4

Finally, I'd be curious to see how cell-line-wise ROC/Precision-recall curves obtained benchmarking control always-essential/never-essential genes are impacted by the tested normalisation procedures.

As the reviewer suggested, we benchmarked the normalized data for discrimination of always-essential/never-essential genes. Specifically, we applied RPCA-normalization to generate normalized profiles for each cell line but stopped before computing similarity between either genes

or cell lines. For each cell line's profile, we generated an AUROC score for both the pre- and post-normalization reflecting the classification of core-essential genes from gold standard non-essential genes retrieved from Hart et al. [1]. The average pre-normalization AUROC score across cell lines is 0.97 whereas the average post-normalization AUROC score decreased to 0.55 (see Figure 2). This indicates that RPCA-normalization is largely removing the core essential gene signature from each cell line's profile. From our evaluations of gene similarity networks or cell line similarity networks, this apparently improves the specificity and sensitivity of that similarity information.

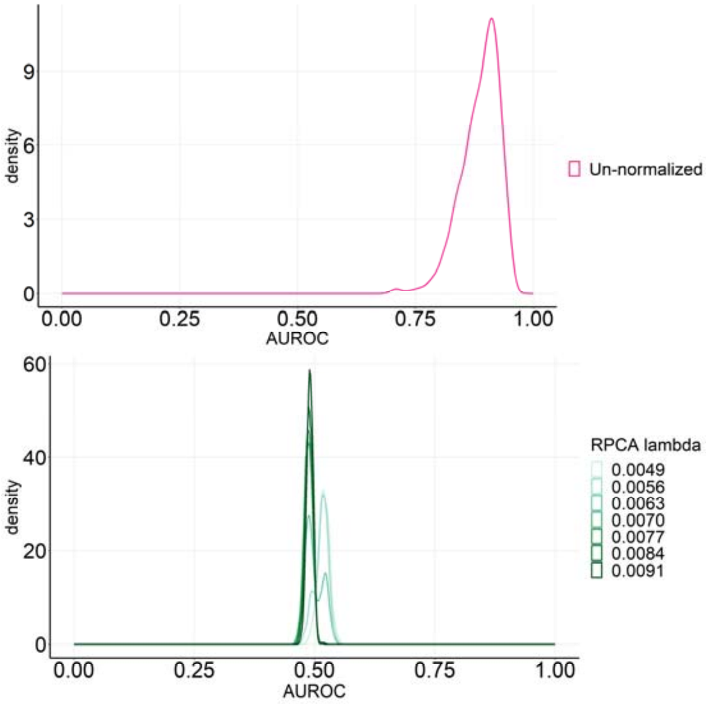

Figure 2: Distribution of AUROC scores across cell-line profiles from un-normalized and RPCA-normalized data

However, if one wants to actually distinguish true essential genes from non-essential genes in any given context, applying our normalization method to the data isn't appropriate. We thank the reviewer for suggesting this line of analysis as it helped to clarify the scope of our method, which is best fit for deriving similarity from these profiles. As described in our response to comment #1, we have added clarifications on the scope of the method to both the introduction and the discussion section of the manuscript.

[1] Hart, T., Brown, K. R., Sircoulomb, F., Rottapel, R., & Moffat, J. (2014). Measuring error rates in genomic perturbation screens: gold standards for human functional genomics. Molecular systems biology, 10(7), 733.

5

this study presents a degree of overlap/similarity with PMID: 35085500, which should be cited discussing differences/commonalities with the approaches presented in this study

We thank the reviewer for bringing the Pan et al. [1] paper to our attention. The method by Pan et al. [1] decomposes gene essentiality data across cell lines into two matrices - a low dimensional matrix depicting loss-of-function effect scores across cell lines and a sparse matrix of weighted mapping of genes to functions. The objective of this is to discover and cluster functional mappings, leveraging the low dimensional space. On the other hand, the goal of our method is to construct informative similarity networks, either on the gene dimension or the cell line dimension. Since they have quite different goals, a direct comparison of the two approaches isn't feasible. The methods we are comparing to in the manuscript are designed for exactly the same goal of measuring similarity amongst genes. We agree with the reviewer that Pan et al. should be cited and that we should conceptually distinguish our approach in our manuscript. We've added the section below to the introduction in our revised manuscript.

[1] Pan, Joshua, et al. "Sparse dictionary learning recovers pleiotropy from human cell fitness screens." Cell systems 13.4 (2022): 286-303.

We added the following lines in the **main text** Introduction section (page 4, paragraph 2) to contrast the mentioned method with ours.

The goal of the proposed onion normalization methods is to enable the construction of improved gene-gene similarity networks from the DepMap dataset, which has been a major recent focus of analyses of these data (Boyle et al, 2018; Wainberg et al, 2021; Gheorghe & Hart, 2022) but we note is distinct from other important applications of the DepMap goals such as direct clustering of the cell lines/genes (Pan, et al., 2022), or more focused target/drug-discovery oriented analyses (Chiu et al, 2021; Ma et al, 2021; Shimada et al, 2021).

Minor points:

1

Page 5, line 112: "GI" should be explicitly defined at its first occurrence

We thank the reviewer for this suggestion. We replaced GI with "genetic interaction" at that point (page 5, paragraph 1, line 118) of the manuscript.

2

across figures, the presented curves are not, strictly speaking, Precision/Recall curves (as the x axis doesn't show recall levels but just True Positives). This should be amended/clarified

Thanks for pointing this out. We agree we should clarify this to avoid confusion.

In the revised manuscript, we modified the legends of main **Figures 1C-D, 2A-B, 3B, 4A and Appendix Figures S9, S9, S11, S12, S13, S14, S15, S16** with the following text:

“The x-axis depicts the absolute number of true positives (TPs) recovered in log scale.”

We also added the following lines to the **Methods section (subsection: Functional evaluations)** (page 18, paragraph 2) to clarify the PR curve’s x-axis -
For all PR analysis plots, we plot precision on the y-axis and the absolute number of true positives (TPs) on the x-axis. We note that this deviates from the strict definition of recall, which is a fractional value (number of TPs divided by the total number of positive examples). In this context, we prefer to plot the absolute number of TPs to provide more information about the total number of gene pairs captured at each threshold. The x-axis would simply be scaled by a single constant if recall were plotted instead.

Reviewer #2:

"coessentiality" networks are functional interaction networks derived from genes with correlated knockout fitness profiles across a panel of CRISPR screens in diverse cell lines. These networks are analogous to functional interaction networks in yeast derived from correlated genetic interaction profiles across a panel of knockout strains. As with yeast, human correlation networks carry biases from the underlying data. Myers and colleagues have previously shown that the oxphos pathway, especially Complex I and the mitochondrial ribosome, presents just such a bias, in no small part because their large complex sizes lead to a huge number of correlated gene pairs, which then dominate the calculated functional enrichment of the resulting correlation network. Here, Hassan et al explore three unsupervised dimensionality reduction methods for removing signals/bias from mitochondrial complexes and normalizing DepMap data. Authors propose an "onion" normalization technique to combine several normalized data layers into a single network. Benchmarking analyses shows that robust PCA combined with onion normalization outperforms existing methods for normalization.

There are several questions which should be addressed before publication:

Major points:

1

The authors used Depmap Ceres scores (20Q2 version) only. Is the Hassan approach robust to changes in the underlying data analysis? If the authors used Chronos (especially since Depmap only uses Chronos now) or other measures of knockout fitness defect instead, do the results change?

We thank the reviewer for this suggestion and agree that it is important to assess the performance of our approach on the newest versions of DepMap data that use the Chronos score. To address this comment, we applied RPCA and RPCO normalization methods as well as the strongest competing method (GLS) on DepMap 2022 q4 Chronos single knockout fitness scores and evaluated the output

networks using FLEX. The results are qualitatively similar to our findings based on the DepMap 2020 q2 Ceres scores.

We added two **Appendix supplementary figures (Appendix Figure S11 and Appendix Figure S12)** and the following figure legends (see Appendix.pdf page 22, paragraph 1; page 24, paragraph 1) to illustrate the findings -

Appendix Figure S11: Exploration of mitochondrial bias within the DepMap 22Q4 Chronos (Data ref: Broad DepMap, 2022) and RPCA normalization across hyperparameters.
A, (Left) Precision- recall (PR) performance analysis of un-normalized DepMap 22Q4 Chronos gene similarity network evaluated against CORUM protein complex standard. The x-axis depicts the number of true positives (TPs) in log-scale. (Right) Contribution diversity plot of CORUM complexes in un-normalized DepMap data. This plot is constructed by sliding a precision cutoff from high to low (indicated by the y-axis), and at each point, plotting a stacked bar plot across the x-axis at that point reflecting the breakdown of complex membership of the TP pairs identified at that threshold. The top ten contributing complexes are listed in the legend, with the light gray category representing all complexes represented at lower frequency.
B, (Left) PR performance analysis of RPCA-normalized data generated with hyperparameter λ set to 0.0049, 0.007 and 0.0091 evaluated against CORUM protein complex as standard. (Right) Corresponding contribution diversity plots illustrating complex contributions in RPCA-reconstructed and RPCA-normalized data. The x-axis depicts the absolute number of true positives (TPs) recovered in log scale.

Appendix Figure S12: Onion-normalization applied to DepMap 22Q4 Chronos data (Data ref: Broad DepMap, 2022) with RPCA as the normalization technique.
A, FLEX precision-recall (PR) performance analysis of original DepMap 22Q4 Chronos data, onion normalization with robust PCA (RPCO), and generalized least squares (GLS) normalization (Wainberg *et al*, 2021) evaluated against CORUM protein complex standard. The x-axis depicts the absolute number of true positives (TPs) recovered in log scale.
B, Contribution diversity of CORUM complexes for the original DepMap, RPCO, and GLS. Fractions of predicted true positives (TP) from different complexes are plotted at various precision levels on the y-axis.
C, Number of complexes for which area under the PR curve (AUPRC) values increase and decrease with respect to chosen AUPRC thresholds due to normalization as compared to un-normalized data. The bars on the left side of the dotted line correspond to RPCA-normalized layers ($\lambda \tilde{=} 0.0056$, 0.0064, 0.0072, 0.0080, 0.0088, 0.0096, 0.0104). The bars on the right side of the dotted line correspond to the RPCO-normalized data from the layers as well as GLS data. The color gradient for each method represents four bins with complexes containing 2 to 3 genes, 4 to 5 genes, 6 to 9 genes, and 10 or more genes. (Left) AUPRC threshold, $t = 0.1$. (Right) $t = 0.5$.

We added the following lines in the **main text (Section: Results; Subsection: Onion normalization integrates normalized data across hyperparameter values)** (page 11, paragraph 1):

Furthermore, we found similar performance from RPCA- and RPCO-normalization techniques when applied to a more recent version of the DepMap (DepMap 2022 Q4 Chronos scores) (Meyers et al, 2017; Dempster et al, 2019b, 2021; Pacini et al, 2021) (Data ref: (Broad DepMap, 2022)) and benchmarked against GLS, confirming that the RPCO-normalization is robust across DepMap scoring pipelines (Appendix Figure S11, Appendix Figure S12).

We also updated the **Methods section** (page 17, paragraph 3) to add the following subsection:

Normalization of DepMap 2022 Q4 Chronos scores
We investigated the effect of RPCA-normalization, RPCO-normalization as well as the GLS method (Wainberg et al, 2021) on the DepMap 2022 Q4 Chronos single KO effect scores (CRISPRGeneEffect.csv) (Data ref: (Broad DepMap, 2022)). Applying RPCA-normalization, we generated seven normalized layers (gene x cell-lines) by setting hyperparameter λ of the RPCA method to $f \div \sqrt{\max(r,c)}$, where r =17453, c=1078, and f = 0.7, 0.8, 0.9, 1, 1.1, 1.2, 1.3. These normalized data were then converted into seven gene-gene profile similarity networks using Pearson Correlation Coefficients as the similarity metric. These networks were integrated using the SNF method ($\sigma = 0.5$, $k = 5$) to create the RPCO-normalized network. The GLS pipeline was applied to the same 2022 q4 Chronos DepMap dataset using the standard settings.

2

The contribution diversity plots (the ones with red and blue contouring) need more explanation. As is, these plots are highly confusing, undermining the message that the authors intend to convey.

We thank the reviewer for this suggestion. We agree that these figures convey crucial information and that our descriptions of them could be improved to clarify their interpretations.

Already in **Main Figure** 1 legend --
'Contribution diversity plot of CORUM complexes in un-normalized DepMap data. This plot is constructed by sliding a precision cutoff from high to low (indicated by the y-axis), and at each point, plotting a stacked bar plot across the x-axis at that point reflecting the breakdown of complex membership of the TP pairs identified at that threshold. The top ten contributing complexes are listed in the legend, with the light gray category representing all complexes represented at lower frequency.'

We added the following lines to the manuscript **main text (section: Results; subsection: Removing low-dimensional signal from the DepMap boosts the performance of non-mitochondrial complexes)** (page 6, paragraph 1):

For example, the diversity plot shows that about 80% of the true-positive gene pairs at precision point 0.8 are from gene pairs belonging to mitochondrial complexes. Specifically, the majority of true positive gene pairs at various precision cut-offs are annotated to be in 55S ribosome and respiratory chain mitochondrial complexes represented by the large red area across the plot. These

two complexes are the highest contributing complexes in terms of true positive pairs and contribute a disproportionate amount to the strong PR curve performance.

We also updated the **Methods section (subsection: Functional evaluations)** (page 18, paragraph 2) to add the following text:

This visualization is augmented by contribution diversity plots, which illustrate specific complexes that contribute to true positive (TP) gene pairs at various precision points on the y-axis. These plots are constructed by sliding a precision cutoff from high to low (indicated by the y-axis), and at each point, plotting a stacked bar plot across the x-axis at that point reflecting the breakdown of complex membership of the TP pairs identified at that threshold. For example, if there are X total TP gene-pairs at a cutoff that results in a precision of 0.8, the diversity plot will contain a stacked bar plot centered at y=0.80 stretching across the x-axis where each section of the bar plot represents the fraction of pairs contributed by a specific protein complex amongst those X TP pairs. This stacked bar plot is recomputed at each precision point to reflect the set of TP pairs satisfying the corresponding cutoff. As a result, a visually larger area from a complex denotes more TP contribution from that complex across the y-axis. In all diversity plots across the manuscript, the top ten contributing complexes are shown in red or blue shades and all other complexes contributing at a lower frequency are represented in gray.

3

From the contribution diversity plots, it seems that by removing the mitochondrial signals from the dataset, they are removing quite a lot of information. The authors must explain how much the underlying information is being removed by these approaches, and perhaps more saliently, must explain why the valid biological signal of the mitochondrial complexes needs to be removed from the data in the first place? In particular, please contrast with the approach of Gheorghe and Hart ,2022, who simply removed the offending annotations from the reference set used to calculate functional coherence of various network construction approaches.

We thank the reviewer for these interesting comments and suggestions.

How much the underlying information is being removed by these approaches?

There are a variety of ways to quantify how much information we're gaining/losing by applying our normalization approach. We present multiple complementary metrics for this, each of which is summarized below:

(1) Global PR curve, including mitochondrial pairs (equivalent of a micro-average PR measure): This measure suggests we are losing some information by applying RPCO normalization, depending on the level of precision desired (see **main Fig. 3B, left side** (standard: CORUM co-complex pairs)). For example, at 0.5 precision, the un-normalized data is able to recover ~4000 TP pairs while the onion-normalized network is able to recover ~2000 TP pairs. However, at 0.2 precision (~34-fold over random), the normalized network recovers ~9k TPs while the un-normalized network

recovers ~8.5k TPs. Thus, whether we're gaining or losing information here depends on the precision cutoff we set. Where the un-normalized network has more information (in the >0.5 precision range), those pairs are a large majority (~80%) composed of mitochondria-related genes (see **main Fig. 3C, Un-normalized** (standard: CORUM co-complex pairs)).

(2) Global PR curve, excluding mitochondrial pairs:
This measure suggests we gain information by applying onion normalization across a wide range of precision cutoffs (see **main Fig. 3B, right side** (standard: CORUM co-complex pairs; TPs that involve two mitochondria-related genes are not counted as true or false positives)). For example, at 0.5 precision, the onion-normalized network is able to recover ~1500 TP pairs while the un-normalized network is able to recover ~70 TP pairs. At 0.2 precision, the onion-normalized network is able to recover ~9000 TP pairs while the un-normalized network is able to recover ~250 TP pairs. What this means is if we set aside mitochondria-related gene pairs (i.e., we don't count TPs that connect pairs of mitochondria-related genes), we are gaining 20-30-fold in terms of true positives at a range of precision thresholds by applying onion normalization.

(3) Individual category PR curve (AUPRC) (similar to a macro-average measure):
For this evaluation, we measure precision-recall on each complex within the CORUM complex standard (considering only pairs that involve at least one gene in that complex) and then compute a summary AUPRC measure per complex. In **main Fig. 3D** we plot the number of complexes for which AUPRC_normalized > 0.1 and AUPRC_un-normalized <= 0.1 (left side t=0.1) as well as AUPRC_normalized > 0.5 and AUPRC_un-normalized <= 0.5 (right side t=0.5). The positive bar indicates the number of complexes that improved and crossed that threshold after normalization. The negative bar indicates the number of complexes that decrease in per-complex AUPRC performance after normalization relative to un-normalized. This evaluation strongly suggests we are gaining information by applying onion normalization. For example, for the t=0.1 threshold, RPCO results in improvements for ~150 complexes while it results in decreases for ~30 complexes relative to the un-normalized networks. At the 0.5 threshold, RPCO results in improvements for ~30 complexes while it results in decreases for ~5 complexes. This per-complex analysis actually indicates stronger performance for the RPCO-normalized network for several mitochondria-related complexes. For example, the normalized network achieves a 0.56 for the Respiratory chain complex I as compared to a 0.47 for the un-normalized network, while RPCO achieves 0.71 for the 55S mitochondrial ribosome as compared to 0.58 for the un-normalized network. Thus, by this measure, onion normalization actually improves the precision/recall in identifying even mitochondrial gene relationships.

To summarize these findings, we conclude that by most measures, onion normalization is substantially improving the amount of information we can capture with similarity networks.

Why does valid biological signal of the mitochondrial complexes need to be removed in the first place?

This is an interesting question. Our evaluations clearly tell us that when we remove these low-dimensional patterns from the data, it boosts our ability to capture a diverse set of other functional

relationships. This suggests that in addition to causing similarity amongst mitochondria-related genes, this low-dimensional signal is obscuring our detection of relationships amongst other genes. One potential explanation is that unrelated, non-mitochondrial genes may also contain portions of the same low-dimensional signal, which causes unspecific correlations between them and a large set of mitochondrial genes.

To explore this hypothesis, we performed a simple analysis of false positives for the un-normalized PCC network and the RPCO-normalized network. Specifically, we thresholded the similarity values at several different cutoffs to create similarity networks of varying densities (from 99.5 percentile up to 99.995 percentile - from ~1 million total edges down to ~1000 total edges). We evaluated the resulting networks against a CORUM complex standard to identify sets of (true positives-TP, false positives-FP, and unlabeled) pairs amongst the similarity network edges. Then, for each network, we measured the fraction of the edges called FP (i.e., they connected two genes with no evidence of a functional relationship in our complex standard) for which only one of those genes was mitochondrial. We found that in the un-normalized networks, a large fraction of the false positives called involved a mitochondrial gene, and this fraction increased substantially for the strongest edges in the network (e.g., >40% of the FP edges for a ~20k edge network involve mitochondria-related genes) (see Figure 3 below). This supports the idea that the same signal that correlates truly related mitochondrial gene pairs is also causing correlation between mitochondria genes and otherwise unrelated other genes (at least according to our current functional standards). When we normalize out those non-specific patterns, we're left with more subtle, specific signals that correlate genes with known shared functions, which improves our PR performance for many non-mitochondrial complexes.

This raises the related question of the source of this signal. In our earlier paper (Rahman et al. 2021 [2]), we hypothesized that one potential source of the strong mitochondria gene signal is that electron transport chain complex proteins are amongst the most stable protein complexes in the proteome in terms of half-life. We reasoned that this could result in delayed phenotypes and variable penetrance in CRISPR screens depending on technical factors (e.g., exact screen length, doubling time of the cell line). If this is true, one might expect other highly stable proteins with unrelated functions to exhibit similarly variable phenotypes, which would introduce covariation across the DepMap. Definitively identifying the source of these patterns will require further experiments and is beyond the scope of this paper. The important contributions of our paper are to demonstrate the utility in measuring similarity at multiple different "layers" in CRISPR screen profiles, that integrating information across these later can improve precision/recall in detecting known functional relationships across a variety of biological processes, and finally, that this layering can be done in an unsupervised manner (we used no information about mitochondrial genes to normalize this signal).

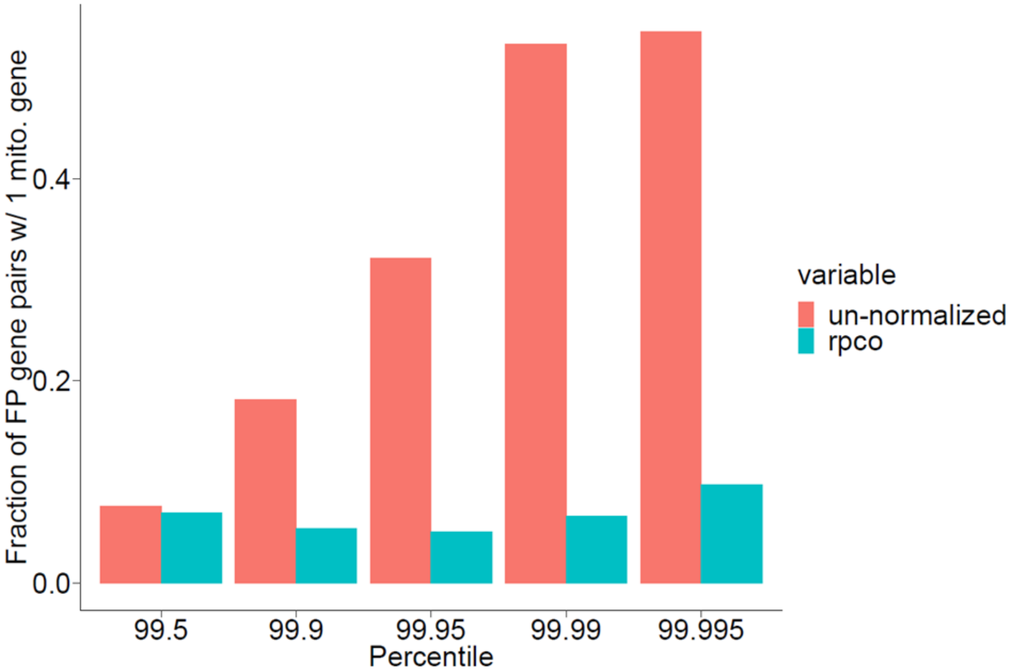

Figure 3: Fraction of false positive gene pairs with only one mitochondrial gene at different percentile cut-offs in un-normalized and RPCO-normalized networks

Please contrast with the approach of Gheorghe and Hart ,2022, who simply removed the offending annotations from the reference set used to calculate functional coherence of various network construction approaches.

The evaluation approach we use for several of our analyses (excluding mitochondrial pairs from the evaluation, e.g., in **main Fig. 3B, right side**) is very similar to the approach of Gheorghe and Hart 2022 [1] and is the same as what we proposed in the original FLEX manuscript (Rahman et al. 2021 [2]). Masking the mitochondrial genes from evaluation to measure signal for other processes is important. However, our onion normalization provides an important boost in signal regardless of how the evaluation is conducted. For example, consider the difference between the "un-normalized" and "RPCO" curves in **main Fig. 3B, right**, which implements the mitochondrial-masking approach: here we gain 20-30-fold in recall across a broad range of precision thresholds by performing our normalization. Thus, only modifying how you evaluate networks misses out on substantial additional functional signal that can be extracted from these data if normalized well.

[1] Gheorghe, Veronica, and Traver Hart. "Optimal construction of a functional interaction network from pooled library CRISPR fitness screens." BMC bioinformatics 23.1 (2022): 1-15.
[2] Rahman, Mahfuzur, et al. "A method for benchmarking genetic screens reveals a predominant mitochondrial bias." Molecular Systems Biology 17.5 (2021): e10013.

4

The authors compare only their onion-normalized networks (which include several normalized layers into one network) with Wainberg GLS (which is a single layer), but they do not compare their

methods by themselves (without onion-ing) with Wainberg. What is the relative performance gain of the onion method over the individual component methods?

We thank the reviewer for this comment. We added an additional supplemental figure (Appendix Figure S9) that directly plots the performance of individual layers relative to the performance of the complete RPCO, GLS, and OLF approaches. These figures support the following conclusions: (1) OLF is generally outperformed in all evaluations by both individual RPCA layers as well as the combined RPCO results; (2) GLS outperforms some of the individual RPCA layers (e.g., λ = 0.0049); (3) the combined RPCO results (after onion-ing of the layers) outperforms all individual RPCA layers and GLS.

We include **Appendix figure S9** and add the following figure legend (see Appendix.pdf page 18, paragraph 1)-

Appendix Figure S9: The performance comparison of individual normalized layer networks with the networks from Onion-normalization and other methods applied to DepMap 20Q2 (Data ref: Broad DepMap, 2020).
A, FLEX precision-recall (PR) performance analysis of networks from original DepMap, robust PCA (RPCA) normalization with hyperparameter λ set to 0.0049, 0.007 and 0.0091, onion normalization with robust PCA (RPCO), generalized least squares (GLS) normalization (Wainberg *et al*, 2021), and olfactory receptor (OLF) normalization (Boyle *et al*, 2018) with CORUM protein complexes as the standard. (Left) All CORUM complex gene pairs as true-positive. (Right) Mitochondrial gene pairs are removed from the evaluation. The x-axis of both plots depicts the absolute number of true positives (TPs) recovered in log scale.
B, Number of complexes for which area under the PR curve (AUPRC) values increase and decrease with respect to chosen AUPRC thresholds due to normalization as compared to un-normalized data. The bars on the left side of the dotted line correspond to AE-normalized layers (latent space size = 1, 2, 3, 4, 5, 10), PCA-normalized layers (first 1, 3, 5, 7, 9, 11, 13, 15, 17, 19 principal components removed) and RPCA layers ($\tilde{\lambda}$= 0.0049, 0.0056, 0.0063, 0.007, 0.0077, 0.0084, 0.0091). The bars on the right side of the dotted line correspond to Onion-normalized data from the PCA, PRCA, and AE layers as well as GLS and OLF. The color gradient for each method represents four bins with complexes containing 2 to 3 genes, 4 to 5 genes, 6 to 9 genes, and 10 or more genes. (Top) AUPRC threshold, t = 0.1. (Bottom) t = 0.5.

We also added the following line to the manuscript **main text (Section: Results; Subsection: Onion normalization integrates normalized data across hyperparameter values)** (page 10, paragraph 3) and cite our supplementary figure:

Although several individual normalized layers from RPCA, PCA, and AE perform comparable to GLS, the combination of all layers (RPCO) results in the strongest performance and outperforms GLS (Appendix Figure S9, Appendix Figure S10).

Minor points:

1

In their Robust PCA methods, they vary the parameter λ. In the text and figures, that parameter is varied at values: 0.0049, 0.0056, 0.0063, 0.007, 0.0077, 0.0084, 0.0091. In their data repository Zenodo, is listed as: normalized_rpca_xx.tsv (*xx=0.7,0.8,0.9,1,1.1,1.2,1.3) ; which is confusing. All other parameters for their other methods match with the data files in Zenodo.

We thank the reviewer for catching this mismatch in file names. To remedy that, we added a note in our Zenodo repository that maps the file names with the parameters. The parameter λ is calculated using the formula $\lambda = f \div \sqrt{\max(r,c)}$, where r = # of rows, c=# of columns, and f = 0.7, 0.8, 0.9, 1, 1.1, 1.2, 1.3. Although we report the actual λ parameter values in the manuscript, our filenames reflect the f.

2

Methods are explained well, and authors provide links to Github repositories for the Onion and Autoencoder normalization implementations.

We thank the reviewer for taking the time to review our data and code repositories.

8th Aug 2023

Manuscript Number: MSB-2023-11657R
Title: Dimensionality reduction methods for extracting functional networks from large-scale CRISPR screens

Dear Chad,

Thank you for sending us your revised manuscript. We have now heard back from the two reviewers who were asked to evaluate your revised study. As you will see below, they are satisfied with the performed revisions and support publication. I am therefore glad to inform you that we can soon accept your manuscript for publication, pending some minor editorial issues listed below.

- Our data editors have noticed some unclear or missing information in the figure legends, please see the attached .doc file. We have also made some minor formatting edits. Please make all requested text changes using the attached file and *keeping the "track changes" mode* so that we can easily access the edits made.

- Our data integrity analyst noted a few instances of figure panel reuse i.e. in Figures 1C and 3C, Figures 1C and 4B, Figures 3C and 4B, Appendix Figures S11A and S12B. We would ask you to indicate the data/panel reuse in the respective figure legends for transparency.

- Please note that for the article type "Method" it is mandatory to use 'Structured Methods', our new Materials and Methods format, which is mandatory for Methods and Articles with a strong methodological focus. According to this format, the Material and Methods section should include a Reagents and Tools Table (listing key reagents, experimental models, software and relevant equipment and including their sources and relevant identifiers) followed by a Methods and Protocols section in which we encourage the authors to describe their methods using a step-by-step protocol format with bullet points, to facilitate the adoption of the methodologies across labs. More information on how to adhere to this format as well as downloadable templates (.doc or .xls) for the Reagents and Tools Table can be found in our author guidelines: . An example of a Method paper with Structured Methods can be found here: .

Please resubmit your revised manuscript online **within one month** and ideally as soon as possible. If we do not receive the revised manuscript within this time period, the file might be closed and any subsequent resubmission would be treated as a new manuscript. Please use the Manuscript Number (above) in all correspondence.

Click on the link below to submit your revised paper.

https://msb.msubmit.net/cgi-bin/main.plex

Thank you for submitting this paper to Molecular Systems Biology.

Kind regards,

Maria

Maria Polychronidou, PhD
Senior Editor
Molecular Systems Biology

If you do choose to resubmit, please click on the link below to submit the revision online before 7th Sep 2023.

https://msb.msubmit.net/cgi-bin/main.plex

IMPORTANT:
Please note that corresponding authors are required to supply an ORCID ID for their name upon submission of a revised manuscript (EMBO Press signed a joint statement to encourage ORCID adoption).
(https://www.embopress.org/page/journal/17444292/authorguide#editorialprocess)
Currently, our records indicate that the ORCID for your account is 0000-0002-1026-5972.

Please click the link below to modify this ORCID:
Link Not Available

The system will prompt you to fill in your funding and payment information. This will allow Wiley to send you a quote for the article processing charge (APC) in case of acceptance. This quote takes into account any reduction or fee waivers that you may be eligible for. Authors do not need to pay any fees before their manuscript is accepted and transferred to the publisher.

As a matter of course, please make sure that you have correctly followed the instructions for authors as given on the submission website.

*** PLEASE NOTE *** As part of the EMBO Press transparent editorial process initiative (see our Editorial at https://dx.doi.org/10.1038/msb.2010.72 , Molecular Systems Biology will publish online a Review Process File to accompany accepted manuscripts. When preparing your letter of response, please be aware that in the event of acceptance, your cover letter/point-by-point document will be included as part of this File, which will be available to the scientific community. More information about this initiative is available in our Instructions to Authors. If you have any questions about this initiative, please contact the editorial office (msb@embo.org).

Reviewer #1:

The authors have satisfactorily addressed the points I raised in the previous round of review by incorporating the requested clarifications and providing a more comprehensive explanation of the scope and aim of their methodology. Furthermore, they have integrated new analyses to more effectively substantiate their claims and have embraced the suggested changes.
I do believe that, as a result, the manuscript is ready to be published and it will make a great contribution to the field.

Reviewer #2:

We thank the authors for the comprehensive response to all reviewer queries, concerns, and conceptual confusions. This revision goes above and beyond. All concerns are addressed, and we find this version highly suitable for publication.

All editorial and formatting issues were resolved by the authors.

5th Sep 2023

RE: MSB-2023-11657RR, Dimensionality reduction methods for extracting functional networks from large-scale CRISPR screens

Dear Chad,

Thank you again for sending us your revised manuscript. We are now satisfied with the modifications made and I am pleased to inform you that your paper has been accepted for publication.

*** PLEASE NOTE *** As part of the EMBO Publications transparent editorial process initiative (see our Editorial at https://dx.doi.org/10.103/msb.2010.72), Molecular Systems Biology publishes online a Review Process File with each accepted manuscripts. This file will be published in conjunction with your paper and will include the anonymous referee reports, your point- by-point response and all pertinent correspondence relating to the manuscript. If you do NOT want this File to be published, please inform the editorial office at msb@embo.org within 14 days upon receipt of the present letter.

LICENSE AND PAYMENT:
All articles published in Molecular Systems Biology are fully open access: immediately and freely available to read, download and share.

Molecular Systems Biology charges an article processing charge (APC) to cover the publication costs. You, as the corresponding author for this manuscript, should have already received a quote with the article processing fee separately. Please let us know in case this quote has not been received.

Once your article is at Wiley for editorial production, you will receive an email from Wiley's Author Services system, which will ask you to log in and will present you with the publication license form for completion. Within the same system the publication fee can be paid by credit card, an invoice or pro forma can be requested.

Payment of the publication charge and the signed Open Access Agreement form must be received before the article can be published online.

Upon acceptance it is mandatory for you to return the completed payment form. Failure to send back the form may result in a delay in the publication of your paper.

Molecular Systems Biology articles are published under the Creative Commons licence CC BY, which facilitates the sharing of scientific information by reducing legal barriers, while mandating attribution of the source in accordance to standard scholarly practice.

Proofs will be forwarded to you within the next 2-3 weeks.

Thank you very much for submitting your work to Molecular Systems Biology.

Kind regards,

Maria

Maria Polychronidou, PhD
Senior Editor
Molecular Systems Biology

EMBO Press Author Checklist

Corresponding Author Name: Chad L. Myers	
Journal Submitted to: Molecular Systems Biology	
Manuscript Number: MSB-2023-11657	

USEFUL LINKS FOR COMPLETING THIS FORM
The EMBO Journal - Author Guidelines
EMBO Reports - Author Guidelines
Molecular Systems Biology - Author Guidelines
EMBO Molecular Medicine - Author Guidelines

Reporting Checklist for Life Science Articles (updated January
This checklist is adapted from Materials Design Analysis Reporting (MDAR) Checklist for Authors. MDAR establishes a minimum set of requirements in transparent reporting in the life sciences (see Statement of Task: 10.31222/osf.io/9sm4x). Please follow the journal's guidelines in preparing your
Please note that a copy of this checklist will be published alongside your article.

Abridged guidelines for figures
1. Data
The data shown in figures should satisfy the following conditions:
- the data were obtained and processed according to the field's best practice and are presented to reflect the results of the experiments in an accurate and unbiased manner.
- ideally, figure panels should include only measurements that are directly comparable to each other and obtained with the same assay.
- plots include clearly labeled error bars for independent experiments and sample sizes. Unless justified, error bars should not be shown for technical
- if n<5, the individual data points from each experiment should be plotted. Any statistical test employed should be justified.
- Source Data should be included to report the data underlying figures according to the guidelines set out in the authorship guidelines on Data

2. Captions
Each figure caption should contain the following information, for each panel where they are relevant:
- a specification of the experimental system investigated (eg cell line, species name).
- the assay(s) and method(s) used to carry out the reported observations and measurements.
- an explicit mention of the biological and chemical entity(ies) that are being measured.
- an explicit mention of the biological and chemical entity(ies) that are altered/varied/perturbed in a controlled manner.
- the exact sample size (n) for each experimental group/condition, given as a number, not a range;
- a description of the sample collection allowing the reader to understand whether the samples represent technical or biological replicates (including how many animals, litters, cultures, etc.).
- a statement of how many times the experiment shown was independently replicated in the laboratory.
- definitions of statistical methods and measures:
  - common tests, such as t-test (please specify whether paired vs. unpaired), simple χ2 tests, Wilcoxon and Mann-Whitney tests, can be unambiguously identified by name only, but more complex techniques should be described in the methods section;
  - are tests one-sided or two-sided?
  - are there adjustments for multiple comparisons?
  - exact statistical test results, e.g., P values = x but not P values < x;
  - definition of 'center values' as median or average;
  - definition of error bars as s.d. or s.e.m.

Please complete ALL of the questions below.
Select "Not Applicable" only when the requested information is not relevant for your study.

Materials

Newly Created Materials	Information included in the manuscript?	In which section is the information available? (Reagents and Tools Table, Materials and Methods, Figures, Data Availability Section)
New materials and reagents need to be available; do any restrictions apply?	Not Applicable	NA

Antibodies	Information included in the manuscript?	In which section is the information available? (Reagents and Tools Table, Materials and Methods, Figures, Data Availability Section)
For **antibodies** provide the following information:<br>- Commercial antibodies: RRID (if possible) or supplier name, catalogue number and or/clone number<br>- Non-commercial: RRID or citation	Not Applicable	NA

DNA and RNA sequences	Information included in the manuscript?	In which section is the information available? (Reagents and Tools Table, Materials and Methods, Figures, Data Availability Section)
**Short novel DNA or RNA including primers, probes**: provide the sequences.	Not Applicable	NA

Cell materials	Information included in the manuscript?	In which section is the information available? (Reagents and Tools Table, Materials and Methods, Figures, Data Availability Section)
**Cell lines:** Provide species information, strain. Provide accession number in repository **OR** supplier name, catalog number, clone number, and/**OR** RRID.	Not Applicable	NA
**Primary cultures:** Provide species, strain, sex of origin, genetic modification status.	Not Applicable	NA
Report if the cell lines were recently **authenticated** (e.g., by STR profiling) and tested for mycoplasma contamination.	Not Applicable	NA

Experimental animals	Information included in the manuscript?	In which section is the information available? (Reagents and Tools Table, Materials and Methods, Figures, Data Availability Section)
**Laboratory animals or Model organisms:** Provide species, strain, sex, age, genetic modification status. Provide accession number in repository **OR** supplier name, catalog number, clone number, **OR** RRID.	Not Applicable	NA
**Animal observed in or captured from the field:** Provide species, sex, and age where possible.	Not Applicable	NA
Please detail **housing and husbandry conditions**.	Not Applicable	NA

Plants and microbes	Information included in the manuscript?	In which section is the information available? (Reagents and Tools Table, Materials and Methods, Figures, Data Availability Section)
**Plants:** provide species and strain, ecotype and cultivar where relevant, unique accession number if available, and source (including location for collected wild specimens).	Not Applicable	NA
**Microbes:** provide species and strain, unique accession number if available, and source.	Not Applicable	NA

Human research participants	Information included in the manuscript?	In which section is the information available? (Reagents and Tools Table, Materials and Methods, Figures, Data Availability Section)
If collected and within the bounds of privacy constraints report on age, sex and gender or ethnicity for all study participants.	Not Applicable	NA

Core facilities	Information included in the manuscript?	In which section is the information available? (Reagents and Tools Table, Materials and Methods, Figures, Data Availability Section)
If your work benefited from core facilities, was their service mentioned in the acknowledgments section?	Not Applicable	NA

Design

Study protocol	Information included in the manuscript?	In which section is the information available? (Reagents and Tools Table, Materials and Methods, Figures, Data Availability Section)
If study protocol has been **pre-registered, provide DOI in the manuscript**. For clinical trials, provide the trial registration number **OR** cite DOI.	Not Applicable	NA
Report the **clinical trial registration number** (at ClinicalTrials.gov or equivalent), where applicable.	Not Applicable	NA

Laboratory protocol	Information included in the manuscript?	In which section is the information available? (Reagents and Tools Table, Materials and Methods, Figures, Data Availability Section)
Provide DOI OR other citation details if **external detailed step-by-step protocols** are available.	Not Applicable	NA

Experimental study design and statistics	Information included in the manuscript?	In which section is the information available? (Reagents and Tools Table, Materials and Methods, Figures, Data Availability Section)
Include a statement about **sample size** estimate even if no statistical methods were used.	Not Applicable	NA
Were any steps taken to minimize the effects of subjective bias when allocating animals/samples to treatment (e.g. **randomization procedure**)? If yes, have they been described?	Not Applicable	NA
Include a statement about **blinding** even if no blinding was done.	Not Applicable	NA
Describe **inclusion/exclusion criteria** if samples or animals were excluded from the analysis. Were the criteria pre-established?<br><br>If sample or data points were omitted from analysis, report if this was due to attrition or intentional exclusion and provide justification.	Not Applicable	NA
For every figure, are **statistical tests** justified as appropriate? Do the data meet the assumptions of the tests (e.g., normal distribution)? Describe any methods used to assess it. Is there an estimate of variation within each group of data? Is the variance similar between the groups that are being statistically compared?	Not Applicable	NA

Sample definition and in-laboratory replication	Information included in the manuscript?	In which section is the information available? (Reagents and Tools Table, Materials and Methods, Figures, Data Availability Section)
In the figure legends: state number of times the experiment was **replicated** in laboratory.	Not Applicable	NA
In the figure legends: define whether data describe **technical or biological replicates**.	Not Applicable	NA

Ethics

Ethics	Information included in the manuscript?	In which section is the information available? (Reagents and Tools Table, Materials and Methods, Figures, Data Availability Section)
Studies involving **human participants**: State details of **authority granting ethics approval** (IRB or equivalent committee(s), provide reference number for approval.	Not Applicable	NA
Studies involving **human participants**: Include a statement confirming that **informed consent** was obtained from all subjects and that the experiments conformed to the principles set out in the WMA Declaration of Helsinki and the Department of Health and Human Services Belmont Report.	Not Applicable	NA
Studies involving **human participants:** For publication of **patient photos**, include a statement confirming that consent to publish was obtained.	Not Applicable	NA
Studies involving experimental **animals**: State details of **authority granting ethics approval** (IRB or equivalent committee(s), provide reference number for approval. Include a statement of compliance with ethical regulations.	Not Applicable	NA
Studies involving **specimen and field samples:** State if relevant **permits** obtained, provide details of authority approving study; if none were required, explain why.	Not Applicable	NA

Dual Use Research of Concern (DURC)	Information included in the manuscript?	In which section is the information available? (Reagents and Tools Table, Materials and Methods, Figures, Data Availability Section)
Could your study fall under dual use research restrictions? Please check biosecurity documents and list of **select agents and toxins** (CDC): https://www.selectagents.gov/sat/list.htm	Not Applicable	NA
If you used a select agent, is the security level of the lab appropriate and reported in the manuscript?	Not Applicable	NA
If a study is subject to dual use research of concern regulations, is the name of the **authority granting approval and reference number** for the regulatory approval provided in the manuscript?	Not Applicable	NA

Reporting

The MDAR framework recommends adoption of discipline-specific guidelines, established and endorsed through community initiatives. Journals have their own policy about requiring specific guidelines and recommendations to complement MDAR.

Adherence to community standards	Information included in the manuscript?	In which section is the information available? (Reagents and Tools Table, Materials and Methods, Figures, Data Availability Section)
State if relevant guidelines or checklists (e.g., **ICMJE, MIBBI, ARRIVE, PRISMA**) have been followed or provided.	Not Applicable	NA
For **tumor marker prognostic studies,** we recommend that you follow the **REMARK** reporting guidelines (see link list at top right). See author guidelines, under 'Reporting Guidelines'. Please confirm you have followed these guidelines.	Not Applicable	NA
For **phase II and III randomized controlled trials**, please refer to the **CONSORT** flow diagram (see link list at top right) and submit the CONSORT checklist (see link list at top right) with your submission. See author guidelines, under 'Reporting Guidelines'. Please confirm you have submitted this list.	Not Applicable	NA

Data Availability

Data availability	Information included in the manuscript?	In which section is the information available? (Reagents and Tools Table, Materials and Methods, Figures, Data Availability Section)
Have **primary datasets** been deposited according to the journal's guidelines (see 'Data Deposition' section) and the respective accession numbers provided in the Data Availability Section?	Not Applicable	NA
Were **human clinical and genomic datasets** deposited in a public access-controlled repository in accordance to ethical obligations to the patients and to the applicable consent agreement?	Not Applicable	NA
Are **computational models** that are central and integral to a study available without restrictions in a machine-readable form? Were the relevant accession numbers or links provided?	Yes	Data availability
If publicly available data were reused, provide the respective **data citations in the reference list**.	Yes	References
